# Counterintuitive Photochemistry of an Isolated Acridinyl Radical: ConPET via Preassembly, Solvated Electrons or a Long‐Lived Excited State?

**DOI:** 10.1002/anie.202506701

**Published:** 2025-07-02

**Authors:** Samuel J. Horsewill, Katherine M. M. Sharrock, Péter P. Fehér, Jack M. Woolley, Imre Pápai, Daniel J. Scott

**Affiliations:** ^1^ Department of Chemistry University of Bath Claverton Down Bath BA2 7AY UK; ^2^ Department of Chemistry University of Warwick Coventry CV4 7AL UK; ^3^ Institute of Organic Chemistry HUN‐REN Research Centre for Natural Sciences Magyar Tudósok Körútja 2 Budapest H‐1117 Hungary; ^4^ Department of Physics University of Warwick Coventry CV4 7AL UK

**Keywords:** Ab initio calculations, ConPET, Photoredox catalysis, Radical ions, Time‐resolved spectroscopy

## Abstract

Photoredox chemistry has seen a dramatic rise in popularity in recent years, but mechanistic understanding has persistently lagged behind reaction development itself. This is particularly true for the emerging area of consecutive photoinduced electron transfer (conPET), which has attracted both great interest due to its ability to activate inert substrates selectively and under mild conditions and continuing controversy over its mechanistic feasibility. We describe herein the isolation of the key radical intermediate state of an acridinium‐based conPET catalyst and detailed investigations of its photochemistry by a suite of (photo)reactivity, photoluminescence and transient absorption techniques, supported by computational studies. We observe strong wavelength and solvent dependencies in the reactivity profile, which correlate well with observations of a long‐lived, fluorescent excited state that would be compatible with diffusion‐limited reactivity. However, photoluminescence and transient absorption spectroscopies suggest that, counter‐intuitively, this state does not actually participate in reactivity. Instead, changes occur far faster than the diffusion limit, which provides strong, direct evidence for preassembly of the photocatalyst and substrate prior to photoexcitation. Further inspection also indicates parallel formation of solvated electrons, likely providing the major pathway under previously reported synthetic conditions, suggesting that otherwise competing rationales for conPET can in fact operate simultaneously.

## Graphical abstract

In this paper, we isolate the reduced form of a commonly used acridinium photocatalyst (acridinyl, **Acr^•^**) and investigate its photochemistry using transient absorption, analysed alongside TD‐DFT calculations. We show that despite the apparent long lifetime of the fluorescent state of **Acr^•^**, it is not relevant to reactivity. Instead, reactivity occurs through counterintuitive preassembly and solvated electron‐based mechanisms.

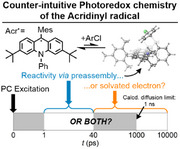

## Introduction

**Figure 1:**
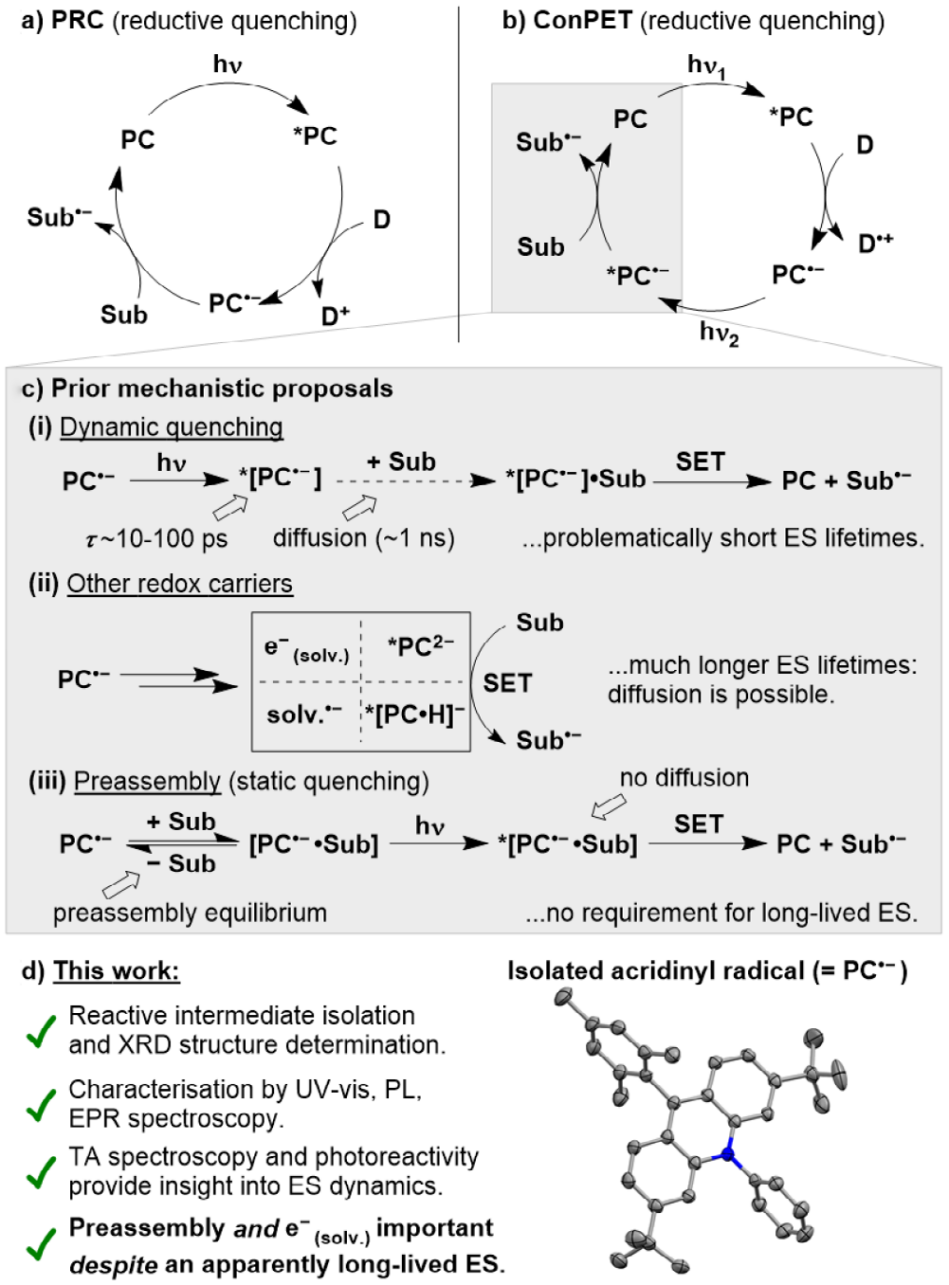
Generic mechanisms for a) standard PRC via reductive quenching and b) conPET catalysis, proceeding also via reductive quenching. Equivalent oxidative quenching mechanisms are also well established for both. Sub, substrate and D, electron donor. c) Competing hypotheses for the “true” mechanism of conPET catalysis; and d) isolation of a key PC^•−^ intermediate, **Acr^•^** and elucidation of its photoreactivity (this work).

Since its inception as a synthetic technique,^[^ ^1^, ^2^, ^3^ ^]^ photoredox catalysis (PRC) has gained significant attention as a sustainable alternative to traditional catalytic methods. In PRC, photons are used to change the redox properties of a photocatalyst (PC) such that in its excited state (ES, *PC) it undergoes electron transfer with a substrate (Figure 1a). In this way, inert substrates can be activated with high selectivity even under relatively mild reaction conditions.^[^ ^4^, ^5^, ^6^ ^]^ However, despite the rapid uptake of PRC in synthetic laboratories, it is challenging to study these reactions and gain mechanistic insight, in large part due to the high reactivity, short lifetimes and invisibility of critical transient radical intermediates to common reaction monitoring techniques such as NMR spectroscopy.

In recent years, a strong interest has emerged in multiple‐photon photoredox techniques, which are capable of performing especially challenging transformations by harnessing the energy of more than one photon during their catalytic cycles. Particularly prominent has been the development of “consecutive photoinduced electron transfer” (conPET), which allows reduction (or oxidation) of substrates that are normally very redox‐resistant (Figure 1b).^[^ ^7^ ^]^ In the generally proposed mechanism of conPET, the typical PRC cycle is supplemented by a second photoexcitation step, which transforms the key intermediate PC^•−^ into an extremely powerful photoreductant, *[PC^•−^] (for simplicity, this discussion will focus on the reductive quenching case; however, the analogous oxidative quenching mechanism proceeding via PC^•+^/*[PC^•+^] is also possible).^[^ ^8^ ^]^

The same key step—photoexcitation‐induced electron transfer from PC^•−^—also forms the basis for several other prominent PRC variants including “e‐PRC” (in which PC^•−^ is generated electrochemically rather than photochemically)^[^ ^6^, ^9^, ^10^ ^]^ and “multiPET” (which adds a third photoinduced electron transfer).^[^ ^11^, ^12^ ^]^ However, the feasibility of this step has proven controversial, provoking significant debate within the PRC community. Primarily, it has been argued that the key radical intermediates, PC^•−^, do not have long enough ES lifetimes to engage in productive bimolecular photoreactivity (Figure 1c, top). Most relevant PC^•−^ are organic doublets, which typically have very short ES lifetimes, on the order of tens of ps or less.^[^ ^6^, ^13^ ^]^ In contrast, diffusion‐limited interactions typically have much longer lifetimes on the order of 1 ns or more (although exact values will depend on concentration, etc.), which has led to a common “rule of thumb” that states that reactive species lifetimes of at least 1 ns are a prerequisite for an effective dynamic photochemical reaction step.^[^ ^14^, ^15^, ^16^, ^17^ ^]^

This has led to arguments that other pathways must instead be operative (Figure 1c, middle) and several options have been proposed, including complete loss of an electron from *[PC^•−^] to generate either a “free”, solvated electron (e^−^_(solv.)_) or a reduced form of the solvent (“solv.^•−^”), both of which are proposed to persist over longer timescales.^[^ ^18^, ^19^, ^20^, ^21^ ^]^ Others have suggested that PC^•−^ can be transformed in situ into closed‐shell species with similarly high reducing power to PC^•−^ but longer ES lifetimes.^[^ ^12^, ^22^, ^23^, ^24^, ^25^, ^26^ ^]^ In particular, Nocera and colleagues have argued for a Meisenheimer‐type complex, [NpMI·H]^−^, as the key intermediate in NpMI‐catalysed e‐PRC reactions, rather than NpMI^•−^.^[^ ^22^, ^23^ ^]^ Still others have argued that conPET/e‐PRC mechanisms are feasible, and that short ES lifetimes can be reconciled with productive photoreactivity by assuming reversible preassembly of the substrate and PC^•−^ prior to excitation (Figure 1c, bottom).^[^ ^27^, ^28^, ^29^ ^]^ This would remove the requirement for diffusion and allow static rather than dynamic ES quenching. Alternatively, a very recent report has suggested that for a PDI‐based *[PC^•−^] with a lifetime of 200–250 ps, diffusion‐limited reactivity is possible, despite this lifetime falling below the 1 ns “rule of thumb”.^[^ ^30^ ^]^

Since their first introduction in 2004,^[^ ^31^, ^32^, ^33^ ^]^ PC systems based on the acridinium backbone have frequently been applied as potent photo‐oxidants in PRC.^[^ ^34^ ^]^ More recently, these catalysts have also played a central role in the development of conPET catalysis, with particular focus on the specific derivative 3,6‐di‐*tert‐*butyl‐9‐mesityl‐10‐phenyl‐acridin‐10‐ium (**Acr^+^**).^[^ ^35^, ^36^, ^37^, ^38^, ^39^, ^40^ ^]^ However, detailed studies into the photoreactivity of the key PC^•−^ intermediate, **Acr^•^** (Figure 1d), are conspicuously lacking, especially in contrast to other conPET/e‐PRC catalysts such as NpMI,^[^ ^22^, ^23^, ^41^ ^]^ DCA,^[^ ^41^, ^42^ ^]^ or PDI.^[^ ^30^, ^43^, ^44^ ^]^ As part of their initial report on **Acr^+^**‐based conPET, the Nicewicz group performed preliminary mechanistic investigations on **Acr^•^** generated in situ; however, the conclusions were tentative, and given the broader controversy around conPET mechanisms, there is a clear need for further study.^[^ ^35^, ^36^, ^37^, ^38^, ^39^ ^]^

We, and others, have recently reported the preparative synthesis of several key PC^•−^ states, which have provided detailed mechanistic insight into their deceptively complex (photo)catalytic reactivity.^[^ ^21^, ^41^, ^45^, ^46^, ^47^ ^]^ We anticipated that a similar methodology could be applied to **Acr^•^** to provide missing mechanistic detail. We thus report herein the first preparative synthesis and isolation of the reduced acridinium photocatalyst **Acr^•^**, its full characterisation (including solid‐state molecular structure) and detailed spectroscopic reactivity studies. These confirm the strong photoreducing ability of **Acr^•^**, subject to clear dependencies on solvent and excitation wavelength that directly correlate with observations of an unusually long‐lived fluorescent ES. Crucially, however, we also show that this correlation is misleading, with transient spectroscopy revealing that photoreactivity is unrelated to this emissive state and can in fact occur either on very short timeframes via preassembly and higher energy ESs, or on longer timeframes through release of solvated electrons, with the latter likely being the major pathway under the conditions used in previous synthetic reports. These results challenge several of the standard assumptions made when trying to model PRC mechanisms.

## Results and Discussion

To our knowledge, the isolation of a simple, neutral acridinyl radical such as **Acr^•^** has not previously been achieved (although there is some structural similarity to the helicene radical PC^•−^ state *^n^* Pr‐DMQA^•^ recently isolated by the Gianetti group).^[^ ^21^ ^]^ However, the isolation of a related diradical and distonic radical cation have both been reported very recently,^[^ ^48^, ^49^ ^]^ as has the synthesis of isoelectronic (but much less reducing) xanthyl radicals.^[^ ^50^ ^]^ This, combined with the recent isolation of other formal PC^•−^ states, suggests that isolation of **Acr^•^** should be feasible.

### Synthesis and Characterisation

**Scheme 1:**
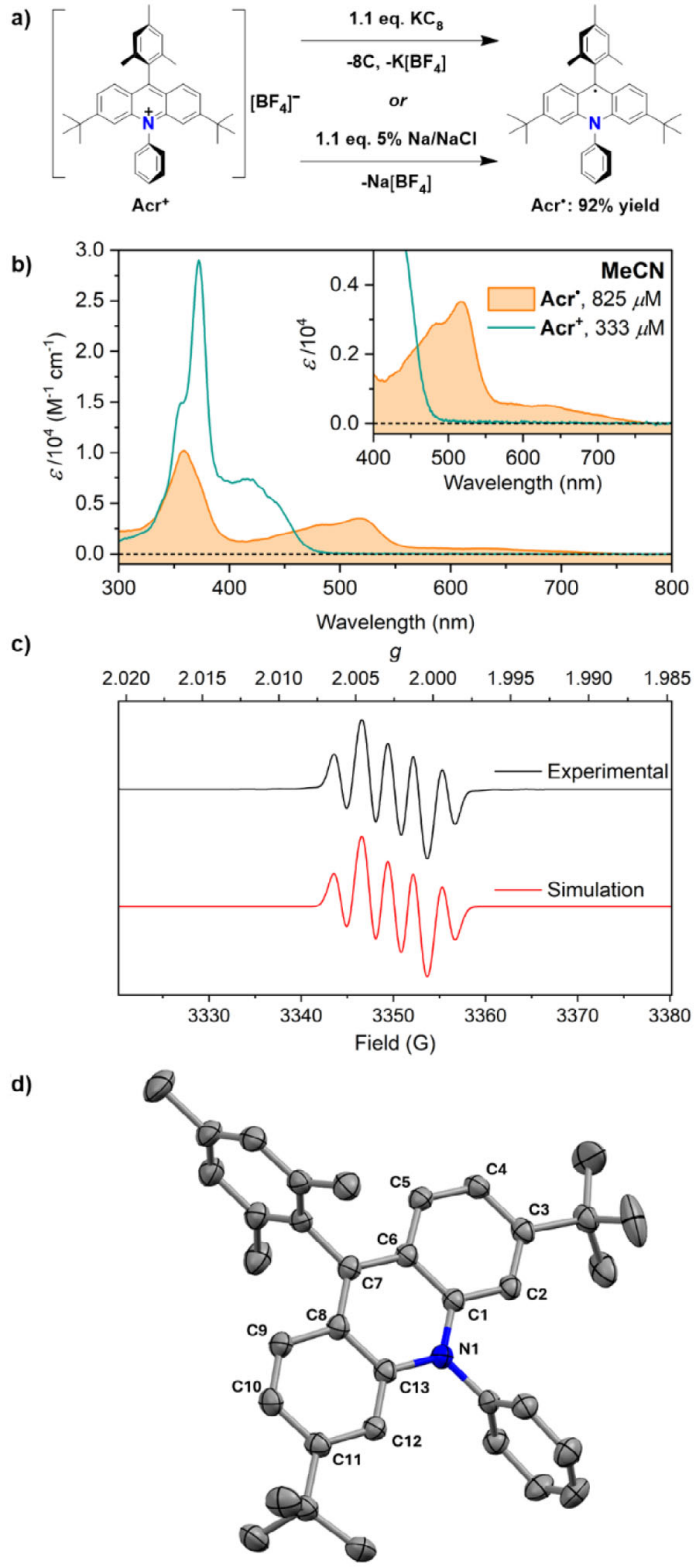
a) Synthesis of **Acr^•^** via KC_8_ or 5% Na/NaCl reduction of [**Acr**]^+^[BF_4_]^−^. Quoted yield is for the 5% Na/NaCl reduction. b) UV–vis spectra of **Acr^•^** (orange) and [**Acr**]^+^[BF_4_]^−^ (teal) recorded in MeCN solution at 298 K, at 825 µM and 333 µM concentrations, respectively. Inset: expansion showing the absorptions of **Acr^•^** more clearly. c) EPR spectrum of **Acr^•^** recorded in toluene solution at 298 K at 200 µM concentration. d) Solid‐state molecular structure of **Acr^•^** as crystallised from a concentrated 1:1 hexane/pentane mixture cooled to −35 °C. Half a hexane molecule, H atoms and positional disorder modelled in the *tert‐*butyl group positions omitted for clarity. Thermal ellipsoids drawn at 50% probability.

Building on our previous methodology, THF solutions of the closed‐shell precursor [**Acr**]^+^[BF_4_]**^−^** were stirred with 1.1 equiv of either KC_8_ or of Na dispersed on NaCl (Na/NaCl, 5% w/w) and then extracted into toluene. Purification was achieved either by sublimation under static vacuum or by crystallisation from pentane, to furnish small, dark‐red crystals of **Acr^•^** in excellent yield (Scheme 1a).

**Acr^•^** is NMR silent, as expected for a delocalised organic radical, but shows strong absorptions in its UV–vis absorption spectrum up to approximately 550 nm (Scheme 1b). This spectrum, which is consistent with previous in situ measurements,^[^ ^35^ ^]^ is essentially identical in both MeCN and THF and is not affected by changes in concentration or temperature (Figures S7–S9).

The X‐band EPR spectrum of **Acr^•^** is also in agreement with a prior in situ observation, although this was not previously simulated.^[^ ^35^ ^]^ The spectrum exhibits hyperfine coupling to one ^14^N nucleus (*A* = 6.851 MHz) and to two equivalent ^1^H nuclei (*A* = 9.294 MHz). These are assigned to the protons at the C9 positions by comparison to the calculated spin density (Scheme 1c).^[^ ^31^ ^]^ No coupling to the two other ^1^H environments of the central acridinyl moiety is observed due to low spin density on these carbon atoms. Using a lower modulation amplitude, a hyperfine structure was observed with a complex mixture of very small coupling constants (*A* ≈ 0.3–0.4 MHz, see Figures S5 and S6). However, these components could not be fully simulated as they were at the detection limit of the spectrometer and collection program used. We attribute this contribution to minor interactions with the protons of the Ph, Mes and/or ^t^Bu moieties, which do not have high spin density.

Cooling a concentrated solution of **Acr^•^** in a 1:1 mixture of hexane and pentane to −35 °C allowed single crystals suitable for X‐ray diffraction to be grown, and the resulting solid‐state molecular structure is shown in Scheme 1d. It features a single acridinyl molecule in the asymmetric unit (as well as half a hexane molecule) and no counterion, consistent with the expected neutral radical. The bond lengths around the central ring of the acridinyl moiety are fully consistent with the **Acr^•^** oxidation state, with significantly lengthened internal N─C bonds (1.397(2)–1.401(2) Å) compared to similar structures in the acridinium oxidation state (ca. 1.36–1.38 Å; see Figure S63 and Table S3 for further information).^[^ ^51^, ^52^ ^]^ This is consistent with the expected lowering of C─N bond order upon reduction.

Of particular interest are the twist angles between the three ring systems in **Acr^•^** as it has been proposed that intramolecular charge transfer between these rings plays a key role in extending the ES lifetime for both **Acr^+^** and **Acr^•^**. Specifically, it has been suggested that electron transfer from the C7‐bound mesityl group to the acridinium core (for **Acr^+^**) or from the acridinyl core to the *N*‐bound phenyl group (for **Acr^•^**) leads to charge‐transfer (CT) excited states that are long‐lived due the near‐orthogonality of these rings, which limits the orbital overlap required for back‐electron‐transfer.^[^ ^35^ ^]^ In the solid‐state molecular structure of **Acr^•^**, the acridinyl and phenyl planes are indeed near‐perpendicular, with an interplanar angle of 87.1°. This is very similar to the equivalent angles observed in similar PCs in the acridinium oxidation state (ca. 87–89°; see Figure S63 and Table S3).^[^ ^51^, ^52^ ^]^ In contrast, the mesityl ring is significantly closer to being perpendicular to the acridine/acridinyl core in **Acr^•^** (88.3°) than is observed in these acridinium cations (ca. 75–81°).

### Photoreactivity Studies

With isolated **Acr^•^** in hand, we turned our attention to the investigation of its (photo)reactivity, with aryl chlorides being chosen as model substrates. Reductive functionalisation of aryl chlorides is synthetically attractive as these substrates are widely available and usually inexpensive. However, their transformations are normally challenging due to strong C─Cl bonds and very negative reduction potentials. As such, reductive functionalisation of Ar─Cl bonds has become a common benchmark reaction in conPET catalysis, including for both the initial examples of conPET involving **Acr^•^** as an intermediate and our own previous mechanistic studies using other isolated PC^•−^.^[^ ^35^, ^41^, ^47^ ^]^

In line with our previous work, we chose to trap the aryl radicals generated upon ArCl reduction using P(OMe)_3_ to form aryl phosphonate products ArPO(OMe)_2_, in order to easily monitor the reactions by ^31^P{^1^H} NMR spectroscopy. Several different excitation wavelengths were used (395, 455 and 530 nm) as we have previously shown that this can dramatically affect the photoreactivity of other authentic PC^•−^ states, and such wavelength dependence is a topic of considerable current interest (vide infra).^[^ ^13^ ^]^ Studies were also performed in both MeCN (which has been the typical solvent for previous **Acr^+^**‐based conPET reactions) and THF (which has been used for previous in situ photophysical characterisation of **Acr^•^**).^[^ ^35^ ^]^

We first investigated the photostability of **Acr^•^** under these conditions by UV–vis and NMR spectroscopy, both with and without P(OMe)_3_ present. In MeCN solution, the radical was photostable and largely unreactive for at least 16 h at all three wavelengths used. However, in THF, good photostability was only observed at 455 and 530 nm, with decomposition gradually occurring over several hours under irradiation with 395 nm LEDs. The decomposition product(s) exhibited a complex ^1^H NMR spectrum and a UV–vis absorption maximum at 468 nm, neither of which is consistent with net oxidation to **Acr^+^**. This decomposition did not appear to be affected by the presence of P(OMe)_3_, and no ^31^P containing NMR‐active products were observed. Control studies to probe the cause of this decomposition are described in the Supporting Information (Section 3.2).

Having established the photostability of **Acr^•^** under most of the conditions of interest, its photoreactivity towards a small family of aryl chloride substrates with electronically distinct *para* substituents was then investigated. No reactions were observed in the dark. However, under 395 nm LEDs in MeCN, the reduction of all five substrates was observed (Table 1, top). Although lower conversions were generally obtained for more electron‐rich substrates, in line with expectations, significant reduction was achieved even with the very electron‐rich 4‐chloroaniline, evidencing a very high reductive strength for **Acr^•^** upon photoirradiation. In contrast, at longer wavelengths, significantly reduced conversions were observed for all substrates, with appreciable reactivity effectively being limited to more electron‐poor substrates such as 4‐chlorobenzonitrile. This is despite **Acr^•^** absorbing well at both 455 and 530 nm (cf. Scheme 1b).

**Table 1:** Conversion of aryl chlorides to aryl phosphonate esters via reduction to aryl radicals using **Acr^•^** and trapping with P(OMe)_3_.^a)^

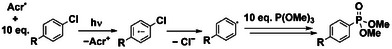				
		Conv.		
Solvent	*R*	395 nm (%)	455 nm (%)	530 nm (%)
MeCN	NH_2_	103	n.d.	n.d.
	OMe	284^b)^	10	n.d.
	H	196	59	n.d.
	CO_2_Me	389	116	15
	CN	882	352	375
THF	NH_2_	9^c)^, ^d)^	−	−
	OMe	15^b)^, ^d)^	−	−
	H	23^c)^, ^d)^	n.d.	n.d.
	CO_2_Me	30^c)^, ^d)^	n.d.	n.d.
	CN	50^c)^, ^d)^, 105^c)^, ^d)^, ^e)^	47^d)^	15^d)^

^a)^ **Acr^•^**, 1.7 mM (MeCN), 5 mM (THF); R‐C_6_H_4_Cl, 50 mM; P(OMe)_3_, 50 mM; 16 h, rt. Yields are given versus **Acr^•^**.

^b)^ Unidentified side‐products were produced in addition to the expected phosphonate product.

^c)^ **Acr^•^** is not fully photostable under 395 nm irradiation in THF.

^d)^ HPO(OMe)_2_ was observed as a side‐product.

^e)^ **Acr^•^** at 1.7 mM instead of 5 mM concentration.

Despite being designed as stoichiometric reactions with no other sacrificial reductant, conversions greater than 100% were consistently observed in MeCN, with almost 9 turnovers being observed at 395 nm for the most electronically activated substrate, 4‐chlorobenzonitrile. This is consistent with our previous investigations of other PC^•−^ using the same methodology, and we again attribute this to re‐reduction of PC to PC^•−^ (in this case, of **Acr^+^** to **Acr^•^**) by the initial radical trapping intermediate ArP^•^(OMe)_3_ (see Supporting Information, Section 3.4).^[^ ^41^, ^47^ ^]^

In contrast, identical reactions in THF gave significantly lower conversions, typically by around an order of magnitude (Table 1, bottom). As in MeCN, the reactivity displayed a strong wavelength dependence, with only 395 nm irradiation allowing reduction of more electron‐rich substrates (despite the photodecomposition occurring at this wavelength). Reactions in THF were also noticeably less clean. In all cases, where the expected ArPO(OMe)_2_ product was observed by ^31^P{^1^H} NMR spectroscopy, a significant secondary product was also observed, which can be assigned as dimethylphosphite (HPO(OMe)_2_) on the basis of ^31^P{^1^H}, ^31^P and ^1^H NMR data (see Supporting Information, Section 3.3, for relevant spectra and discussion of possible formation mechanisms).

### Fluorescence Spectroscopy

The reactivity studies above confirm the ability of **Acr^•^** to effect reduction of challenging substrates upon photoirradiation. However, they cannot on their own account for how this reactivity occurs (vide supra). To shed more light on the latter question, more detailed spectroscopic characterisation was carried out, beginning with steady‐state fluorescence experiments.

**Figure 2:**
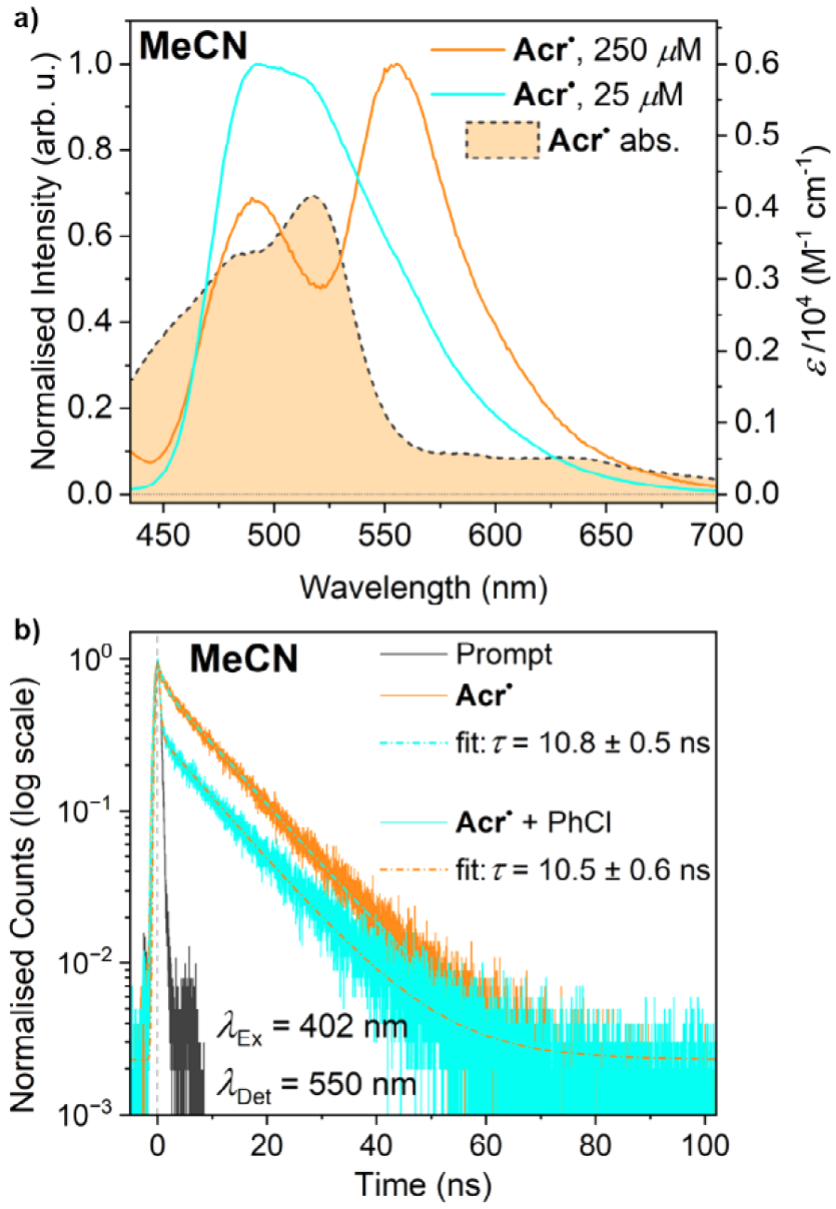
a) Steady‐state fluorescence spectra of **Acr^•^** at different concentrations under 420 nm excitation. Spectra are normalised to their global maxima. The absorbance spectrum of **Acr^•^** is also shown for reference. b) Fluorescence decay traces for **Acr^•^** (500 µM) and for a mixture of **Acr^•^** (500 µM) and PhCl (50 mM). All spectra recorded in MeCN at 298 K.

Previous studies on in situ generated **Acr^•^** have suggested the existence of an emissive ES.^[^ ^35^, ^40^ ^]^ However, it is known that previous attributions of fluorescent behaviour to other organic PC^•−^ (e.g., DCA^•−^)^[^ ^53^, ^54^, ^55^ ^]^ have been erroneous, with observed emissions instead being caused by decomposition products (e.g., due to exposure to air), and it has recently been suggested that the apparent fluorescence of **Acr^•^** may be a similar misinterpretation.^[^ ^13^ ^]^ Nevertheless, in our hands, steady‐state fluorescence experiments on isolated **Acr^•^** in MeCN under rigorously oxygen‐ and water‐free conditions gave reproducible emission spectra (Figures 2a and Figure S51). Experiments at relatively high **Acr^•^** concentration show two maxima at 490 and 555 nm, consistent with previous reports where this was attributed to the existence of two different emissive states.^[^ ^35^ ^]^ However, experiments at lower **Acr^•^** concentration show only a single maximum, which is inconsistent with this prior interpretation. In fact, the observation of two maxima at higher concentrations appears to be due to simple inner filter effects, with the apparent drop in emission around 520 nm being attributable to the presence of a significant **Acr^•^** absorbance maximum at this wavelength (Figure 2a). Interestingly, the “true”, lower‐concentration emission spectrum of **Acr^•^** is very similar to that of **Acr^+^** (vide infra).

The observation of fluorescence from an organic PC^•−^ state is highly unusual in the context of PRC. However, it should be noted that acridinyl radicals can be considered a subset of the broader family of triarylmethyl radicals, which have been widely studied for their emissive properties, including examples showing both higher excited state emission (vide infra)^[^ ^56^ ^]^ and exceptionally long ES lifetimes of up to 400 ns.^[^ ^57^ ^]^ In particular, a family of xanthyl radicals, whose core structure is isoelectronic to that of **Acr^•^,** has very recently been reported to be fluorescent from either localised or CT ESs, with lifetimes between 1 and 20 ns.^[^ ^50^ ^]^

The lifetime of the emissive **Acr^•^**‐derived ES in MeCN was quantified by time‐resolved fluorescence measurements, which revealed a monoexponential fluorescence decay with a lifetime of 10.8 ns (Figure 2b). Notably, this is significantly longer than the 1 ns “rule of thumb” lifetime noted above (which agrees well with our own calculations of a 1.0 ns diffusion lifetime in our reactions; see Supporting Information, Section 6.3), suggesting that this emissive state, hereafter labelled *[**Acr^•^**]^EM^, should be long‐lived enough to engage in a diffusion‐limited bimolecular reaction with ArCl. Also notable is the fact that this emission is strongly solvent‐dependent, with no fluorescence observed in THF solution, despite the UV–vis spectra not exhibiting any significant differences in the two solvents (see Figure S7). Similarly, the emission is strongly wavelength dependent, with no fluorescence observed upon irradiation at 530 nm, despite the strong absorbance of **Acr^•^** at this wavelength, suggesting that this could represent emission from a high energy ES (vide infra for additional discussion; see also Supporting Information, Section 6.1).

It is conspicuous that the existence of the *[**Acr^•^**]^EM^ state appears to correlate closely with the photoreactivity observed in the previous section, with good conversion of all substrates being observed under conditions where *[**Acr^•^**]^EM^ is accessible (MeCN, 395 nm irradiation) and greatly diminished performance when it is not (THF or longer irradiation wavelengths). Thus, at this point in our study, there appeared to be a refreshingly simple explanation for the conPET reactivity observed using **Acr^+^**: that unlike most other PC^•−^, **Acr^•^** possesses a fluorescent ES (*[**Acr^•^**]^EM^) that is long‐lived enough to engage in productive, diffusion‐limited interactions with external substrates. In this model, the solvent‐ and wavelength‐sensitivity of this ES would neatly explain the solvent‐ and wavelength‐dependence of the observed photoreactivity.

In an attempt to confirm this explanation, the time‐resolved fluorescence measurements were repeated in the presence of a large excess of the model substrate PhCl (whose concentration was matched to that used in the earlier reactivity studies: 50 mM). Under a dynamic quenching regime, this should lead to a significant reduction in the fluorescence lifetime. However, to our surprise, no significant change in lifetime was observed (within error: 10.5 ± 0.5 ns versus 10.8 ± 0.6 ns), although a qualitative comparison indicated that the initial intensity of emission was significantly diminished (see Figure S53), suggesting that the initial population of the emissive state was reduced in the presence of substrate. It should be noted that no significant difference in the steady‐state absorption spectrum of **Acr^•^** is observed in the presence of substrate, which might otherwise explain this discrepancy (see Figures S9–S12).

### Transient Absorption Spectroscopy

The time‐resolved fluorescence measurements suggest that *[**Acr^•^**]^EM^ is in fact not efficiently quenched by PhCl. To better understand this surprising result, we turned to ultrafast transient absorption spectroscopy (TA), to gain further insight into the excited‐state dynamics of **Acr^•^**. To provide a comprehensive set of results, measurements were performed in both MeCN and THF, both in the presence and absence of PhCl (50 mM), and under excitation at wavelengths where PhCl either was or was not transformed during reactivity studies (395 and 530 nm, respectively; cf. Table 1).

All traces were background‐corrected before chirp correction, deconvolution and fitting of the resultant matrices under a sequential model (see Supporting Information, Sections 5.3 and 6.2, for full details, including additional discussion of possible sub‐ps and/or trace ns features not considered here). It should be emphasised that in all cases the sample was translated in two dimensions between laser pulses throughout the experiment to avoid any persistent contributions from photodecomposition, and we observed no bulk change in absorbance. We can therefore rule out any contributions to the transient behaviour from photodecomposition products.

**Figure 3:**
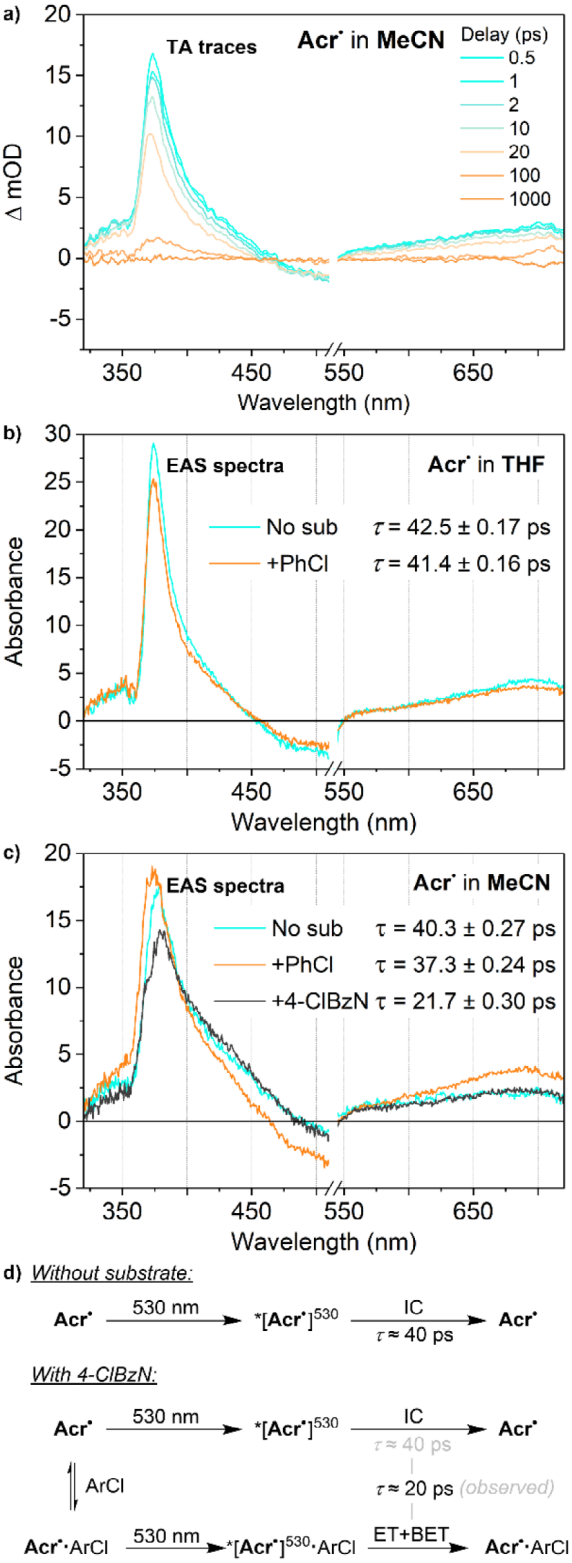
a) TA spectra of **Acr^•^** in MeCN at selected time points following excitation by 530 nm light; and deconvoluted TA evolution‐associated spectra (EAS) following excitation at 530 nm in b) THF solution or c) MeCN solution. Concentrations: **Acr^•^**, 1 mM; chlorobenzene (PhCl) and 4‐chloro‐benzonitrile (4‐ClBzN), 50 mM. d) Schematic illustration of the photochemical processes involved. IC, internal conversion; (B)ET, (back‐)electron transfer.

Beginning with 530 nm irradiation, we observed only a single component in the TA spectra in both THF and MeCN (Figure 3). This component appears very similar in both solvents, featuring a ground‐state bleach between 450 and 550 nm, alongside excited state absorptions at 375 and 700 nm. This component, hereafter labelled *[**Acr^•^**]^530^, has a relatively short lifetime (THF, *τ* = 42.5 ps; MeCN, *τ* = 40.3 ps) consistent with other organic *[PC^•−^].^[^ ^35^, ^42^, ^44^ ^]^

The shape of the TA traces for *[**Acr^•^**]^530^ was not noticeably affected by the presence of substrate, and only very slight decreases in lifetime were detected in the presence of PhCl (from 42.5 to 41.4 ps in THF; from 40.3 to 37.3 ps in MeCN), consistent with the lack of reactivity towards PhCl observed during photoreactivity studies at 530 nm. However, a much more significant reduction in lifetime was observed upon addition of the more activated, electron‐poor substrate 4‐chlorobenzonitrile (21.7 ps in MeCN). This suggests efficient quenching of the excited state by the substrate on a tens of ps timescale, which should not be possible under a diffusion‐limited regime and instead points towards a static quenching model (vide infra).

**Figure 4:**
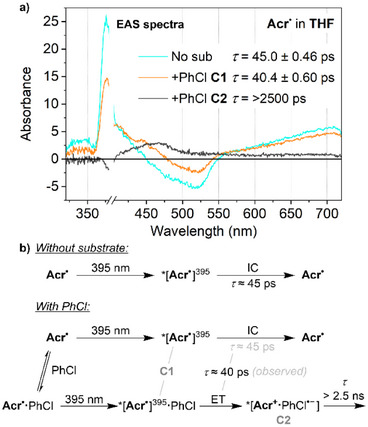
a) Deconvoluted TA EAS spectra following excitation at 395 nm in THF solution. Where multiple components are observed, they are denoted as C1 and C2, with C1 transforming into C2. Concentrations: **Acr^•^**, 1 mM; chlorobenzene (PhCl), 50 mM. b) Schematic illustration of the photochemical processes involved. IC, internal conversion; ET, electron transfer.

Very similar TA behaviour was observed in THF upon irradiation of **Acr^•^** at 395 nm. This again provided only a single transient feature, hereafter referred to as *[**Acr^•^**]^395^ (Figure 4). The TA trace and lifetime of *[**Acr^•^**]^395^ are very similar to those of *[**Acr^•^**]^530^ (*τ* = 45.0 ps versus 42.5 ps), and it is therefore possible that they are in fact the same ES, potentially being generated via very rapid relaxation (*τ* < 1 ps) of two different initial excitations. However, significant differences are observed in the presence of substrate, and so we tentatively assign *[**Acr^•^**]^395^ and *[**Acr^•^**]^530^ to be two distinct ESs, albeit with enough electronic similarities to yield very similar TA transients (see Supporting Information, Section 5.3, for additional discussion). Specifically, unlike for *[**Acr^•^**]^530^, TA indicates a reduced lifetime for *[**Acr^•^**]^395^ in the presence of PhCl (from 45.0 to 40.4 ps), and evolution into a second transient state with a much longer lifetime that exceeds the experimental timescale (>2500 ps, black trace in Figure 4a). The fact that this component is not observed in the absence of substrate suggests direct involvement of PhCl and we therefore assign this state to the initial photoproduct formed via electron transfer from **Acr^•^** to PhCl, *[**Acr^+^·**PhCl^•−^]. This conclusion is consistent with both computational investigations (vide infra) and reactivity studies (PhCl reduction observed under 395 nm but not 530 nm irradiation; vide supra). Again, it is clear that the presence of substrate leads to changes in ES behaviour on a tens of ps timescale.

**Figure 5:**
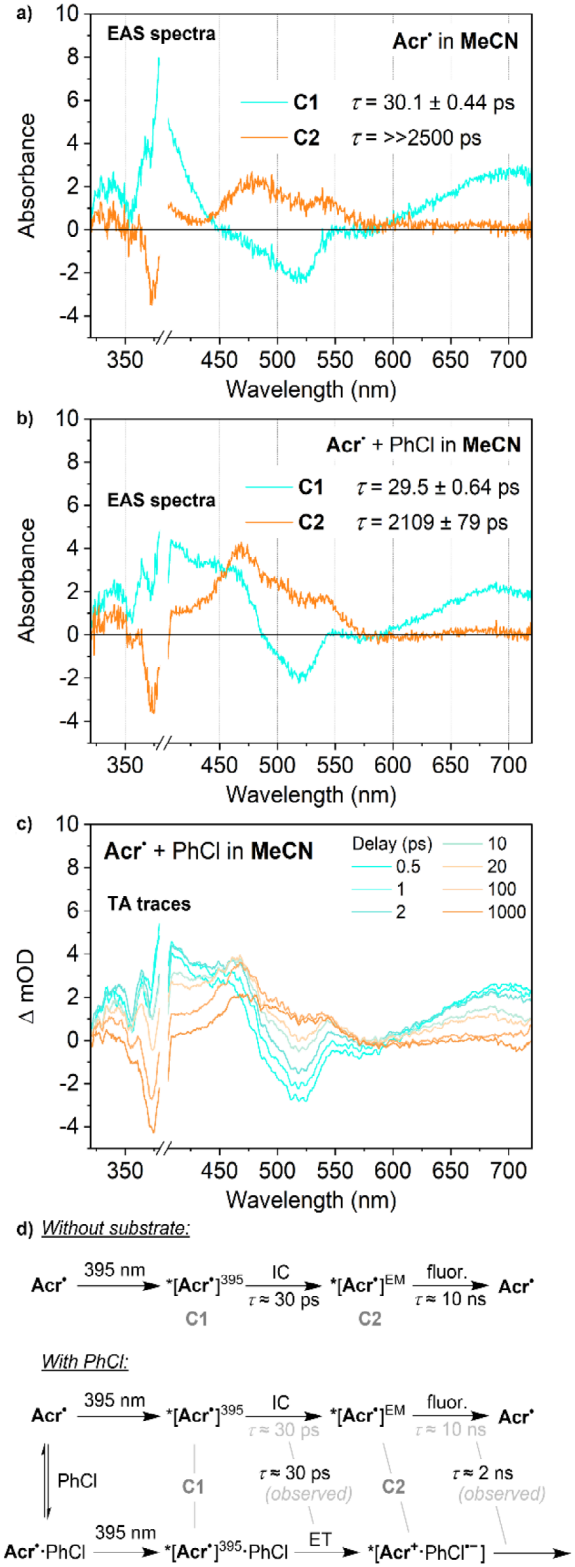
Deconvoluted TA EAS spectra a) for **Acr^•^** following excitation at 395 nm in MeCN solution and b) in the presence of chlorobenzene. Where multiple components are observed, they are denoted as C1 and C2, with C1 transforming into C2. c) TA spectra of **Acr^•^** in the presence of chlorobenzene under the same conditions at selected time points. Concentrations: **Acr^•^**, 1 mM; chlorobenzene (PhCl), 50 mM. d) Schematic illustration of the photochemical processes involved. IC, internal conversion; ET, electron transfer; fluor., fluorescence.

More complex behaviour was observed at 395 nm in MeCN, with irradiation of **Acr^•^** resulting in a transient spectrum with two components. The first of these is a short‐lived state very similar to that observed in THF at the same wavelength, which is therefore assigned as the same ES, *[**Acr^•^**]^395^ (Figure 5a). However, this state was observed to have an appreciably shorter lifetime than in THF (30.1 ps versus 45.0 ps) and to evolve into a second transient state rather than relax directly to the ground state. This second state features two new absorption maxima at 480 and 530 nm and has a much longer lifetime. This lifetime is significantly longer than the experimental measurement window (>>2.5 ns) and so cannot be quantified with high confidence; however, it is estimated to be on the order of ca. 10 ns. Given the similarity between this value and the lifetime measured by time‐resolved fluorescence experiments (10.8 ns), and the fact that it is only observed under the conditions in which emission is also observed (MeCN, 395 nm irradiation), this state can be assigned fairly confidently as the emissive state *[**Acr^•^**]^EM^.

Finally, the TA behaviour of **Acr^•^** in the presence of PhCl was investigated under these same conditions, and modelling of the TA data again indicated a two‐component system with an initial, short‐lived transient evolving into one longer‐lived component. (Figure 5b). The shape and lifetime of the former are mostly unchanged, so this is assigned as the same ES, *[**Acr^•^**]^395^. However, significant differences were observed for the longer‐lived trace. In fact, close inspection of the data revealed that this trace actually corresponds to two different transient species with different lifetimes. While the quality of the TA data does not allow for a quantitative deconvolution, visual inspection confirms that the shape of the trace varies systematically over time (Figure 5c).

Specifically, this trace features two primary absorbance maxima: at ca. 470 and 540 nm. The former can be seen to diminish significantly over several hundred ps, with no concomitant change in the latter. The remaining trace closely resembles the long‐lived transient observed in the absence of PhCl and decays similarly slowly, so is again assigned to the emissive ES *[**Acr^•^**]^EM^. Meanwhile, the other contribution is a good match for the long‐lived trace observed for **Acr** ^•^ + PhCl in THF (Figure 4a, maximum at 470 nm), so is similarly assigned as the initial photoproduct *[**Acr^+^·**PhCl^•−^]. We suggest that these two states could arise separately from excitation of **Acr^•^** that either is or is not preassembled with PhCl (Figure 5d and vide infra).

Note that in this final experiment, the short‐lived TA trace assigned to *[**Acr^•^**]^395^ also shows higher‐than‐expected absorbance at around 450 nm (cf. TA in the absence of PhCl; Figure 5a versus 5b). This can also be explained through incomplete deconvolution of the TA spectra for *[**Acr^•^**]^395^ and *[**Acr^+^·**PhCl^•−^].

### Electronic Structure Calculations

**Figure 6:**
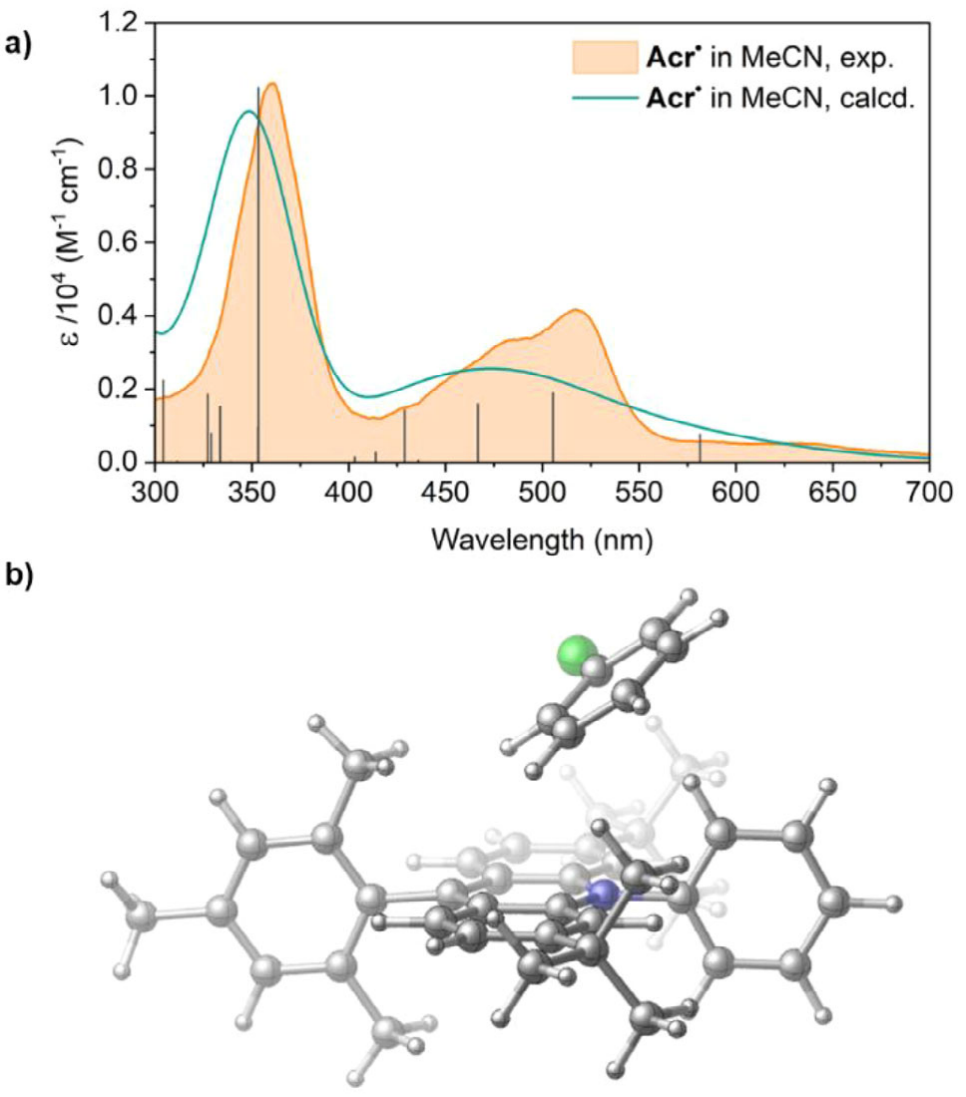
a) Experimental (orange) and calculated (teal) UV–vis spectra of **Acr^•^** in MeCN solution, with calculated transitions shown as black bars. b) Structure of the lowest‐energy calculated [**Acr^•^** ·PhCl] preassembly in MeCN solution.

To assist with analysis of the transient absorption data, we performed time‐dependent density functional theory (TD‐DFT) calculations on an optimised structure of **Acr^•^**, identifying the first 30 excited states and predicting their energies and oscillator strengths (Table 2; for full data see Table S15). We used the B3LYP‐D3(BJ)/TZVP level of DFT with the SMD implicit solvation model,^[^ ^58^, ^59^, ^60^, ^61^ ^]^ using the Gaussian16 software package together with Multifwn for hole‐electron analysis to further characterise the doublet radical states below ∼3.5 eV (above ∼350 nm) and ORCA for relaxation rates (see Supporting Information, Section 8.1, for further details).^[^ ^62^, ^63^, ^64^, ^65^ ^]^ The calculated absorption spectra for **Acr^•^** are an excellent match for the experimental data in both MeCN and THF (Figures 6a, S67, and S68). Quartet states were considered as well as doublets but were found to be too high in energy to be relevant (see Table S15). On this basis, we are able to suggest that excitations at 395 and 530 nm should initially generate primarily the D_9_ and D_2_ states, respectively. This allows for a tentative correlation of the transient *[**Acr^•^**]^530^ with D_2_ and *[**Acr^•^**]^395^ with D_9_ (although fs relaxation to lower‐energy states is also plausible; see Supporting Information, Section 5.3). Interestingly, these are both calculated to be localised excitations rather than CT states, as has previously been proposed for the active **Acr^•^** ES.^[^ ^35^ ^]^

**Table 2:** Calculated ES energies and oscillator strengths (*f*) from TD‐DFT in MeCN solution for **Acr^•^** and for preassembly complex [**Acr^•^** ·PhCl].

Calculated D_0_→D*_n_* for **Acr^•^**				Calculated D_0_→D*_n_* for [**Acr^•^** ·PhCl]			
D*_n_*	Δ*E* (eV)	*λ* (nm)	*f*_0n_ (10^2^)	*f*_0n_ (10^2^)	*λ* (nm)	Δ*E* (eV)	D*_n_*
D_1_	2.132	582	1.93	2.26	567	2.188	D_1_
D_2_	2.453	505	4.73	3.77	502	2.471	D_2_
*D_3_*	*2.486*	*499*	*0*	*0.36*	*491*	*2.525*	*D_3_*
D_4_	2.656	467	3.99	3.24	479	2.586	D_4_
–	–	–	–	**0.71**	**469**	**2.646**	**D_PhCl,1_**
–	–	–	–	**0.10**	**459**	**2.699**	**D_PhCl,2_**
*D_5_*	*2.844*	*436*	*0.14*	*0.24*	*431*	*2.876*	*D_5_*
D_6_	2.891	429	3.57	2.14	426	2.909	D_6_
D_7_	2.995	414	0.68	0.30	406	3.057	D_7_
*D_8_*	*3.076*	*403*	*0.36*	*0.25*	*402*	*3.082*	*D_8_*
D_9_	3.509	353	25.60	18.04	361	3.438	D_9_
*D_10_*	*3.511*	*353*	*2.38*	*5.33*	*355*	*3.4973*	*D_10_*

Entries in **bold** denote intermolecular charge transfer states, and those in *italics* denote intramolecular charge transfer states. Blank lines included to highlight similarities between **Acr** ^•^ and [**Acr** ^•^·PhCl].

As TA investigations suggest static rather than dynamic quenching behaviour, we also decided to investigate the potential for preassembly between **Acr^•^** and PhCl in silico. Screening of the ground state potential energy surface revealed a number of local minima corresponding to different weakly bound **Acr^•^⋅**PhCl adduct structures, with the lowest energy lying at +2.4 kcal mol^−1^ in MeCN (+3.0 kcal mol^−1^ in THF) in free energy with respect to the dissociated state. Though slightly endergonic, these states are clearly accessible in solution, so consistent with a reversible equilibrium in which this preassembled structure is a minor component (alongside adducts with other geometries; see Supporting Information, Section 8.1). This lowest energy conformer has PhCl located above the N atom of the acridine core featuring aryl–aryl contacts with acridine (Figure 6b). Other low energy structures feature similar π–π interactions (see Supporting Information, Section 8.1.1, for details).

To understand the photochemical consequences of this preinteraction with PhCl, we then performed TD‐DFT calculations using this optimised geometry. The resulting ES energies and oscillator strengths are shown on the right in Table 2 (see also Tables S7 and S8) and can be summarised with two key conclusions. First: the presence of PhCl has relatively little effect on the existing **Acr^•^** transitions, either in terms of energy or oscillator strength. However, second: it does lead to the emergence of two new ESs, D_PhCl,1_ and D_PhCl,2_, both of which can be characterised as having intermolecular CT character, i.e., *[**Acr^+^·**PhCl^•−^] (see Table S11 and Figure S70).

D_PhCl,1_ and D_PhCl,2_ both have very low oscillator strengths, so cannot be accessed efficiently via direct photoexcitation, which is consistent with the lack of change to steady‐state UV–vis spectra observed upon PhCl addition. However, it seems likely that they would be easily accessible via relaxation of higher energy excited states. Crucially, the relative energies of the states involved mean that this relaxation would be possible for excitation at 395 nm (as the **Acr^•^** D_9_ state is significantly higher in energy than D_PhCl,1_ or D_PhCl,2_) but not at 530 nm (as the **Acr^•^** D_2_ state is significantly lower in energy). Given that generation of *[**Acr^+^·**PhCl^•−^] is the first step required for Ph─Cl bond cleavage and subsequent functionalisation, this is fully consistent with the wavelength trends observed during reactivity studies, as well as TA observation of traces attributable to *[**Acr^+^·**PhCl^•−^].

### Mechanistic Discussion

#### Photoreactivity via Preassembly

As already discussed, reactivity and steady‐state fluorescence studies imply that **Acr^•^** possesses a high‐energy, long‐lived ES, whose solvent‐ and wavelength‐dependent formation correlates well with observed reactivity patterns. In isolation, these observations would suggest that **Acr^+^**‐based conPET catalysis is able to proceed simply via dynamic quenching of this emissive ES. However, this possibility can be excluded based on time‐resolved fluorescence and TA studies, neither of which indicates any significant interaction between *[**Acr^•^**]^EM^ and the model substrate PhCl. Instead, TA observations reveal that in cases where productive photoreactivity can be achieved, the photodynamic behaviour of **Acr^•^** is affected by the presence of substrate on tens on ps timescales, which is far faster than can be accounted for by purely dynamic quenching.

Most other mechanistic models that have been offered as alternatives to *[PC^•−^]‐based reactivity involve the formation of other, longer‐lived species derived from PC^•−^ that can engage in diffusion‐limited electron transfer in its stead (e.g., e^−^_(solv.)_, solv.^•−^, *[PC·H]^−^, etc.). However, the absence of other long‐lived transients in the TA analysis (besides unreactive *[**Acr^•^**]^EM^) suggests that such explanations are in this case unnecessary and insufficient. In contrast, all of our experimental observations so far (though vide infra) are fully accounted for if we assume the presence of preassembly between **Acr^•^** and substrate (an assumption that is also supported by DFT calculations). Additional reactivity studies also show that increasing substrate concentration leads to diminishing improvement in reaction performance once these concentrations become high enough, which would be qualitatively consistent with preassembly saturation (see Supporting Information, Section 3.3.1).

**Figure 7:**
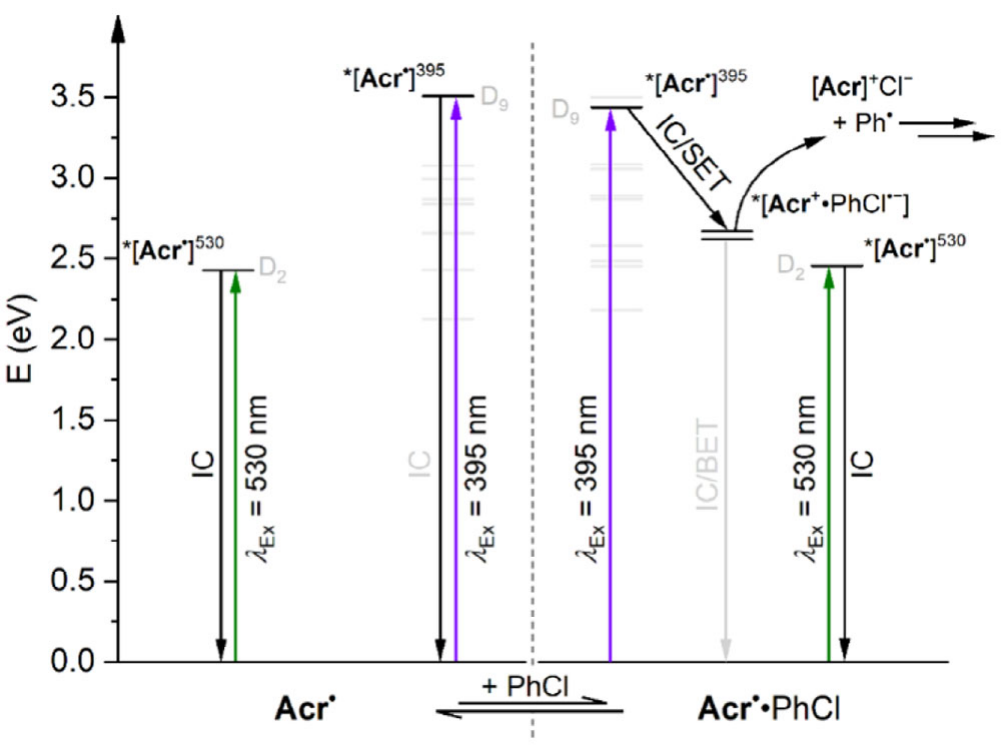
Energy level diagram for **Acr^•^** and the hypothesised **Acr^•^·**PhCl encounter complex in THF solvent, highlighting the key energy and electron transfer processes undergone following irradiation by either 530 or 395 nm light. Energy levels are from TD‐DFT. IC, internal conversion; BET, back‐electron transfer; SET, single‐electron transfer.

This model is summarised in Figure 7 for excitation of **Acr^•^** in the presence of PhCl in THF (where the model is simplified by the absence of *[**Acr^•^**]^EM^) and involves an equilibrium between “free” **Acr^•^** and the preassembled adduct **Acr^•^** ·PhCl (see Figure S71 for the analogous summary in MeCN). Photoexcitation of either free or preassembled **Acr^•^** at 395 nm leads to the initial, short‐lived ES *[**Acr^•^**]^395^ (tentatively D_9_). In the case of free **Acr^•^**, this then reverts rapidly and unproductively to the ground state via internal conversion. In contrast, for **Acr^•^** ·PhCl, the initial state can instead evolve into the intramolecular CT state *[**Acr^+^·**PhCl^•−^], whose lifetime is likely extended by spatial separation of the two charges (especially if cage escape occurs). This can then either revert unproductively to the ground state via back‐electron‐transfer (BET) or fragment to productively generate the key Ph^•^ intermediate, followed by downstream radical trapping steps. In contrast, excitation of **Acr^•^** at 530 nm leads to generation of *[**Acr^•^**]^530^ (tentatively D_2_), which is too low in energy to provide access to *[**Acr^+^·**PhCl^•−^] even when preassembled, so simply reverts to the ground state via rapid internal conversion.

This model accounts for all of the transients observed during TA experiments in THF and/or under 530 nm irradiation, and as it involves static quenching of the **Acr^•^** ES, it is also consistent with these traces being affected by the presence of substrates on tens of ps timescales. The observed wavelength‐dependence of the photoreactivity can also be understood based on the relative energies of the relevant ESs, which is supported by TD‐DFT calculations. The counterintuitive impact of excitation wavelength on many assumed conPET and e‐PRC reaction steps has been a topic of significant recent discussion, as have many apparent discrepancies between substrate reduction potentials and the potentials provided by the lowest energy (D_1_) excited states of the relevant PC^•−^.^[^ ^13^ ^]^ However, both phenomena are accounted for if photoreactivity proceeds not via the D_1_ state but rather a higher ES, as proposed here for **Acr^•^**. The possible involvement of such higher ESs is a notable emerging concept in modern synthetic photochemistry.^[^ ^13^ ^]^

#### Characterisation of *[**Acr^•^**]^EM^ and Evidence for Solvated Electrons Under 395 nm Irradiation in MeCN

Although TA experiments suggest that it is not directly relevant for photoreactivity, one facet of the **Acr^•^** system that still requires additional consideration, at least for reactions carried out using the synthetically relevant combination of MeCN solvent and short wavelength irradiation, is the unusual nature of the observed emissive state, *[**Acr^•^**]^EM^. As already noted, the existence of a long‐lived, emissive ES for **Acr^•^** is not necessarily surprising.^[^ ^50^, ^56^, ^57^ ^]^ However, in the context of other PC^•−^ states, it is certainly atypical. Similarly, although solvent‐dependent emission and emission from higher ESs are both well‐established, they are also very atypical.^[^ ^56^, ^66^ ^]^ As such, the apparent coincidence of all three phenomena in a single system is rather unexpected and difficult to rationalise (for additional discussion see Supporting Information, Section 6.1).

Fortunately, a plausible explanation is provided by more detailed consideration of the spectroscopic data related to *[**Acr^•^**]^EM^. In particular, close inspection of the TA trace attributed to *[**Acr^•^**]^EM^ reveals considerable similarity to that previously reported for the ES of the closed‐shell acridinium (i.e., *[**Acr** ^+^]).^[^ ^35^ ^]^ Comparable similarities between *[**Acr^•^**]^EM^ and *[**Acr** ^+^] can also be seen in both their emission (vide supra) and excitation spectra (see Supporting Information, Section 5.1).^[^ ^67^ ^]^ At first glance, this might appear to suggest a rather mundane explanation for the observed emission: contamination of **Acr^•^** by **Acr** ^+^ (cf. previous mistaken attributions of emission to in situ generated PC^•−^).^[^ ^53^, ^54^, ^55^ ^]^ However, this explanation can be excluded for several reasons. First, UV–vis and other spectroscopic measurements confirm the absence of any appreciable **Acr** ^+^ in samples of isolated **Acr^•^**, indicating that it could not be present in more than trace amounts. Second, although it is known that fluorescence measurements can be extremely sensitive to the presence of trace impurities, the same is not true of TA methods. The large responses observed in these experiments thus cannot plausibly be attributed to the presence of trace **Acr** ^+^. Third, and perhaps most conclusively, the presence of an **Acr** ^+^ impurity as the active emitter would not be consistent with the clear solvent dependence of the observed emission as **Acr** ^+^ emission would not be limited to MeCN solutions.

These factors suggest that in situ observation of *[**Acr**]^+^ must instead arise via initial photoexcitation of **Acr^•^**; in other words, that absorption of sufficiently high energy photons induces photoionisation of **Acr^•^** to **Acr** ^+^. As such, rather than a conventional, fully localised ES, it appears that the emissive state *[**Acr^•^**]^EM^ is instead best described as an ion pair *[**Acr** ^+^ **·**e^−^], which can also be considered a “charge‐transfer‐to‐solvent” state consisting of an acridinium cation and an ejected, MeCN‐solvated electron, e^−^_(MeCN)_.^[^ ^68^, ^69^, ^70^ ^]^ Notably, such a characterisation is fully consistent with the observed wavelength dependence of emission (as only photons of sufficiency energy will be able to reach the e^−^ ejection threshold) and also provides a simple explanation for the observed solvent dependence (as the ability of MeCN to accept an electron is much higher than that of THF and other less polar/reducible solvents). The absence of a localised, emissive *[**Acr^•^**]^EM^ state is also consistent with TD‐DFT calculations, which predict that such states would have lifetimes far lower than the ca. 10 ns observed experimentally and would relax preferentially via nonradiative pathways (see Supporting Information, Section 8.2). In this scenario, the observed emission from *[**Acr** ^+^] could occur either by secondary excitation of **Acr** ^+^ after the initial photoionisation step or by photoionisation generating excited *[**Acr** ^+^] directly (for additional discussion of these two options, see Supporting Information, Section 8.1.2).

In either case, the *[**Acr** ^+^] ES itself is not directly involved in the observed photoreactivity (vide supra), consistent with the oxidising rather than reducing nature of *[**Acr** ^+^] (cf. control experiments using **Acr** ^+^, Supporting Information, Section 4). However, the same cannot be assumed of the concomitantly generated (and in these experiments spectroscopically invisible)^[^ ^71^ ^]^ solvated electron, e^−^_(MeCN)_. In fact, e^−^_(MeCN)_ are powerful reductants and have previously been proposed as key intermediates for conPET‐type reactions that use TADF chromophores such as 4CzIPN and 4DPAIPN.^[^ ^19^, ^20^ ^]^ Moreover, the lifetime of the e^−^_(MeCN)_ generated from **Acr^•^** must be at least as long as the 10 ns *[**Acr** ^+^] lifetime (assuming that in the absence of substrate e^−^_(MeCN)_ decays primarily by recombination with *[**Acr** ^+^] and/or **Acr** ^+^), which is comfortably long enough for diffusion‐limited reactivity.

**Scheme 2:**
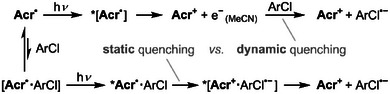
Parallel pathways proposed for photoreduction of aryl chlorides (ArCl) by **Acr^•^** in MeCN under 395 nm irradiation: via either solvated electrons (dynamic quenching, top) or preassembly (static quenching, bottom).

As such, in addition to the preassembly‐mediated static quenching pathway noted above, a second, dynamic quenching pathway via e^−^_(MeCN)_ is also likely to be available for reactions in MeCN under short wavelength irradiation (Scheme 2). Indeed, based on the initial photoreactivity studies, it is likely that this is in fact the major pathway under the most synthetically relevant conditions (i.e., 395 nm irradiation in MeCN), with static quenching providing only a more minor contribution.^[^ ^72^ ^]^ In contrast, reactions under conditions where e^−^_(MeCN)_ formation is not available (e.g., under 530 nm irradiation and/or in THF) must rely on the preassembly contribution only, which provides a convenient explanation for the greatly diminished—but still clearly nonzero—conversions achieved in these cases (though other factors such as reduced photostability in THF and more efficient preassembly in MeCN^[^ ^73^ ^]^ may also contribute for certain reactions).

## Conclusion

We have successfully isolated the neutral radical **Acr^•^**, which is the one‐electron reduced form of a ubiquitous acridinium PC, and characterised it both structurally and electronically. Using this authentic material, we have been able to perform a suite of studies into the photoreactivity of this species, which plays a key role in the high‐profile but much‐debated proposed mechanisms of conPET catalysis. In the course of our investigations, we discovered that a long‐lived, emissive excited state that had previously appeared to be the key reactive intermediate was in fact not quenched by the substrate and not directly relevant to the mechanism. Instead, we were able to observe quenching of much shorter‐lived excited states on ultrafast timescales (tens of ps), significantly shorter than would be expected for diffusion‐limited processes (ca. 1 ns). This strongly points towards preassembly between the substrate and **Acr^•^** prior to excitation, which is supported by (TD‐)DFT calculations. Nevertheless, detailed analysis of the emissive state also provides compelling evidence for a simultaneous, diffusion‐limited pathway based on solvated electrons, which can operate specifically under the most synthetically relevant conditions and is likely the dominant productive mechanism in these particular cases. The feasibility of multiple pathways has significant implications for the future scope and applications of conPET‐type reactivity (especially for PC based on the acridinium scaffold) and implies that improved preassembly could be an effective design strategy for improved catalysis, especially in less reducible solvents (e.g., by optimising PC^•−^/substrate π–π interactions), but also that the absence of preassembly need not act as a strict limit on substrate scope (e.g., for reducible substrates other than aromatic halides that lack π systems).

These results also provide strong evidence for the fundamental feasibility of the conPET mechanism, despite previous arguments to the contrary, at least in the case of **Acr^+^**/**Acr^•^** photocatalysis. Given the increasingly evident mechanistic complexity of conPET reactions (and of PRC in general), as well as the wide variety of transformations and PC structures that have been investigated, it seems unlikely that there is a single, “one size fits all” explanation that can account for every example of proposed *[PC^•−^] reactivity. Indeed, these results clearly challenge the typical assumption that a single mechanistic pathway will be operative even in individual cases. Nevertheless, it is very likely that the conclusions drawn here will also be applicable to other types of PC/PC^•−^ that have been explored for conPET/e‐PRC applications. For example, in our previous work studying isolated DCA^•−^ and NpMI^•−^, we were able to show broadly similar patterns of reactivity to those described herein for **Acr^•^**, but were unable to reconcile our general conclusion (that results were most easily explained by the initially proposed conPET/e‐PRC mechanisms) with observations of unexpected wavelength‐dependence or with previous reports of short PC^•−^ lifetimes.^[^ ^41^ ^]^ However, if DCA^•−^ and NpMI^•−^ behave similarly to **Acr^•^**, these discrepancies are fully accounted for.

Finally, we will note that previous discussions of substrate/catalyst preassembly in the PRC literature have generally focused on cases where the photocatalyst does not possess an apparent long‐lived excited state, with the common implicit assumption that such preassembly is unnecessary in cases where a long‐lived excited state does exist.^[^ ^74^ ^]^ Our results demonstrate that this approach is, in principle, insufficient, and that preassembly can in fact play an appreciable role even in cases where a long‐lived excited state is apparently observed. In particular, preassembly may provide access to higher ESs that are short‐lived but much more reactive than the lowest‐energy ES. The potential synthetic utility of higher‐ES photoreactivity is enormous, but is only just beginning to be explored and understood.^[^ ^13^ ^]^

## Supporting Information

Crystallographic Information is available from the CCDC.^[^ ^75^ ^]^ The authors have cited additional references within the Supporting Information.^[^ ^76^, ^77^, ^78^, ^79^, ^80^, ^81^, ^82^, ^83^, ^84^, ^85^, ^86^, ^87^, ^88^, ^89^, ^90^, ^91^ ^]^

## Author Contributions

S.J.H., K.M.M.S., P.P.F. and J.M.W. performed the experimental and theoretical investigations. J.M.W. assisted with data analysis. D.J.S. and I.P. provided supervision and resources. S.J.H. wrote the original draft manuscript, and all authors contributed to review and editing. D.J.S. conceptualised the study.

## Conflict of Interests

The authors declare no conflict of interest.

## Supporting information



Supporting Information S1

Supporting Information S2

## Data Availability

The data that support the findings of this study are available in the Supporting Information of this article.
