# Supplementary material for: Counterintuitive Photochemistry of an Isolated Acridinyl Radical: ConPET via Preassembly, Solvated Electrons or a Long‐Lived Excited State?
Source: Angew Chem Int Ed Engl. 2025 Jul 2;64(34):e202506701. doi: 10.1002/anie.202506701 (PMC12363617; doi:10.1002/anie.202506701)
Supplement: Supplementary file 1 — Supporting Information S1 [file ANIE-64-e202506701-s001.pdf]

Supporting Information

Counterintuitive Photochemistry of an Isolated Acridinyl Radical: ConPET via Preassembly, Solvated Electrons, or a Long-Lived Excited State?

Samuel J. Horsewill,[a] Katherine M. M. Sharrock,[b] Péter P. Fehér,[c] Jack M. Woolley,[d] Imre Pápai,*[c] and Daniel J. Scott*[a]

[a] Department of Chemistry, University of Bath, Claverton Down, Bath, BA2 7AY, United Kingdom

[b] Department of Chemistry, University of Warwick, Coventry, CV4 7AL, United Kingdom

[c] Institute of Organic Chemistry, HUN-REN Research Centre for Natural Sciences, Magyar Tudósok Körútja 2, H-1117 Budapest, Hungary

[d] Department of Physics, University of Warwick, Coventry, CV4 7AL, United Kingdom

*E-mail: papai.imre@ttk.hu, ds2630@bath.ac.uk

Contents

1 Methods 2
 1.1 General experimental procedures 2
 1.2 LED apparatus 3
2 Synthesis and characterisation of Acr$^{•}$ 5
 2.1 Preparation 5
 2.2 NMR spectroscopy 5
 2.3 EPR spectroscopy 6
 2.4 UV-vis spectroscopy 8
3 Acr$^{•}$ (photo)reactivity 11
 3.1 Photostability 11
 3.2 THF decomposition mechanistic analysis 16
  3.2.1 Calculated H-atom transfer to Acr$^{•}$ 16
  3.2.2 THF radical trapping experiments 16
 3.3 Photoreactivity with aryl chlorides 19
  3.3.1 Study of concentration effects on reactivity 25
 3.4 Super-stoichiometric reaction yields 29
4 $[Acr]^{+}[BF_4]^{-}$ reactivity 30
 4.1 NMR spectrum of $[Acr]^{+}[BF_4]^{-}$ 30
 4.2 UV-vis spectra of $[Acr]^{+}[BF_4]^{-}$ 31
 4.3 Photostability 32
 4.4 Photoreactivity with aryl chlorides 37
5 Fluorescence and transient absorption spectroscopy 39
 5.1 Fluorescence spectroscopy 39
 5.2 Fluorescence lifetime analysis 40
 5.3 Transient absorption spectroscopy 41
6 Additional discussion 51
 6.1 Solvent-dependent Acr$^{•}$ fluorescence 51
 6.2 Photoreduction of solvent 52
 6.3 Estimation of collision rate and quenching efficiency 53
7 Crystallographic information 54
 7.1 General crystallographic information 54
 7.2 XRD data for Acr$^{•}$ 54
8 Computational Studies 58
 8.1 TD-DFT details 58
  8.1.1 Preassembly structures 64
  8.1.2 Origin of emission from *[Acr^{+}] 65
 8.2 TD-DFT information tables 67
9 References 80

1 Methods

1.1 General experimental procedures

Unless stated otherwise, all reactions, manipulations and spectroscopic acquisitions were performed under an N_2 or Ar atmosphere (< 0.1 ppm O_2, H_2O) through use of an MBraun MB20G glovebox and standard Schlenk line techniques. All glassware was oven dried (>160 °C) overnight prior to use.

Hexane, pentane and toluene were collected from an Innovative Technologies inc. PS-400-7 solvent purification system before being degassed and stored over molecular sieves (3 Å). THF and CH_3CN were pre-dried over activated molecular sieves (3 Å), then dried in a still (THF, sodium/benzophenone; MeCN, CaH_2) before being degassed and stored over molecular sieves (3 Å). We note that trace benzene is observed in some NMR spectra recorded in H_8-THF, which is due to minor decomposition of benzophenone during the drying process. NMR solvents were dried by refluxing over a drying agent (C_6D_6, potassium; CD_3CN, CaH_2) in a sealed ampoule under reduced pressure, then degassed and distilled before being stored over molecular sieves (3 Å). KC_8 and a 5% dispersion of Na on NaCl (5% Na/NaCl) were prepared according to literature procedures.[1,2] Unless otherwise stated, [**Acr**]$^+$[BF_4]$^-$ and all other starting materials were purchased from major suppliers. Unless supplied under inert atmosphere or noted otherwise, solids were dried under vacuum and liquids were degassed and dried over molecular sieves (3 Å).

NMR spectra were recorded on a Bruker Avance (400 MHz), a Bruker Neo (400 MHz), or an Agilent ProPulse (500 MHz) spectrometer. Chemical shifts, *δ*, are reported in parts per million (ppm); ^1H NMR shifts were referenced externally to $SiMe_4$ and referenced internally to residual solvent peaks, while ^{31}P NMR shifts were referenced externally to 85% H_3PO_4 (aq.). The abbreviations s, d, t, q, m are used to indicate singlets, doublets, triplets, quartets and multiplets, respectively. Where integrals are given for $^{31}P\{^1H\}$ NMR spectra, the spectra were recorded using an inverse-gated decoupling experiment which was confirmed to provide quantitative integration by measurement of a sample containing known concentrations of Ph_3PO standard and $PhPO(OMe)_2$ (each at 5 mM), which was comparable to the concentrations used in the experiment. Samples for NMR were prepared inside an oxygen-free glovebox using stock solutions in anhydrous solvents (stored at −35 °C) and were then removed in an air-tight J Youngs tap NMR tube for irradiation and analysis.

EPR spectra were recorded at room temperature on a Bruker EMXmicro X-band continuous wave EPR spectrometer at the CAESR facility at the University of Oxford. Samples for EPR were prepared inside an oxygen-free glovebox and removed in an air-tight J Youngs tap EPR tube for analysis.

UV-vis spectra were recorded at room temperature using an Ocean Optics assembly with an Ocean Optics DH2000 light source and Ocean Insight Flame miniature spectrometer, using fibre-optic cables to pass light through a sample in the glovebox. Steady-state fluorescence measurements were recorded at room temperature using an Agilent Cary Eclipse spectrophotometer, using an air-tight 1×1 cm quartz fluorescence cuvette. Fluorescence lifetime data were recorded on a Horiba Fluorolog-3 with 402 nm NanoLed attachment for the excitation source using an airtight 1 mm path length quartz cuvette mounted at *ca.* 30° to the incident light. Fluorescence lifetimes were obtained using a dual exponential decay fitting program in Origin. Where electronic spectroscopy was carried out outside of the glovebox, samples were prepared inside an oxygen-free glovebox and removed in air-tight cuvettes (J Young's tap or foiled screwcap) and stability over the timescale of the experiment was confirmed using UV-vis spectroscopy (i.e. no changes in absorbance were observed).

Ultrafast transient absorption measurements were performed at the Warwick centre for ultrafast Spectroscopy (WCUS, www.warwick.ac.uk/research/rtp/wcus). A detailed explanation of the setup is available elsewhere,[6,7] with only key details pertaining to the experiments detailed here. Samples were placed in the spectrometer using an airtight 1 mm path length quartz cuvette. The sample was translated in the plane of the probe focus to ensure fresh sample was interrogated for each pump probe delay. Samples were photoexcited at both 395 and 530 nm generated by using an Optical parametric amplifier (TOPAS-Prime with UV extension, Light Conversion). Changes in the Optical density (ΔOD) were collected through the use of a fibre coupled spectrometer (Avantes, AvaSpec-ULS1650F). GloTarAn (Global Target Analysis) software was used for background correction and to generate EADS spectra and obtain lifetime fits.[8]

Elemental analysis was performed as an air-sensitive sample by Orla McCullough at London Metropolitan University, with each measurement performed in duplicate with a combustible. We attribute the low %C value measured for **Acr•** to incomplete combustion with the effect exacerbated by the exceptionally high wt% of C.

1.2 LED apparatus

The LED apparatus used in this study is one that we have recently described elsewhere.[3,4] For clarity and ease of reference, pertinent information is reproduced here.

Individual LEDs are mounted to a heatsink plate, with the six-LED array powered by a 28 W power supply. The sample block was kept cool by circulation of cooling water, and JY NMR tubes were positioned above the LEDs in a bath of deionised water to assist with thermal transfer. The LEDs used for this study are listed below:

Luminus SST-10-UV-A130, UV (395 nm)

Osram OSLON SSL 120, Deep Blue (455 nm)

Osram OSLON SSL 120, Green (530 nm)

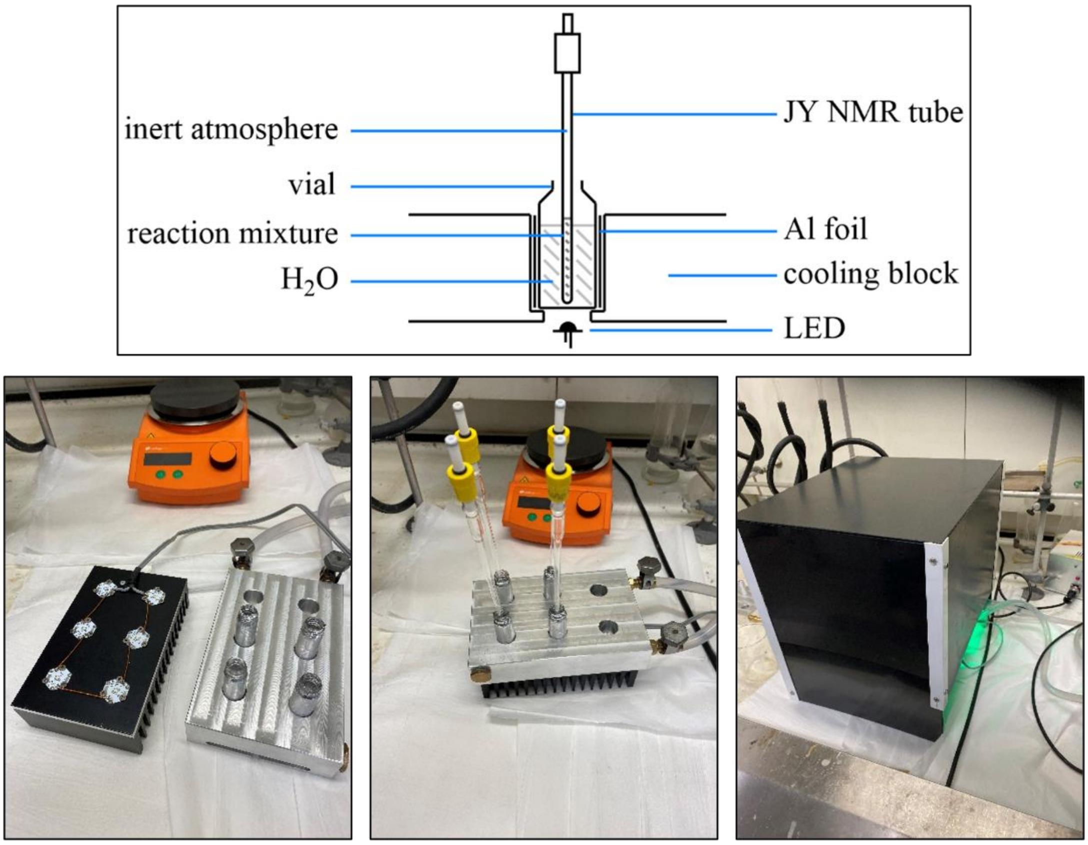

Figure S1: Illustration and photographs of the apparatus used for photochemical experiments. Similar apparatus has been described previously by the Wolf group.[5] Schematic illustration not to scale. Sockets in cooling block have diameter 1.8 cm to fit vials. Base of vial is ca. 9 mm above the LEDs.

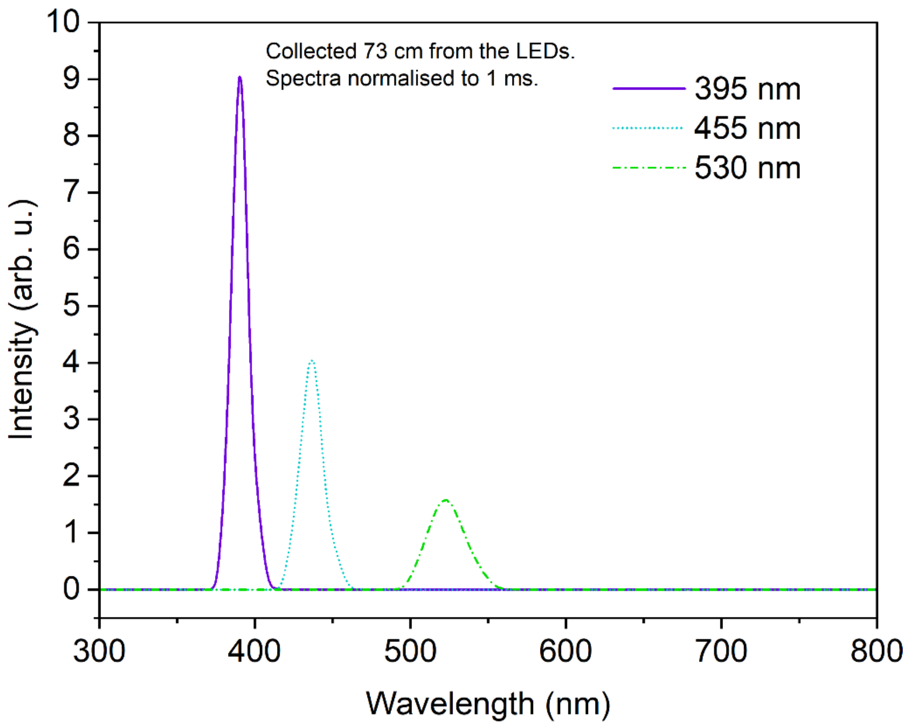

Figure S2: Emission spectra of the LEDs used in this study. Spectra were recorded at a fixed distance from the fibre-optic input of an Ocean Insight Flame spectrometer and were normalised to account for different integration times (395 nm, 0.5 ms; 455 and 530 nm, 1 ms). The abbreviation "arb. u." is used to refer to the arbitrary units of the emission measurements.

2 Synthesis and characterisation of Acr•

2.1 Preparation

A Schlenk flask was charged with [**Acr**]$^+$[BF_4]$^-$ (225 mg, 0.392 mmol) and 5% Na/NaCl (196 mg, 0.426 mmol Na), then THF (10 mL) was added with stirring. The mixture was stirred at room temperature for 2 h, during which time a colour change from yellow to deep red was observed. THF was removed *in vacuo* and toluene (15 mL) was added to dissolve **Acr•**. The suspension was then filtered, toluene was removed *in vacuo* from the filtrate, and the resulting dark solids were dried *in vacuo* overnight to provide analytically pure **Acr•** as a red solid (175.6 mg, 0.361 mmol, 92% yield). Further purification could be carried out by either sublimation at 200 °C under static vacuum or (preferentially) by crystallisation from a saturated pentane solution cooled to −35 °C.

A similar procedure could be carried out using KC_8 as the reducing agent. However, this resulted in a lower yield (57%), which we believe is due to less efficient extraction of **Acr•** into toluene in the presence of graphitic carbon. Sublimation was not possible directly from graphitic residues.

UV-vis (MeCN): λ_{max} (ε) = 361 (10 320), 484 (3330), 516 (4160), 635 nm (510 M^{-1} cm^{-1}).

UV-vis (THF): λ_{max} (ε) = 363 (12 100), 484 (4530), 518 (5380), 629 nm (730 M^{-1} cm^{-1}).

Elemental Analysis: Anal. Calcd. for **Acr•**: C, 88.84%; H, 8.28%; N, 2.88%. Found (crystalline): C, 86.64-87.02%; H, 8.23-8.50%; N, 2.71-2.81%.

2.2 NMR spectroscopy

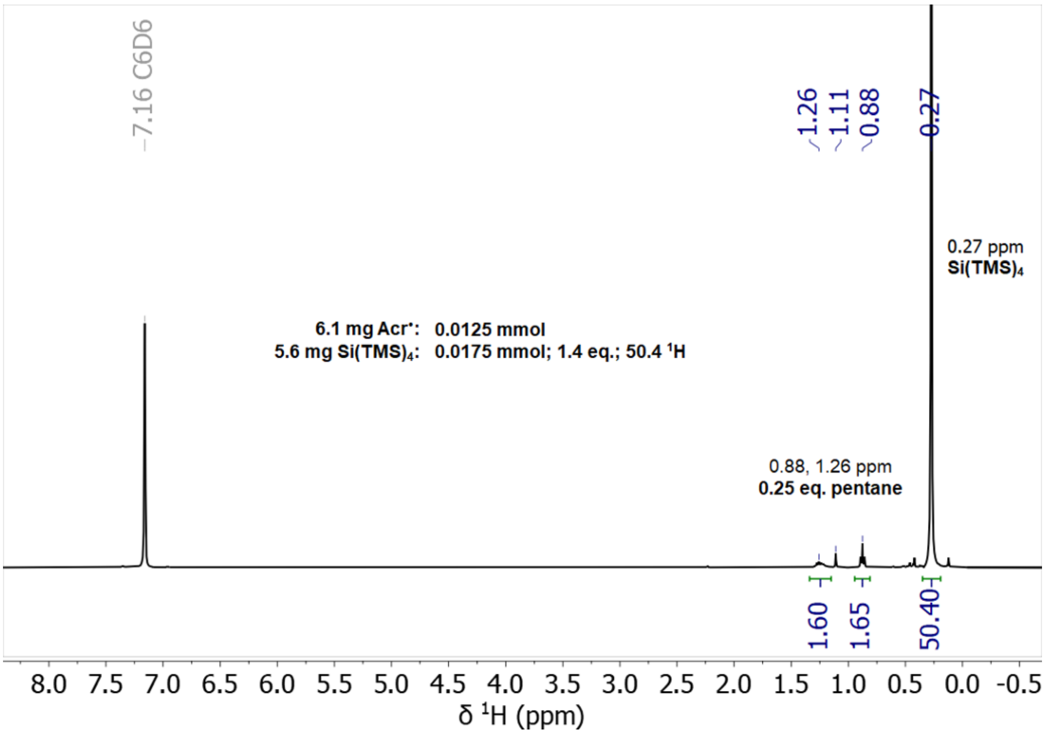

Figure S3: ^{1}H NMR spectrum of crystallised **Acr•** as recorded in C_6D_6. Si(TMS)$_4$ included as internal standard. Also observed is 0.25 eq. of residual, co-crystallised pentane, which is only partially removed during post-crystallisation workup. Assignments: δ = 0.27 ppm, Si(TMS)$_4$; δ = 0.88, 1.26 ppm, pentane.

2.3 EPR spectroscopy

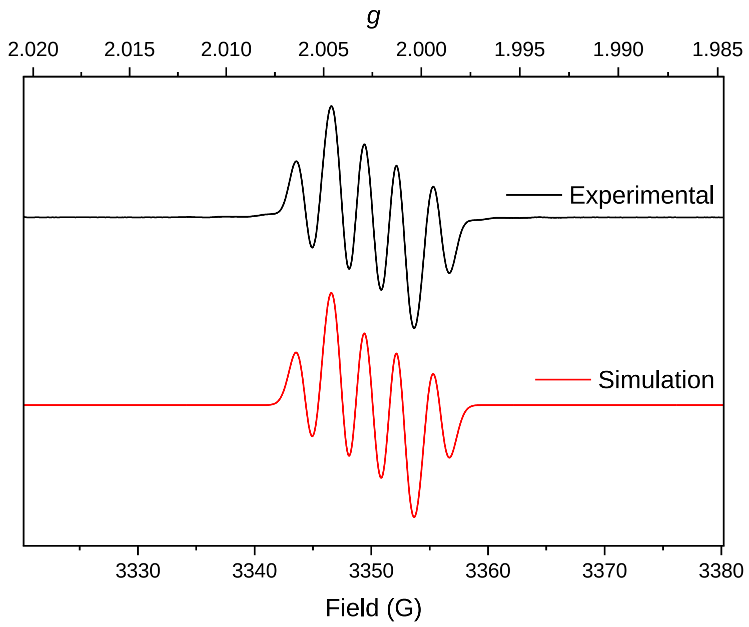

Figure S4: X-band EPR spectrum of **Acr•**, recorded on a 200 μM toluene solution at 298 K. Simulated as: g = 2.00252; 2× A(^{1}H) = 9.294 MHz, 1× A(^{14}N) = 6.851 MHz. Experimental parameters: frequency, 9.3897 GHz; power, 0.07962 mW; Mod. Amp., 0.200 G.

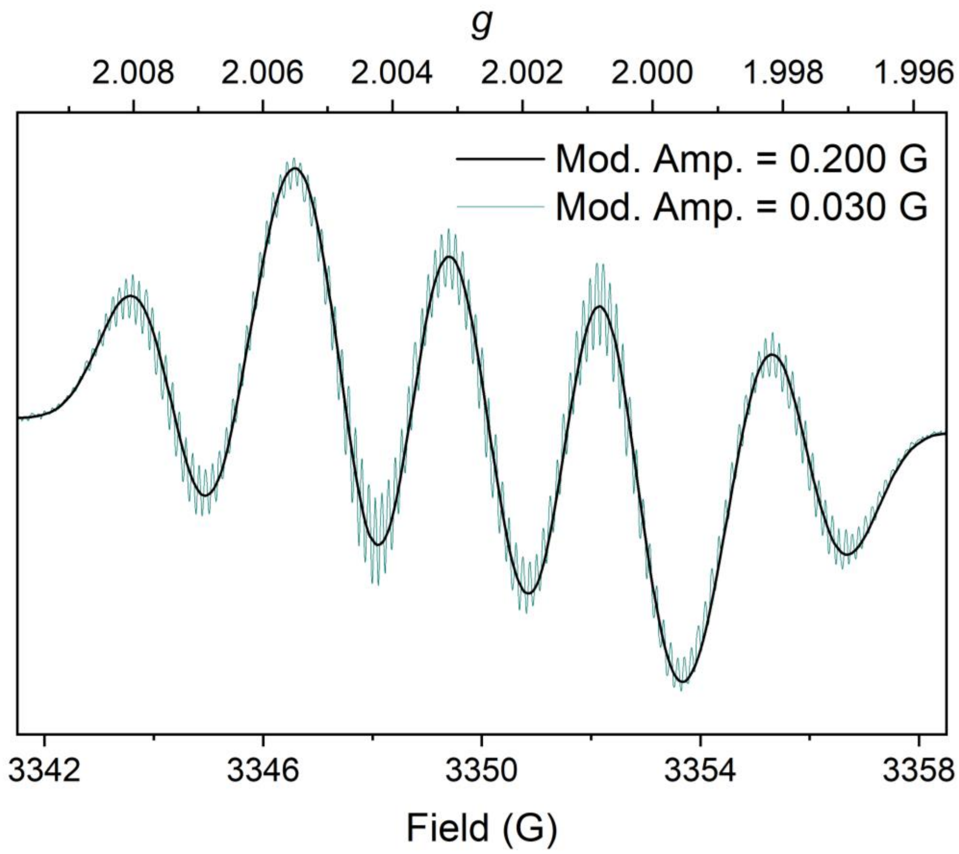

Figure S5: X-band EPR spectra of **Acr•**, recorded on a 200 μM toluene solution at 298 K. The black line shows the spectrum recorded with a modulation amplitude of 0.200 G (time constant = 327.68 ms), while the teal line shows the spectrum recorded with a modulation amplitude of 0.03 G (time constant = 81.92 ms). All other experimental parameters were identical: frequency, 9.3897 GHz, power, 0.07962 mW, time = 600 s.

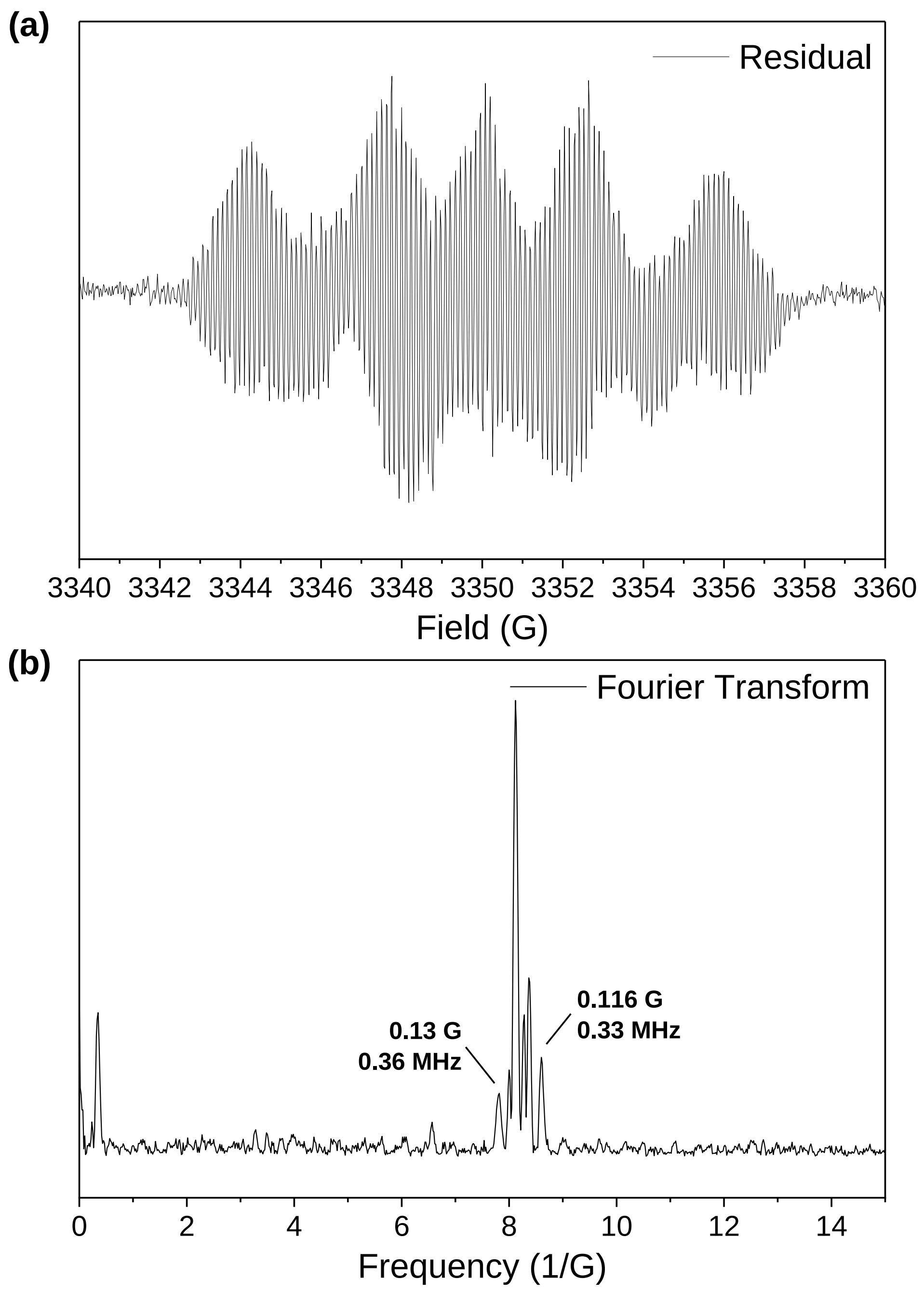

Figure S6: (a) Residual after subtraction of normalised 0.200 G modulated X-band EPR spectrum from 0.030 G modulated spectrum. (b) Fourier transform of the residual, showing the range of small hyperfine coupling frequencies present.

2.4 UV-vis spectroscopy

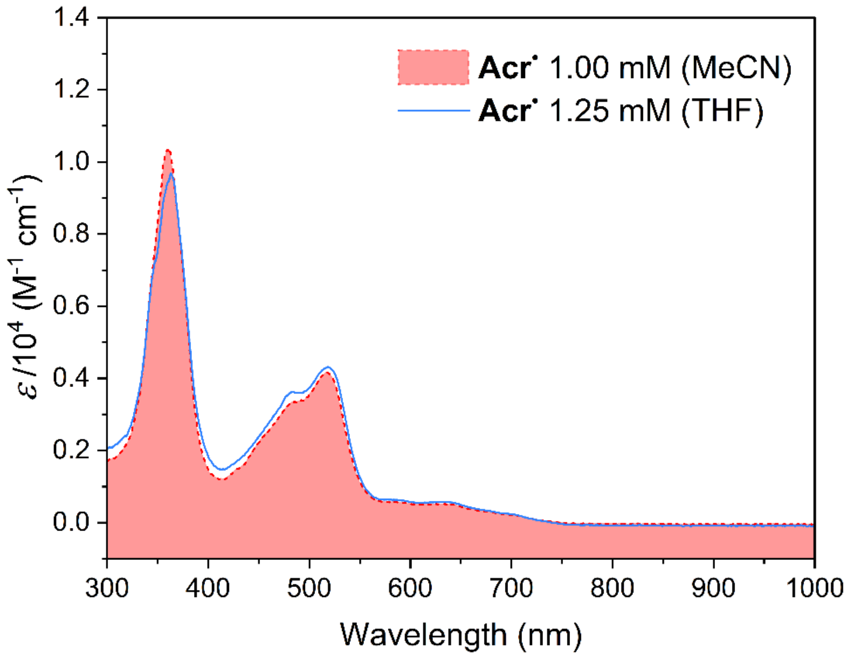

Figure S7: UV-visible absorption spectra of **Acr•** in MeCN and in THF solutions.

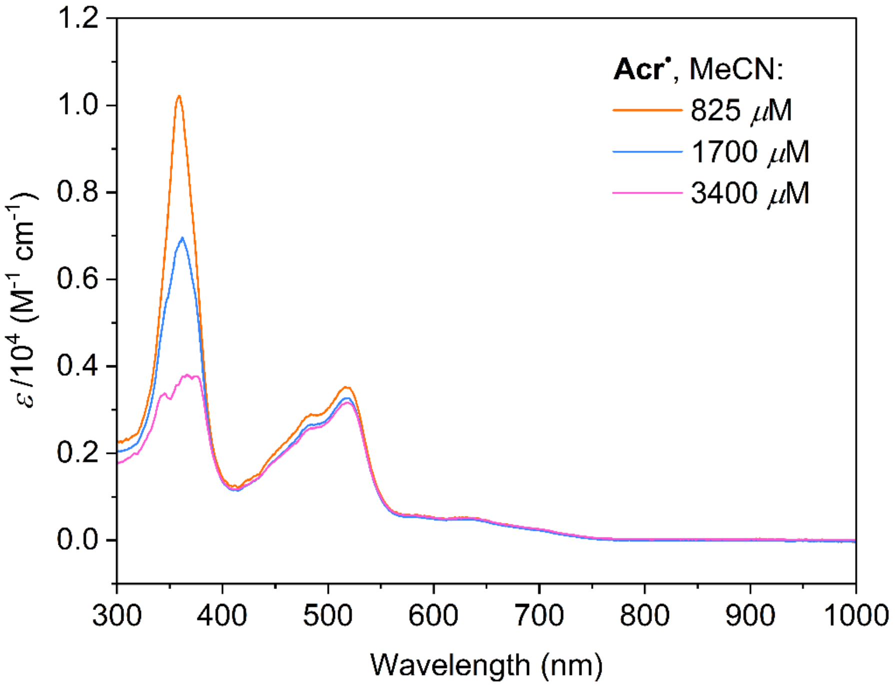

Figure S8: UV-vis absorption spectra of **Acr•** in MeCN solution at various concentrations. The reduction in intensity of the peak at 350 nm at higher concentrations is due to saturation of the detector.

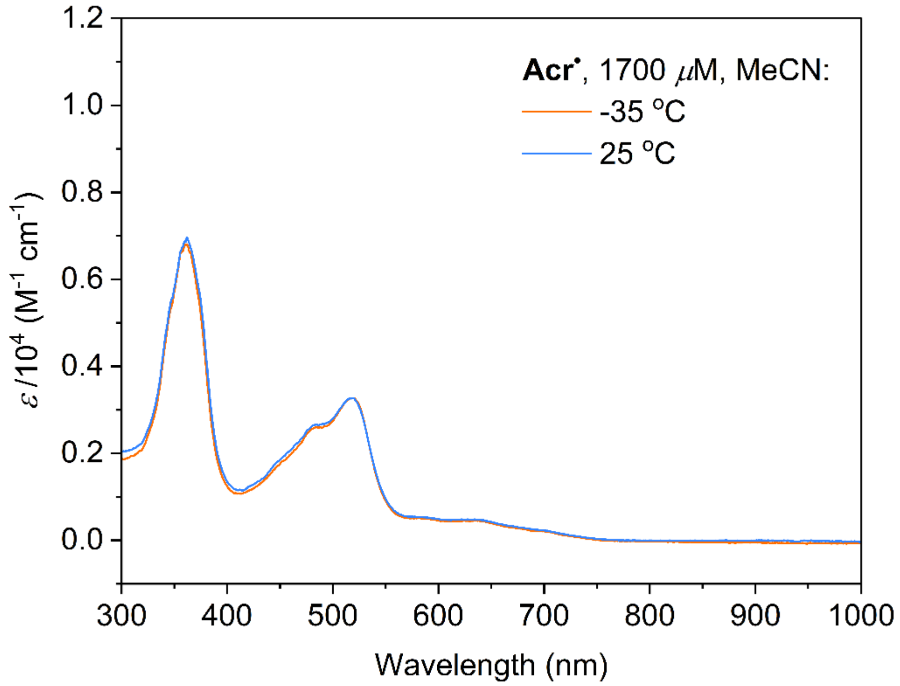

Figure S9: UV-vis absorption spectra of **Acr•** (1700 μM) in MeCN solution at different temperatures.

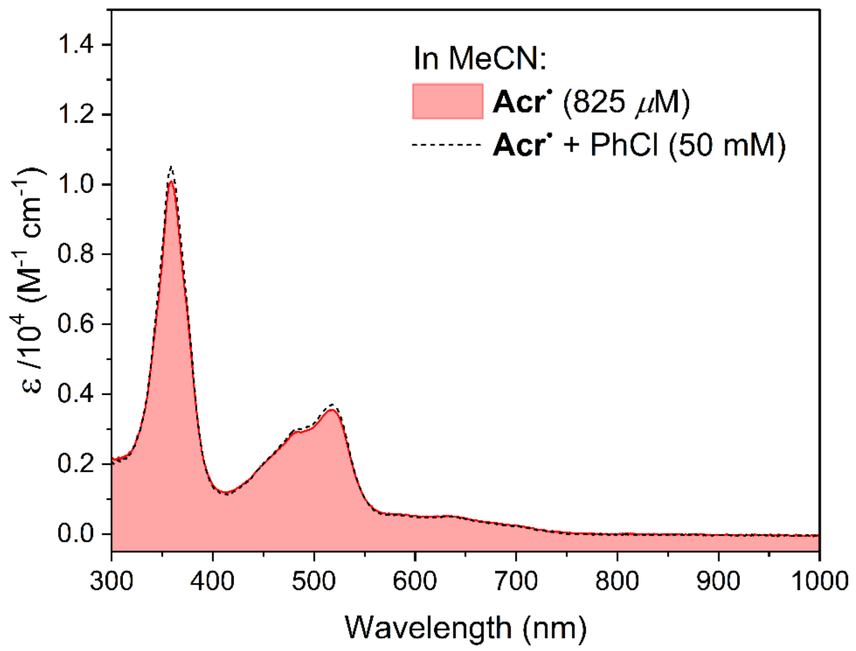

Figure S10: UV-vis absorption spectrum of **Acr•** in MeCN solution, and in MeCN solution with chlorobenzene substrate present.

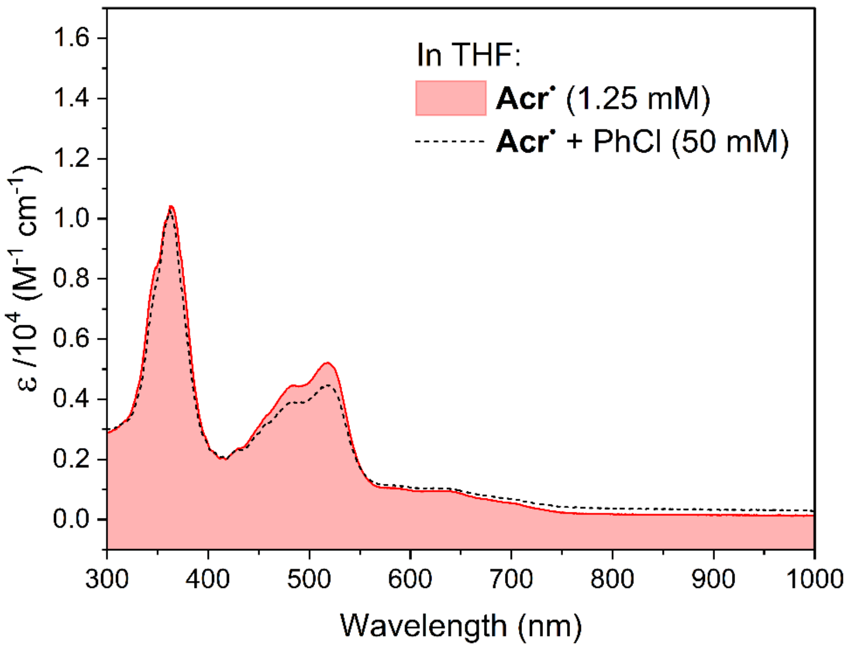

Figure S11: UV-vis absorption spectrum of **Acr•** in THF solution, and in THF solution with chlorobenzene substrate present.

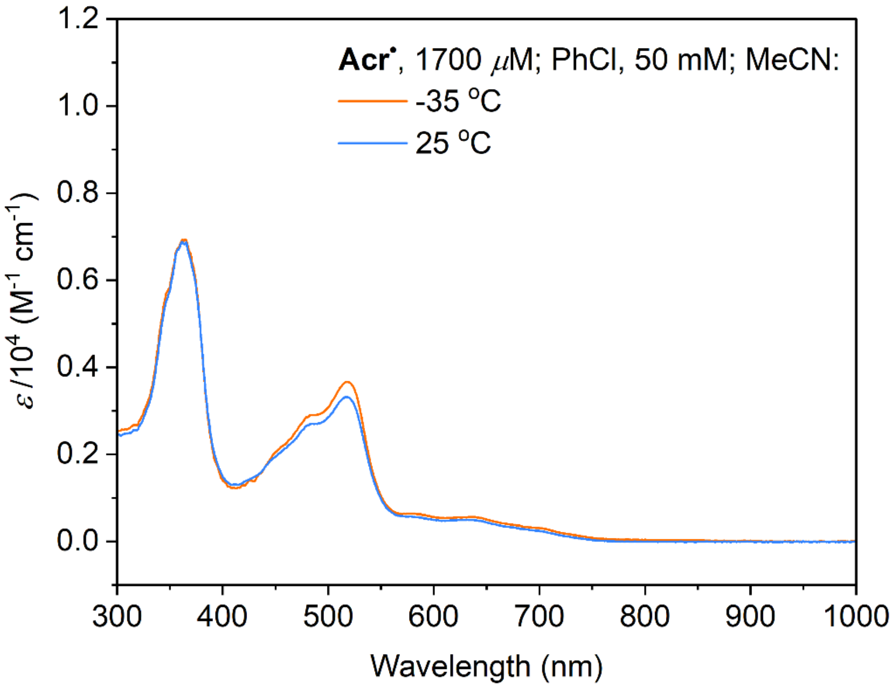

Figure S12: UV-vis absorption spectra of **Acr•** (1700 μM) in MeCN solution in the presence of PhCl (50 mM) at different temperatures.

3 Acr$^{\bullet}$ (photo)reactivity

3.1 Photostability

In MeCN or THF, **Acr$^{\bullet}$** (MeCN, 1.7 mM; THF, 5 mM) was irradiated by 395, 455 or 530 nm light, with or without $P(OMe)_3$ (50 mM), for 16 h. NMR spectra were recorded, then the solutions diluted (MeCN, 0.425 mM; THF, 1.25 mM) to record UV-vis spectra. In MeCN, no appreciable new resonances in the NMR spectra were detected and no large changes in the UV-vis spectra were observed (with the only minor changes detected occurring after 395 nm irradiation), despite the long irradiation time, indicating that **Acr$^{\bullet}$** does not undergo significant photodegradation in MeCN at any of these three wavelengths. In contrast, in THF significant changes in both the ^{1}H NMR and UV-vis spectra were observed under 395 nm irradiation, indicating that **Acr$^{\bullet}$** is not photostable under these conditions. Note that the new spectra obtained in this case are not consistent with simple oxidation of **Acr$^{\bullet}$** to **Acr^{+}**; see Figure S38 and Figure S39 for the NMR and UV-vis spectra of **Acr^{+}**, respectively. When photostability tests were undertaken in 2-methyl-THF solvent rather than THF, under otherwise identical conditions, **Acr$^{\bullet}$** experienced less photodecomposition, clearly visible from the UV-vis spectra (Figure S20). This supports the notion that the decomposition involves ring-opening of THF.

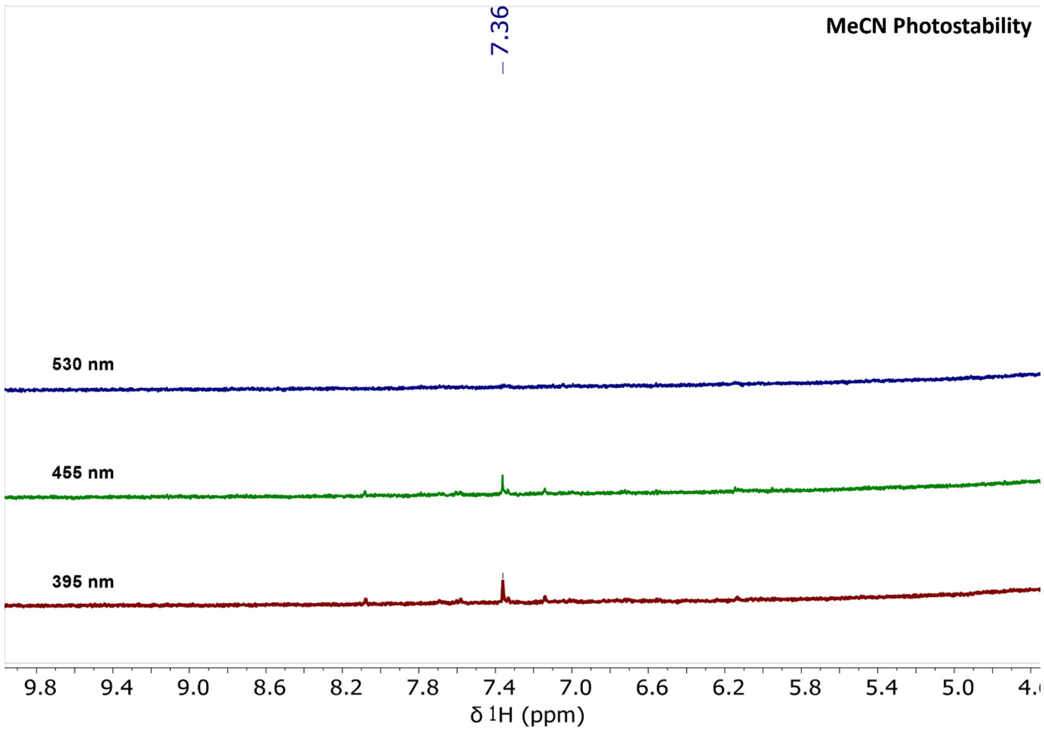

Figure S13: ^{1}H NMR spectra of **Acr$^{\bullet}$** in MeCN following irradiation by 395, 455 or 530 nm light for 16 h. Assignment: δ = 7.36 ppm, benzene.

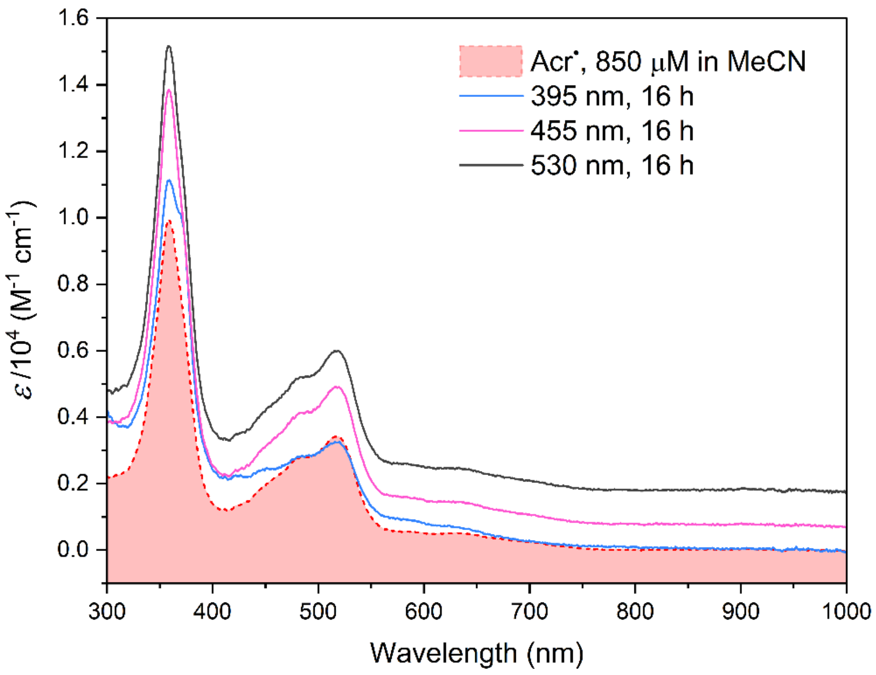

Figure S14: UV-vis spectra of Acr• in MeCN following irradiation by 395, 455 or 530 nm light for 16 h. 455 nm and 530 nm spectra are vertically offset by 1000 and 2000 M^{-1} cm^{-1} respectively for clarity.

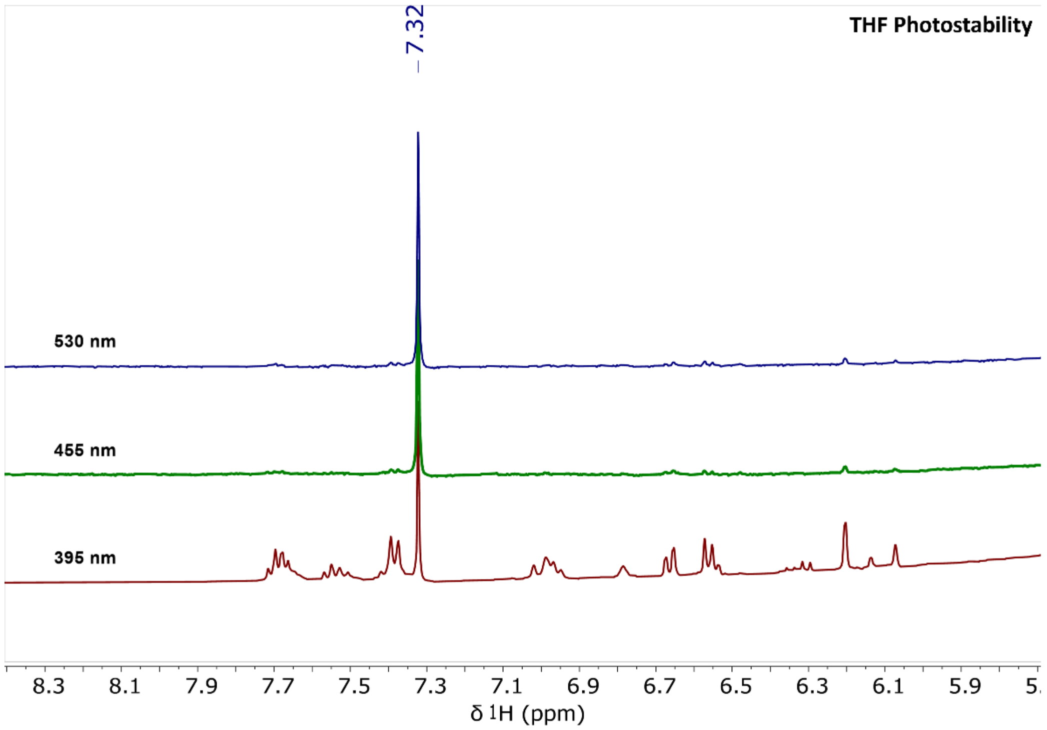

Figure S15: ^{1}H NMR spectra of **Acr•** in THF following irradiation by 395, 455 or 530 nm light for 16 h. Assignment: δ = 7.32 ppm, benzene.

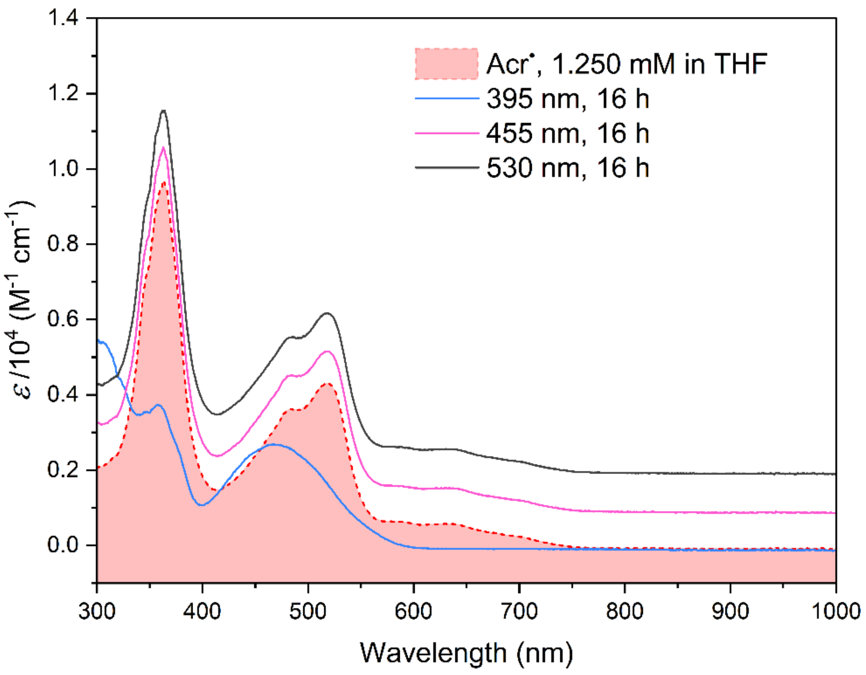

Figure S16: UV-vis spectra of **Acr•** in THF following irradiation by 395, 455 or 530 nm light for 16 h. 455 nm and 530 nm spectra are vertically offset by 1000 and 2000 M^{-1} cm^{-1} respectively for clarity.

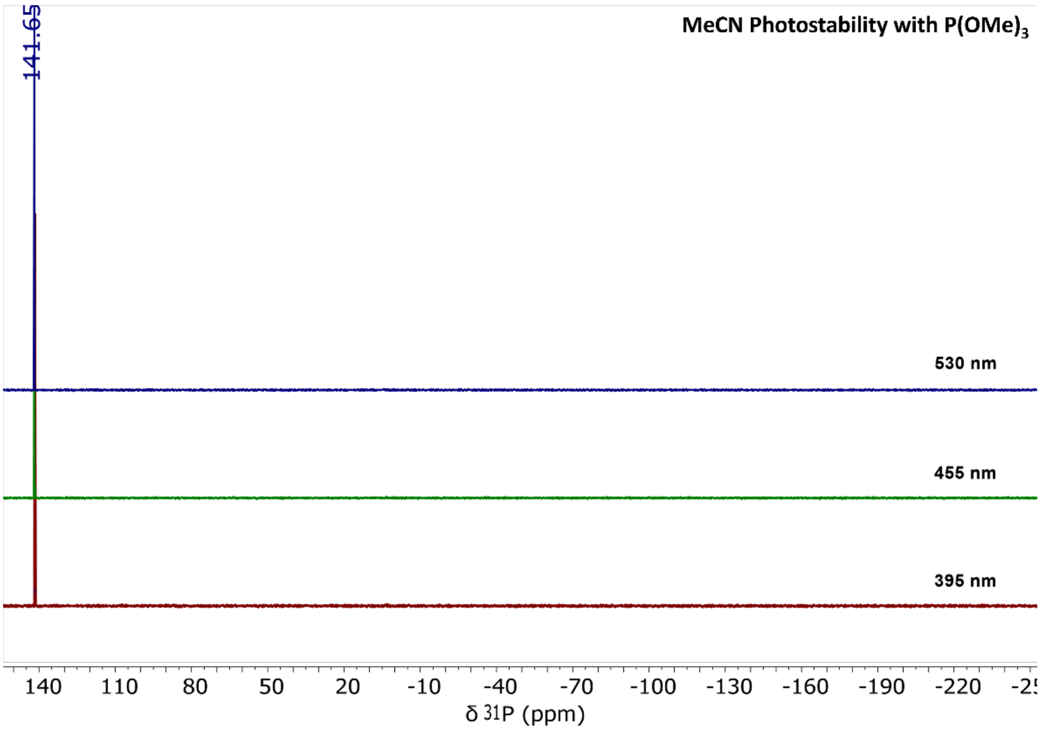

Figure S17: $^{31}P\{^{1}H\}$ NMR spectra of a mixture of **Acr•** with $P(OMe)_3$ in THF following irradiation by 395, 455 or 530 nm light. Assignment: δ = 141.7 ppm, $P(OMe)_3$. No significant differences were observed in the ^{1}H NMR spectra compared to the spectra run without $P(OMe)_3$.

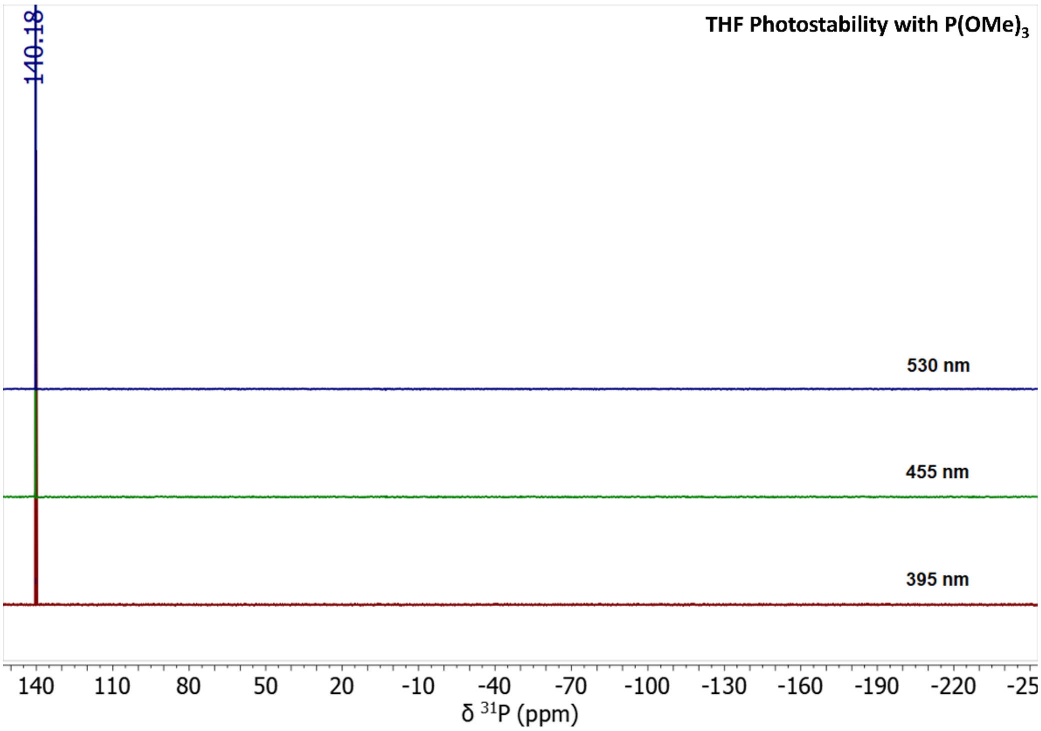

Figure S18: $^{31}P\{^{1}H\}$ NMR spectra of a mixture of **Acr•** with $P(OMe)_3$ in THF following irradiation by 395, 455 or 530 nm light. Assignment: δ = 140.2 ppm, $P(OMe)_3$. No significant differences were observed in the ^{1}H NMR spectra compared to the spectra run without $P(OMe)_3$.

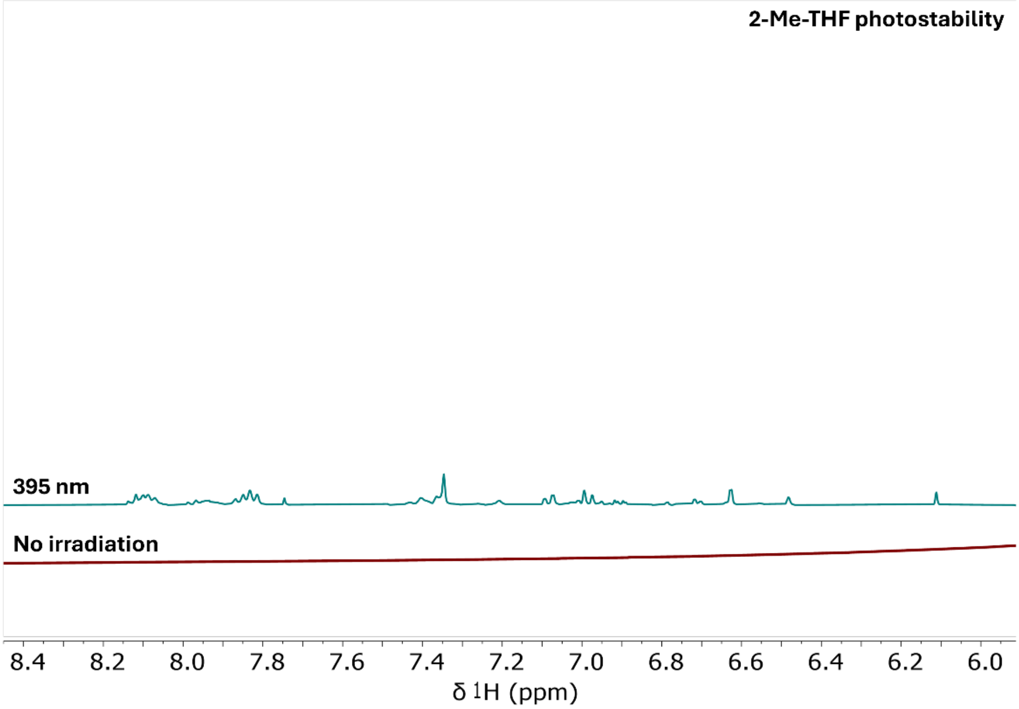

Figure S19: ^{1}H NMR spectra of **Acr•** in 2-Me-THF (5 mM) either without any irradiation or following irradiation at 395 nm for 16 h.

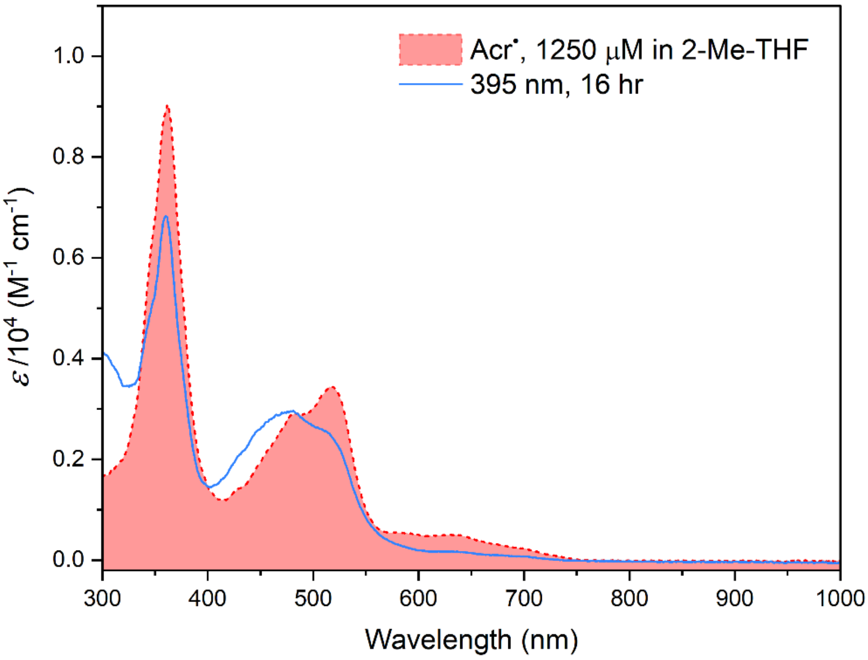

Figure S20: UV-vis spectra of **Acr•** in 2-Me-THF following irradiation by 395 nm light for 16 h.

3.2 THF decomposition mechanistic analysis

Based on the evidence provided by transient absorption experiments (see Section 5.3, below, and discussion in the main manuscript), as well as the fact that productive photoreactivity is observed under conditions where **Acr•** is fully photostable (see Section 3.3, below), it seems unlikely that photodecomposition products of **Acr•** under 395 nm irradiation in THF play a significant role in photoreactivity under these conditions (indeed, the fact that far superior performance is observed under equivalent conditions in MeCN, where significant photodecomposition is not observed, would seem to imply the opposite).

As such, the nature of this decomposition has not been investigated in detail. However, given the obvious solvent dependence, a direct photoreaction with the solvent seems likely. It is conspicuous that the reduced photostability of **Acr•** in THF *vs.* MeCN mirrors that of **Acr**$^+$, which is also prone to decomposition upon irradiation in THF (see Section 4.3, below). The latter can be attributed to photoinduced oxidation of THF, which is much less stable than MeCN towards strong oxidants (e.g. *[**Acr**$^+$]). In contrast with **Acr**$^+$, **Acr•** is a poor oxidant and instead usually acts as a reductant. Discussion of its excited state reactivity therefore tends to focus on the enhancement of its reducing power. However, photoexcitation will also significantly enhance the oxidising power of **Acr•**. ***Acr•** states, especially high energy ones, may therefore also be strong oxidants. In particular, while 395 nm excitation of **Acr•** is expected to initially generate primarily the D_9 excited state, it is also expected to generate a small fraction of the D_{10} state, which has much lower oscillator strength but is essentially identical in energy (see Table S8, below). The latter can be characterised as a charge transfer state from the mesityl group to the acridinyl core. As such, it has strong "Mes$^{•+}$" character, which is directly analogous to the well-known *[**Acr**$^+$] charge transfer excited state, and suggests a similar capacity to act as an excited state oxidant (note also that because THF is a solvent, static quenching can be assumed to apply, bypassing diffusion limitations). As such, we tentatively suggest that the poor photostability of **Acr•** under 395 nm irradiation in THF can be attributed to photoinduced oxidation of the solvent (albeit likely with very low quantum yield), followed by irreversible downstream reaction steps.

An alternative possibility is that **Acr•** photodecomposition is instead initiated *via* H atom transfer from the solvent to **Acr•**, which would be consistent with the weaker C–H bond found in THF than in MeCN (see Section 8). As such, some preliminary investigations have been performed as an initial test of this alternative hypothesis. However, so far these have been inconclusive. While calculations suggest that H atom transfer from THF to **Acr•** should become thermodynamically favourable upon photoexcitation, this is also true of MeCN, despite the much greater photostability of **Acr•** in this solvent. Photoinduced H atom transfer would also be accessible from ESs available upon excitation at longer wavelengths, where no photodecomposition is observed. Attempts have also been made to chemically trap the putative $[C_4H_7O]^•$ radical that would be generated upon H atom loss from THF. However, these investigations have been stymied by the lack of a suitable trapping reagent that would be sufficiently reactive towards $[C_4H_7O]^•$ yet unreactive towards **Acr•**/***Acr•**. For example, no reaction is observed in the presence of $P(OMe)_3$ as a radical trap (see Section 3.1), whereas styrene appears to interact directly with **Acr•**/***Acr•**.

3.2.1 Calculated H-atom transfer to Acr•

We calculated the thermodynamics of H-atom transfer to **Acr•** from both THF and MeCN molecules, namely the free energy changes of the **Acr•** + MeCN → **Acr**H + MeCN-deH• and **Acr•** + THF → **Acr**H + THF-deH• reactions (see Section 8.1 for the relevant DFT-optimised structures, MeCN-deH• and THF-deH• denote the dehydrogenated radical species). In both cases, the reaction is endergonic (THF, ΔG_0 = 29.4 kcal mol^{-1}; MeCN, ΔG_0 = 33.0 kcal mol^{-1}), when **Acr•** is considered in ground state. Given that the lowest excited states of **Acr•** is about 49 kcal/mol (580 nm) above the ground state, H-atom transfer processes are thermodynamically accessible from the excited states of the PC radical. However, photodecomposition was only observed in THF.

3.2.2 THF radical trapping experiments

To test whether photodecomposition of **Acr•** upon 395 nm photoirradiation in THF might originate from H atom abstraction from solvent, followed by functionalisation of the acridine core by the resulting THF-derived $[C_4H_7O]^•$ radical, solutions of **Acr•** (MeCN, 1.7 mM; THF, 5 mM) were irradiated by 395 nm light in the presence of styrene (50 mM or 100 mM) as a potential $[C_4H_7O]^•$ trap. However, these experiments did not provide any evidence of

new THF/styrene-derived products, despite it appearing that addition of styrene (either 50 or 100 mM concentration) does suppress the decomposition of **Acr•** in THF. Neither significant decomposition products, nor any new aromatic resonances attributable to styrene-derived products were detected by ^{1}H NMR spectroscopy after 395 nm irradiation of **Acr•** in the presence of styrene (Figure S21-Figure S22).

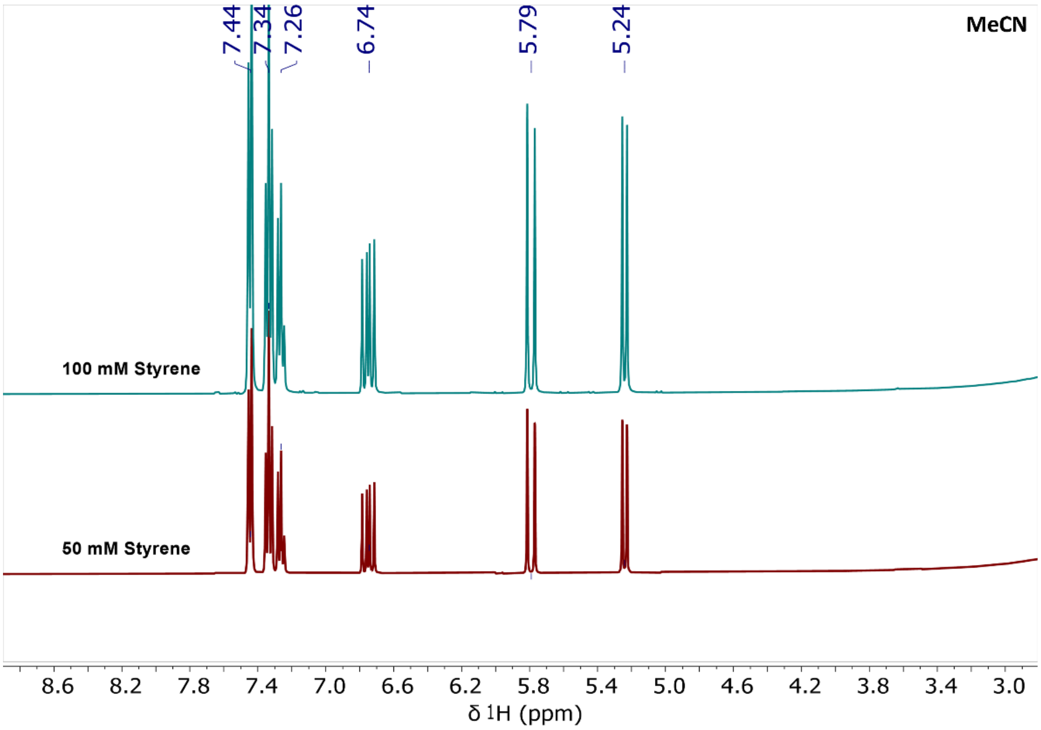

Figure S21: ^{1}H NMR spectrum of the reaction in MeCN between **Acr•** and styrene following irradiation at 395 nm for 16 h. Assignments: δ = 5.24, 5.79, 6.74, 7.26, 7.34, 7.44 ppm, styrene.

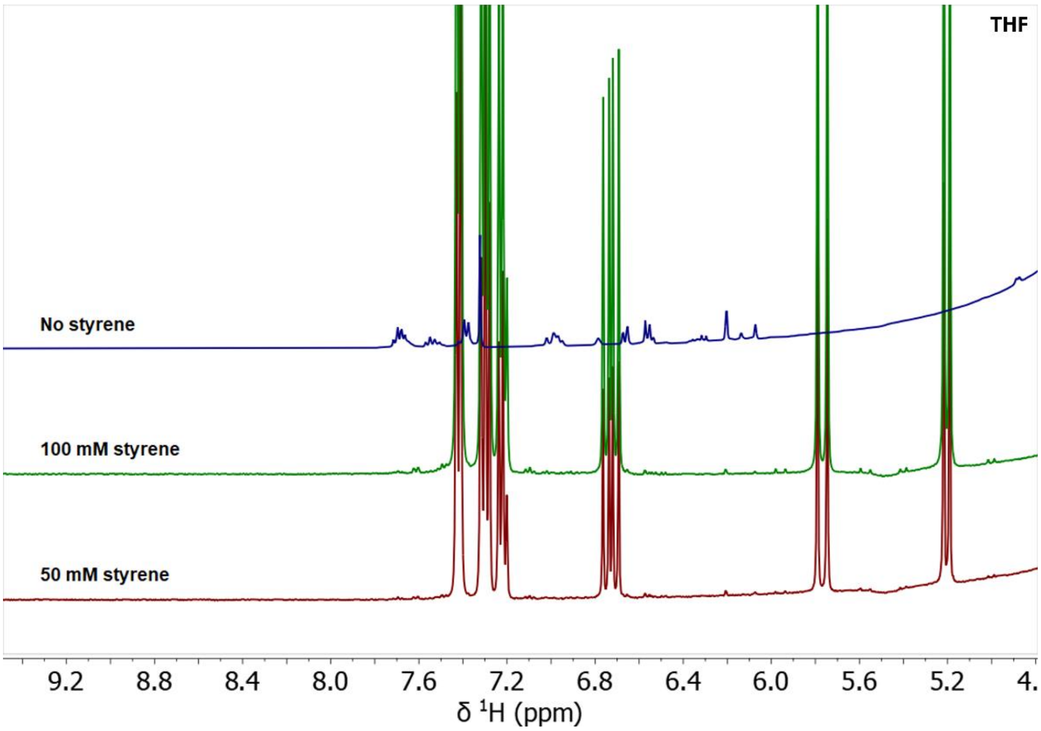

Figure S22: ^{1}H NMR spectrum of the reaction in THF between **Acr**$^{\bullet}$ and styrene following irradiation at 395 nm for 16 h. Assignments: δ = 5.20, 5.77, 6.73, 7.22, 7.30, 7.42 ppm, styrene. Spectra are normalised by the THF resonances (not pictured).

3.3 Photoreactivity with aryl chlorides

In either MeCN or in THF (500 μL), **Acr•** (MeCN, 1.7 mM; THF, 5 mM), ArCl (50 mM), and $P(OMe)_3$ (50 mM) were combined and either kept in the dark, or irradiated together in an NMR tube, for 16 h. For quantification, Ph_3PO (1 eq., 10 mM, 125 μL) was added and the mixture analysed by quantitative $^{31}P\{^1H\}$ NMR spectroscopy. % Yields are given *vs*. **Acr•** and were measured by integration against Ph_3PO.

In reactions where THF was used as the solvent and a productive reaction was observed, resonances consistent with dimethyl phosphite ($HPO(OMe)_2$) (δ = 10.1 ppm) were also observed. We did not observe $HPO(OMe)_2$ in unproductive reactions, indicating it is only produced 'downstream' of ArCl reduction, and this is attributed to photooxidation of $P(OMe)_3$ by [**Acr**]$^+$ generated in this step, rather than any direct photoreaction of **Acr•** (for more detailed discussion, including related control reactions, see Section 4).

The concentrations of **Acr•** used in these reactions were originally chosen to match our previous work studying other $PC^{\bullet-}$ (5 mM).[3] However, due to the low solubility of **Acr•** in MeCN (max. 3.4 mM), a lower concentration was required for studies in this solvent. Our previous studies using other $PC^{\bullet-}$ showed that reducing the $PC^{\bullet-}$ concentration in these reactions can lead to increased conversion (once normalised).[3] To test whether this might account for the improved reactivity observed in MeCN *vs.* THF, a test reaction was performed using the lower 1.7 mM concentration for the reaction in THF that gave the highest conversion (4-chlorobenzonitrile, 395 nm). However, this resulted in only a relatively modest increase in conversion (from 50% at 5 mM to 105% at 1.7 mM), that is still much lower than what is observed in MeCN (882%; see Table 1 of the main manuscript). For additional discussion of the effect of **Acr•**/substrate concentrations, see Section 3.3.1, below.

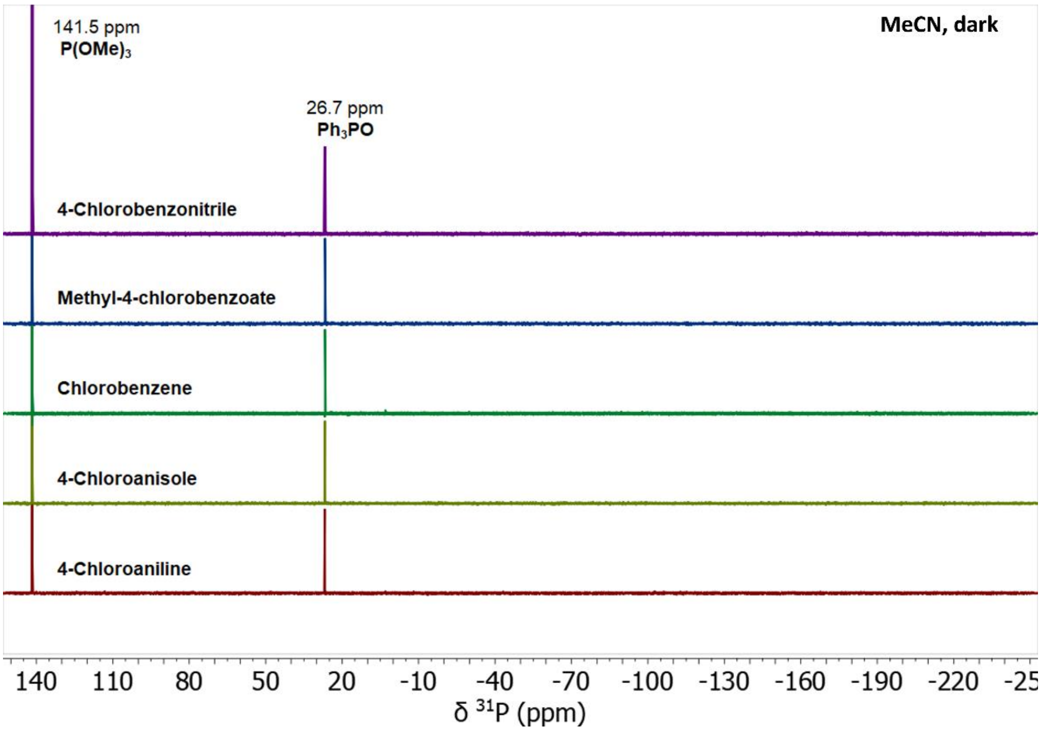

Figure S23: $^{31}P\{^1H\}$ NMR spectra of the reactions of Acr• in MeCN with various aryl chlorides in the presence of $P(OMe)_3$ after remaining in the dark for 16 h. Assignments: δ = 141.5 ppm, $P(OMe)_3$; δ = 26.7 ppm, Ph_3PO.

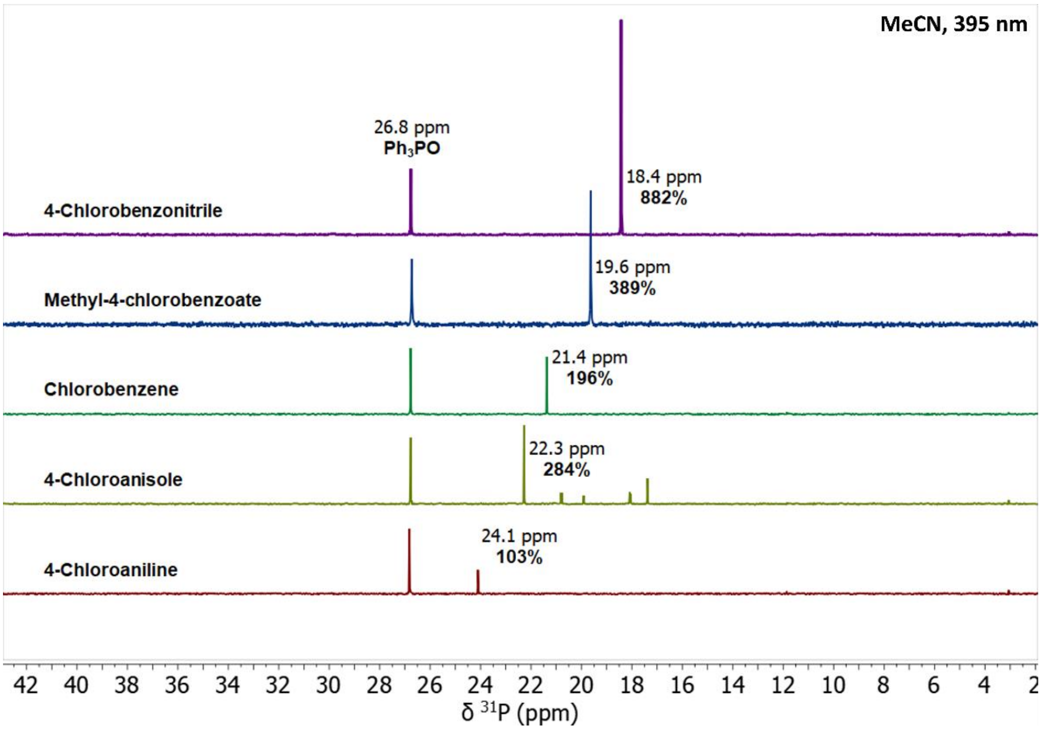

Figure S24: $^{31}P\{^1H\}$ NMR spectra of the reactions of **Acr•** in MeCN with various aryl chlorides in the presence of $P(OMe)_3$ following irradiation at 395 nm for 16 h. Assignments: δ = 26.8 ppm, Ph_3PO; δ = 24.1 ppm, $NH_3(C_6H_4)PO(OMe)_2$; δ = 22.3 ppm, $MeO(C_6H_4)PO(OMe)_2$; δ = 21.4 ppm, $PhPO(OMe)_2$; δ = 19.6 ppm, $MeO_2C(C_6H_4)PO(OMe)_2$; δ = 18.4 ppm, $NC(C_6H_4)PO(OMe)_2$. Not shown: δ = 141.5 ppm, $P(OMe)_3$. See Figure S25 for an expansion showing the additional products observed for the reaction with 4-chloroanisole.

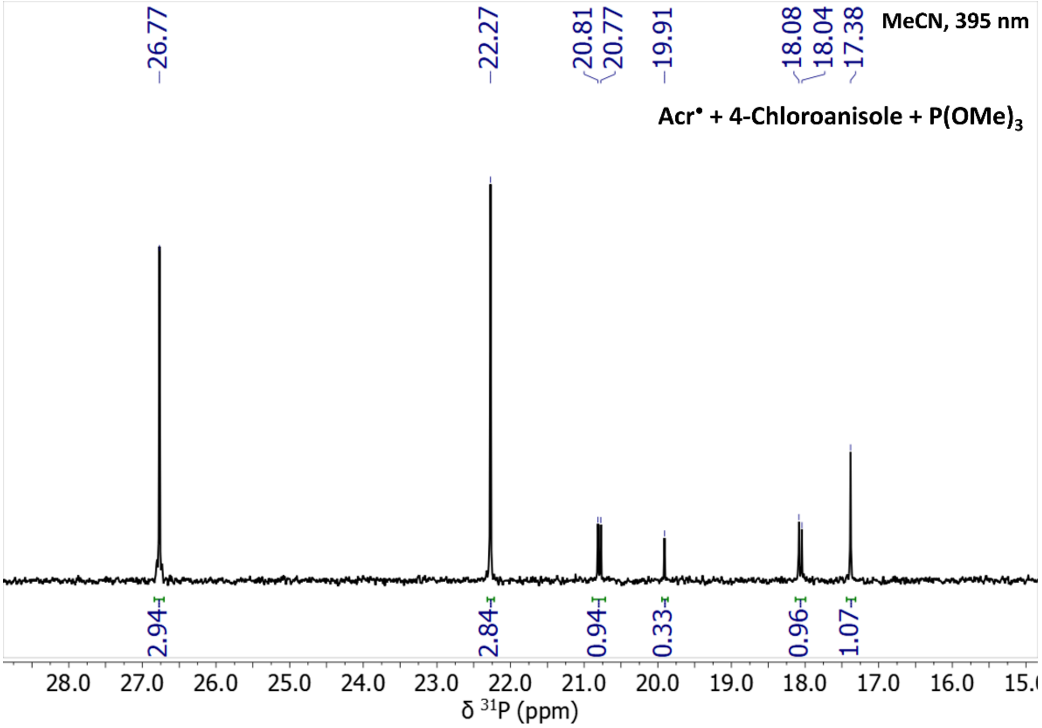

Figure S25: $^{31}P\{^1H\}$ NMR spectrum of the reaction of **Acr•** in MeCN with 4-chloroanisole in the presence of $P(OMe)_3$ following irradiation at 395 nm for 16 h. Assignments: δ = 26.8 ppm, Ph_3PO; δ = 22.3 ppm, $MeO(C_6H_4)PO(OMe)_2$.

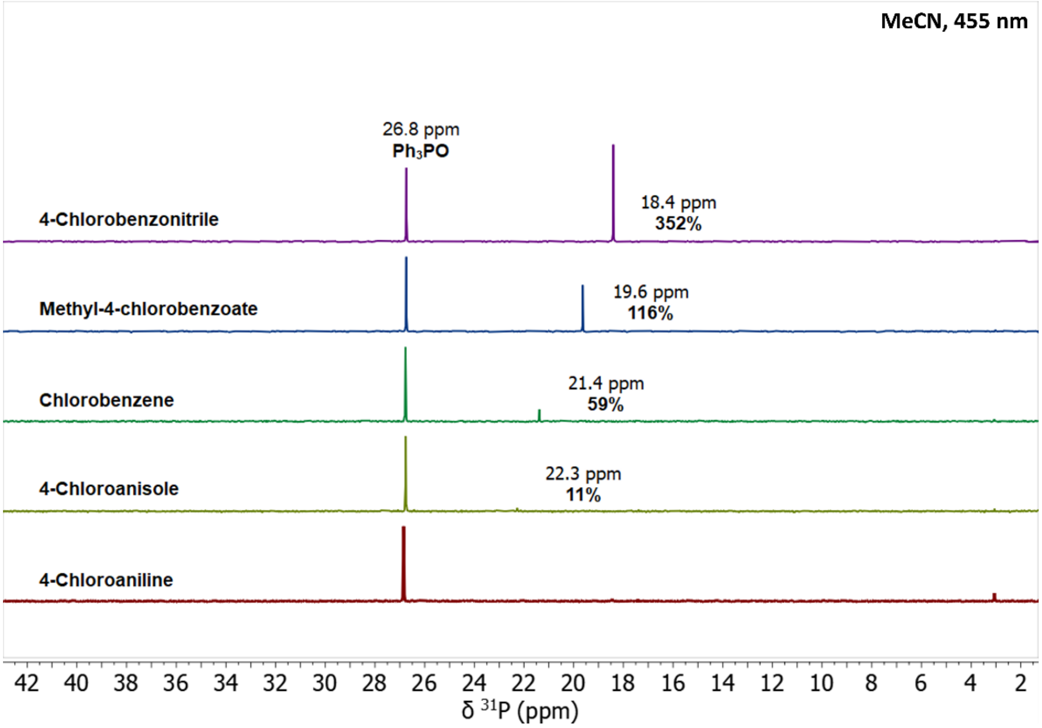

Figure S26: $^{31}P\{^{1}H\}$ NMR spectra of the reactions of **Acr•** in MeCN with various aryl chlorides in the presence of $P(OMe)_3$ following irradiation at 455 nm for 16 h. Assignments: δ = 26.8 ppm, Ph_3PO; δ = 22.3 ppm, $MeO(C_6H_4)PO(OMe)_2$; δ = 21.4 ppm, $PhPO(OMe)_2$; δ = 19.6 ppm, $MeO_2C(C_6H_4)PO(OMe)_2$; δ = 18.4 ppm, $NC(C_6H_4)PO(OMe)_2$. Not shown: δ = 141.5 ppm, $P(OMe)_3$.

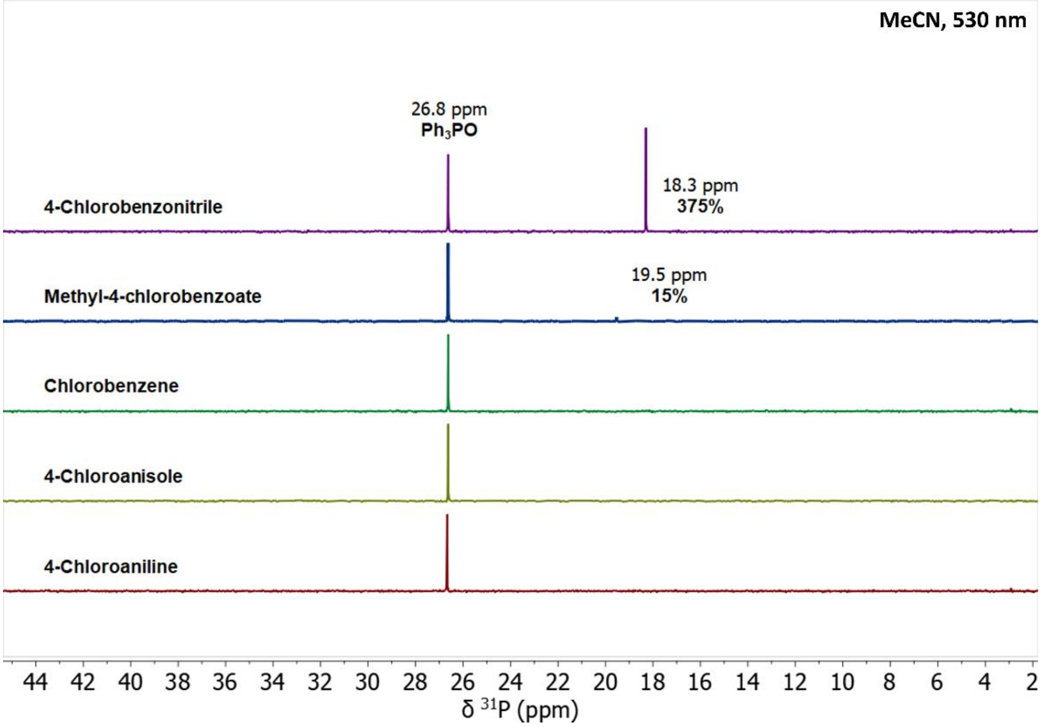

Figure S27: $^{31}P\{^{1}H\}$ NMR spectra of the reactions of **Acr•** in MeCN with various aryl chlorides in the presence of $P(OMe)_3$ following irradiation at 530 nm for 16 h. Assignments: δ = 26.8 ppm, Ph_3PO; δ = 19.5 ppm, $MeO_2C(C_6H_4)PO(OMe)_2$; δ = 18.3 ppm, $NC(C_6H_4)PO(OMe)_2$. Not shown: δ = 141.5 ppm, $P(OMe)_3$.

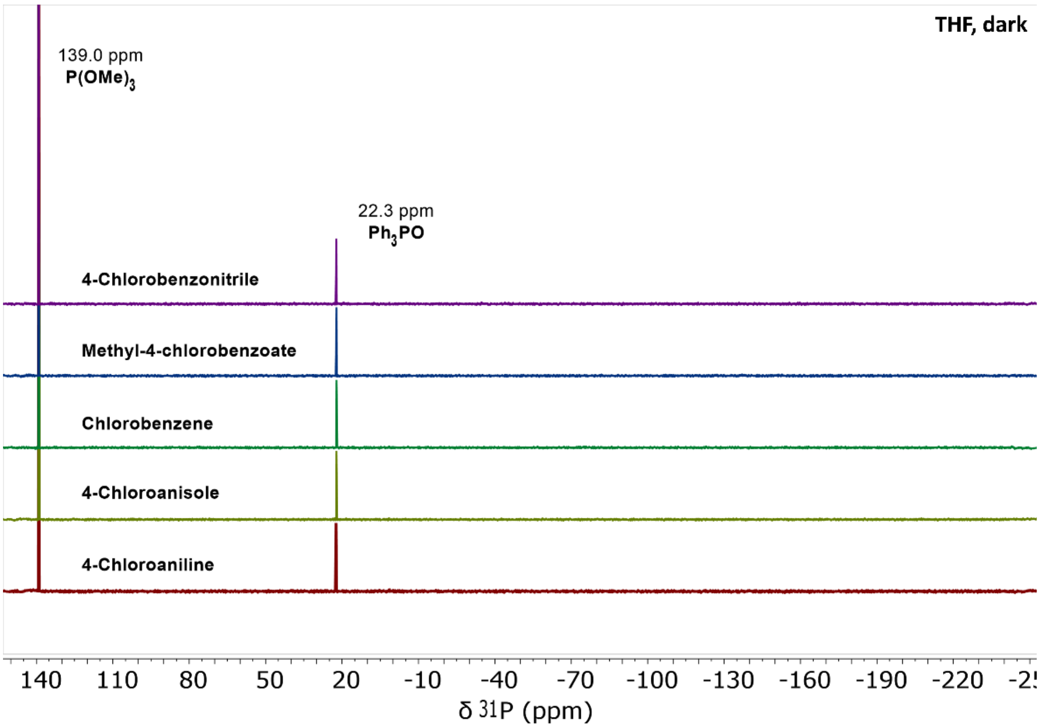

Figure S28: $^{31}P\{^1H\}$ NMR spectra of the reactions of Acr$^•$ in THF with various aryl chlorides in the presence of $P(OMe)_3$ after remaining in the dark for 16 h. Assignments: δ = 139.0 ppm, $P(OMe)_3$; δ = 22.3 ppm, Ph_3PO.

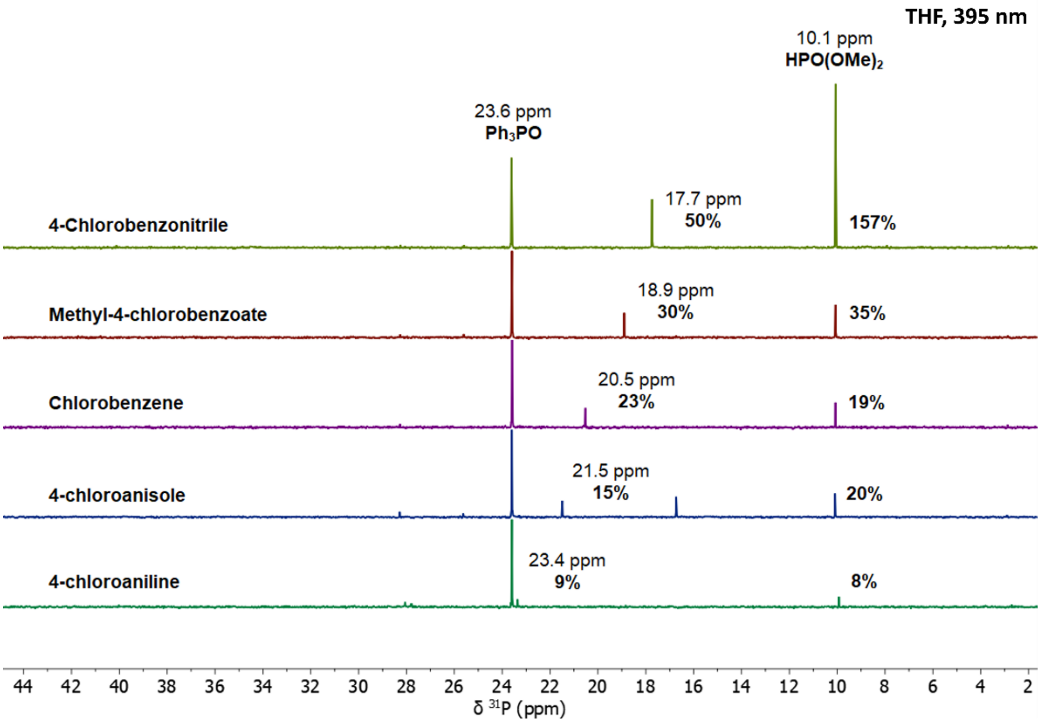

Figure S29: $^{31}P\{^1H\}$ NMR spectra of the reactions of **Acr$^•$** in THF with various aryl chlorides in the presence of $P(OMe)_3$ following irradiation at 395 nm for 16 h. Assignments: δ = 23.6 ppm, Ph_3PO; δ = 21.5 ppm, $MeO(C_6H_4)PO(OMe)_2$; δ = 23.4 ppm, $H_2N(C_6H_4)PO(OMe)_2$; δ = 20.5 ppm, $PhPO(OMe)_2$; δ = 18.9 ppm, $MeO_2C(C_6H_4)PO(OMe)_2$; δ = 17.7 ppm, $NC(C_6H_4)PO(OMe)_2$; δ = 10.1 ppm, $HPO(OMe)_2$. Not shown: δ = 140.4 ppm, $P(OMe)_3$.

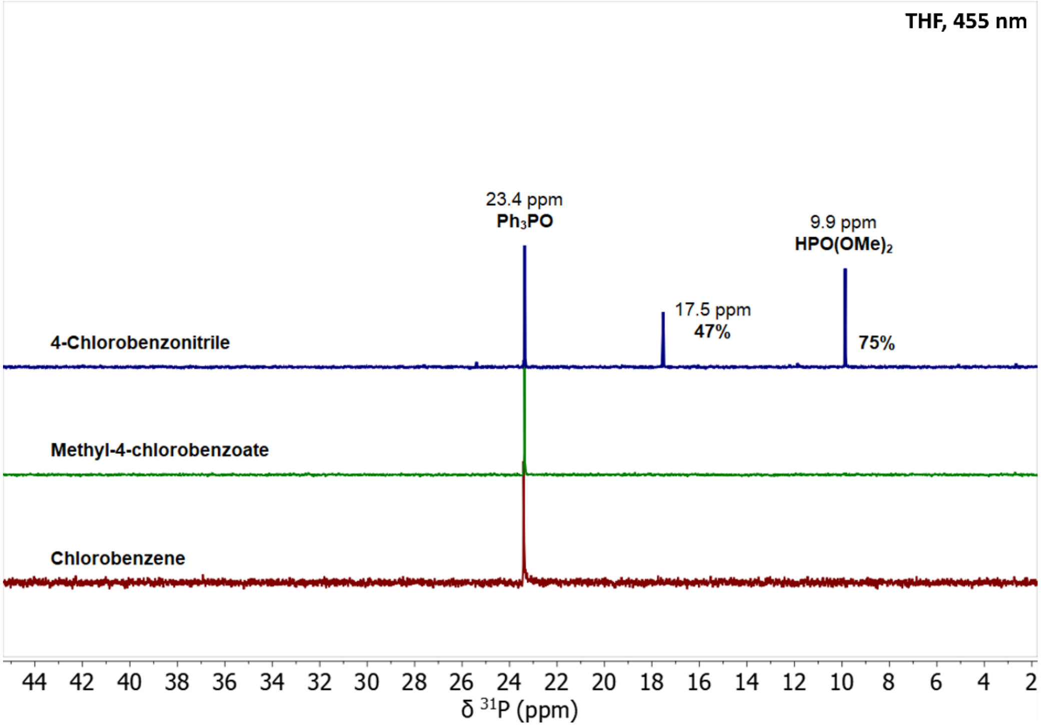

Figure S30: $^{31}P\{^{1}H\}$ NMR spectra of the reactions of **Acr•** in THF with various aryl chlorides in the presence of $P(OMe)_3$ following irradiation at 455 nm for 16 h. Assignments: δ = 23.4 ppm, Ph_3PO; δ = 17.5 ppm, $NC(C_6H_4)PO(OMe)_2$; δ = 9.9 ppm, $HPO(OMe)_2$. Not shown: δ = 140.4 ppm, $P(OMe)_3$.

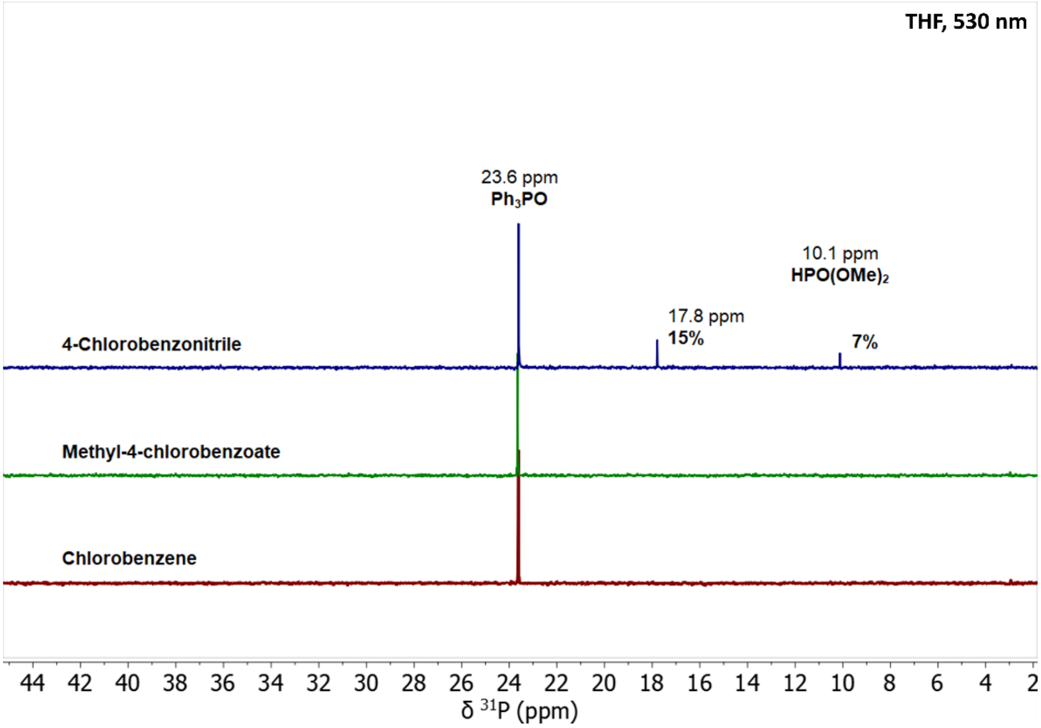

Figure S31: $^{31}P\{^{1}H\}$ NMR spectra of the reactions of **Acr•** in THF with various aryl chlorides in the presence of $P(OMe)_3$ following irradiation at 530 nm for 16 h. Assignments: δ = 23.6 ppm, Ph_3PO; δ = 17.8 ppm, $NC(C_6H_4)PO(OMe)_2$; δ = 10.1 ppm, $HPO(OMe)_2$. Not shown: δ = 140.4 ppm, $P(OMe)_3$.

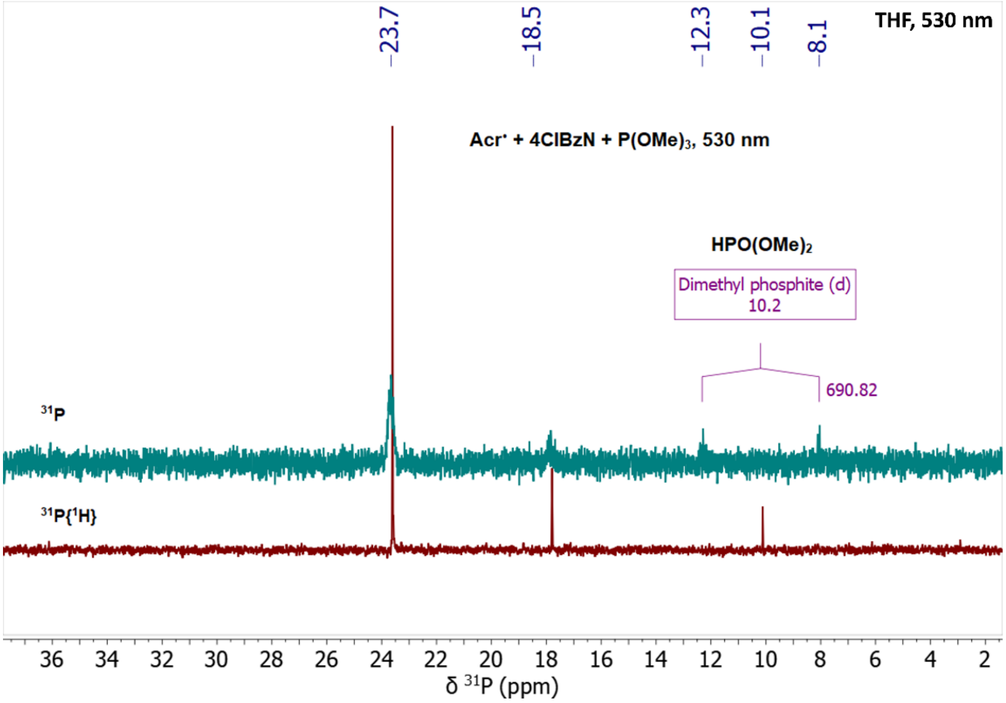

Figure S32: ^{31}P and ^{31}P{^{1}H} NMR spectra of the reaction of **Acr•** with 4-chlorobenzonitrile and $P(OMe)_3$ following irradiation at 530 nm for 16 h, with the resonance assigned to dimethyl phosphite ($HPO(OMe)_2$) highlighted.

3.3.1 Study of concentration effects on reactivity

In order to understand the role played by the concentrations of both **Acr$^{\bullet}$** and ArCl substrates, additional experiments were performed using different concentrations of both **Acr$^{\bullet}$** and 4-chlorobenzonitrile as a model substrate (see Table S1). Decreasing the concentration of **Acr$^{\bullet}$** increased the product yield relative to the photocatalyst (i.e. the turnover) significantly in MeCN (Figure S33, reaction with 4-chlorobenzonitrile in MeCN at 395 nm: [**Acr$^{\bullet}$**] = 1.7 mM, 882% yield; 0.5 mM, 1710%; 0.25 mM, 2400%). A very similar trend was observed in THF (Figure S34, reaction with 4-chlorobenzonitrile (4ClBzN) in THF at 395 nm: [**Acr$^{\bullet}$**] = 5 mM, 50% yield; 1.7 mM, 105%; 0.5 mM, 283%; 0.25 mM, 487%).

In line with expectations, product yield decreased with lower concentration of 4ClBzN, and increased with higher concentrations, up to the saturation limit at around 1 M (see Table S1, Chart S1). Note that approaching this limit, 4ClBzN is at such a concentration that it is likely the majority of **Acr$^{\bullet}$** molecules see it in their solvation shell on a purely statistical basis, implying the potential for static quenching regardless of 'formal' preassembly. Nevertheless, the initially rapid increase in % yield with increasing substrate concentration clearly begins to drop off once [4ClBzN] reaches approximately 250 mM, which would be consistent with a preassembly mechanism approaching saturation kinetics (provided the efficiency of the initial photoreduction event is the primary limiting factor for the observed conversions).

For reactions at higher [4ClBzN], it was necessary to increase the concentration of $P(OMe)_3$ to prevent it becoming the limiting reagent. Therefore, the concentration was increased to 500 mM for all reactions with [4ClBzN] > 50 mM. This leads to a significant increase in reaction yield (roughly 2.6x: from 882 % to 2,368 % upon increasing from 50 to 500 mM $P(OMe)_3$, using [4-ClBzN] ≈ 50 mM in both cases). Since control reactions indicate a lack of any productive direct (photo)reaction between $P(OMe)_3$ and **Acr$^{\bullet}$** or **[Acr]$^{+}$** (see Sections 3.1 and 4.3), this must be due to increased efficiency of productive steps occurring downstream of the initial aryl radical photo-reduction (and subsequent fragmentation). As such, the interpretation given above is somewhat tentative, as this observation implies that conversions are in fact dependent on not only the rate of the initial ArCl reduction, but also the rates of at least some 'downstream' reaction steps (e.g. productive ArCl$^{\bullet-}$ fragmentation and radical trapping, unproductive BET, *etc.*), which may also be concentration-dependent. (Note, for example, also the presence of both stoichiometric and catalytic ArCl reduction pathways, whose rates relative to one another are also likely to be concentration-dependent; see Section 3.4, below.)

Table S1: Yield of $NC(C_6H_4)PO(OMe)_2$ with different concentrations of **Acr$^{\bullet}$** and 4ClBzN following irradiation at 395 nm for 16 h. For [4-ClBzN] ≤ 50 mM, [$P(OMe)_3$] = 50 mM. For [4-ClBzN] > 50 mM, [$P(OMe)_3$] = 500 mM.

		**[4-Chlorobenzonitrile]; τ_{diff}; theoretical max yield vs. Acr$^{\bullet}$**							
		10 mM	25 mM	50 mM	54 mM	135 mM	270 mM	540 mM	970 mM
		5 ns	2 ns	1 ns	926 ps	370 ps	185 ps	93 ps	52 ps
**Solvent**	**[Acr$^{\bullet}$]**	588 %	1,471 %	2,941 %	3,176 %	7,941 %	15,881 %	29,412 %*	29,412 %*
MeCN	1.7 mM	302 %	482 %	882 %	2,368 %	4,903 %	6,115 %	5,797 %	8,081 %
	0.5 mM	-	-	1,710 %	-	-	-	-	-
	0.25 mM	-	-	2,400 %	-	-	-	-	-
		10 mM	25 mM	50 mM	73 mM	181 mM	363 mM	725 mM	1370 mM
		5 ns	2 ns	1 ns	685 ps	276 ps	138 ps	69 ps	36 ps
		588 %	1,471 %	2,941 %	4,294 %	10,646 %	21,352 %	29,412 %*	29,412 %*
THF	5 mM	-	-	50 %	-	-	-	-	-
	1.7 mM	83 %	65 %	105 %	448 %	769 %	1,018 %	1,504 %	2,083 %
	0.5 mM	-	-	283 %	-	-	-	-	-
	0.25 mM	-	-	487 %	-	-	-	-	-

* maximum yield is limited by [$P(OMe)_3$] rather than [4-chlorobenzonitrile].

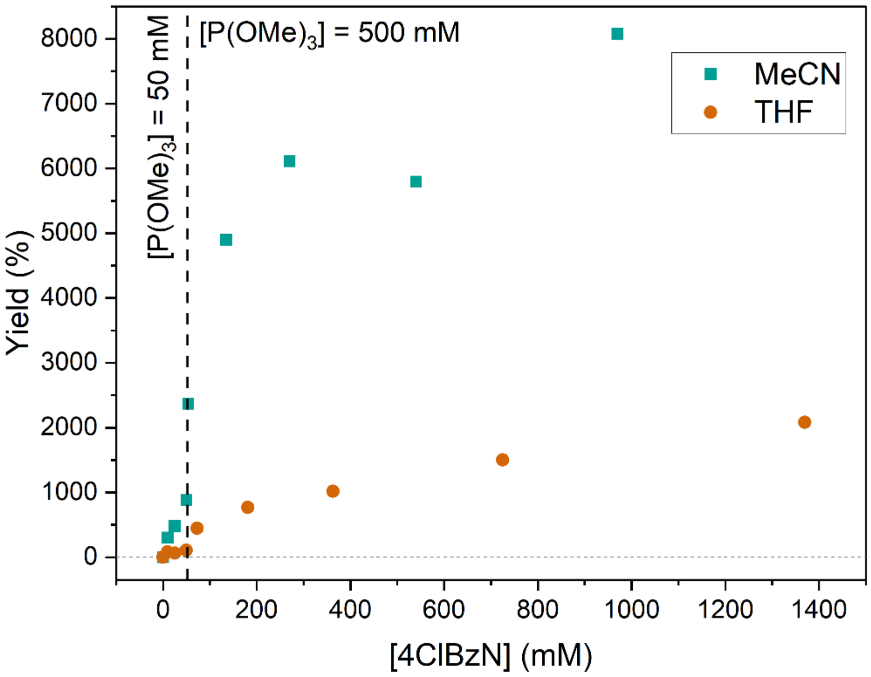

Chart S1: Yields of $NC(C_6H_4)PO(OMe)_2$ plotted against [4ClBzN]. Note that for [4ClBzN] ≤ 50 mM, $[P(OMe)_3]$ = 50 mM, for [4ClBzN] > 50 mM, $[P(OMe)_3]$ = 500 mM.

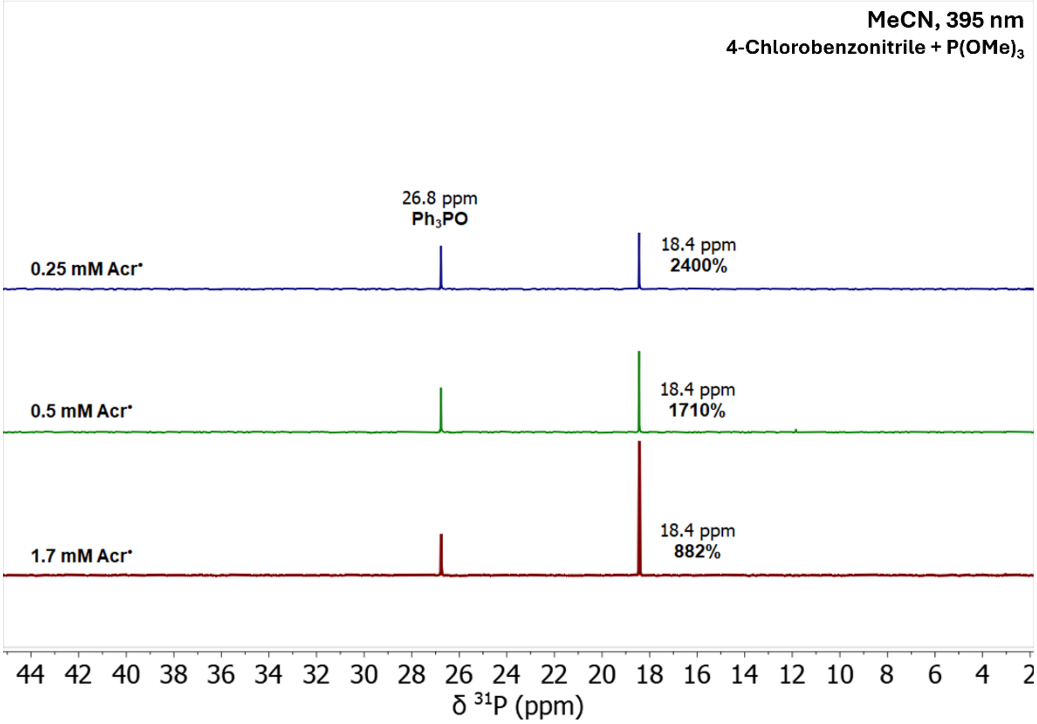

Figure S33: $^{31}P\{^{1}H\}$ NMR spectra of the reaction of **Acr•** at various concentrations in MeCN with 4-chlorobenzonitrile in the presence of $P(OMe)_3$ following irradiation at 395 nm for 16 h. Assignments: δ = 23.6 ppm, Ph_3PO; δ = 18.4 ppm, $NC(C_6H_4)PO(OMe)_2$. Not shown: δ = 140.5 ppm, $P(OMe)_3$.

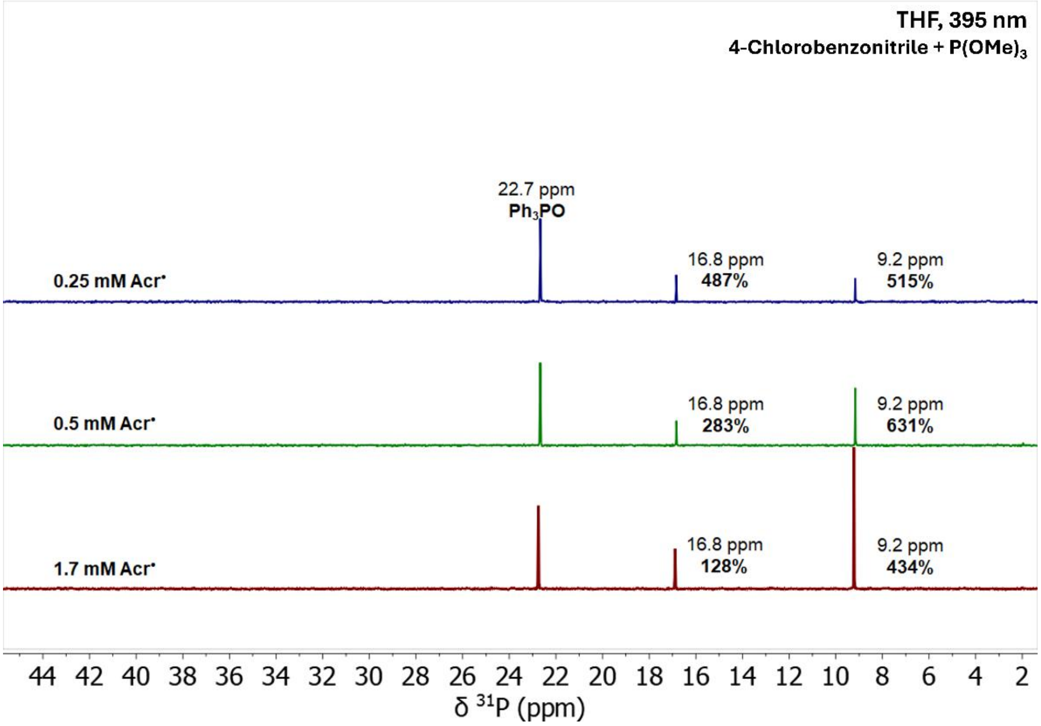

Figure S34: $^{31}P\{^{1}H\}$ NMR spectrum of the reaction of **Acr•** at various concentrations in THF with 4-chlorobenzonitrile in the presence of $P(OMe)_3$ following irradiation at 395 nm for 16 h. Assignments: δ = 22.7 ppm, Ph_3PO; δ = 16.8 ppm, $NC(C_6H_4)PO(OMe)_2$; δ = 9.2 ppm, $HPO(OMe)_2$. Not shown: δ = 140.5 ppm, $P(OMe)_3$.

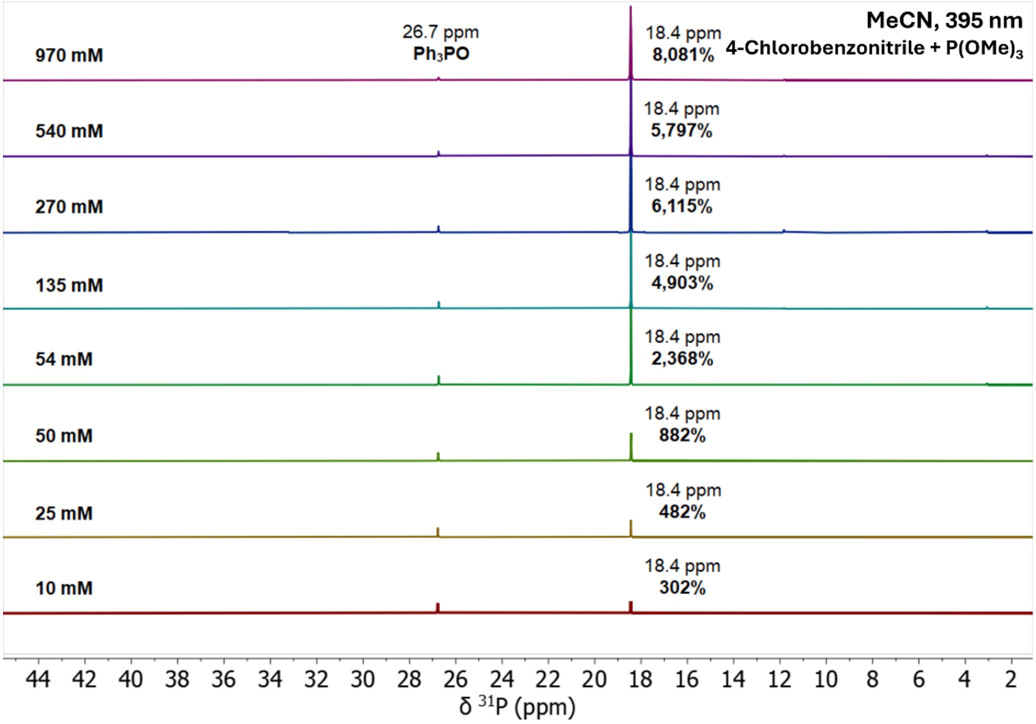

Figure S35: $^{31}P\{^{1}H\}$ NMR spectra of the reaction of **Acr•** (1.7 mM) in MeCN with 4-chlorobenzonitrile at various concentrations in the presence of $P(OMe)_3$ following irradiation at 395 nm for 16 h. Assignments: δ = 26.7 ppm, Ph_3PO; δ = 18.4 ppm, $NC(C_6H_4)PO(OMe)_2$. Not shown: δ = 140.5 ppm, $P(OMe)_3$.

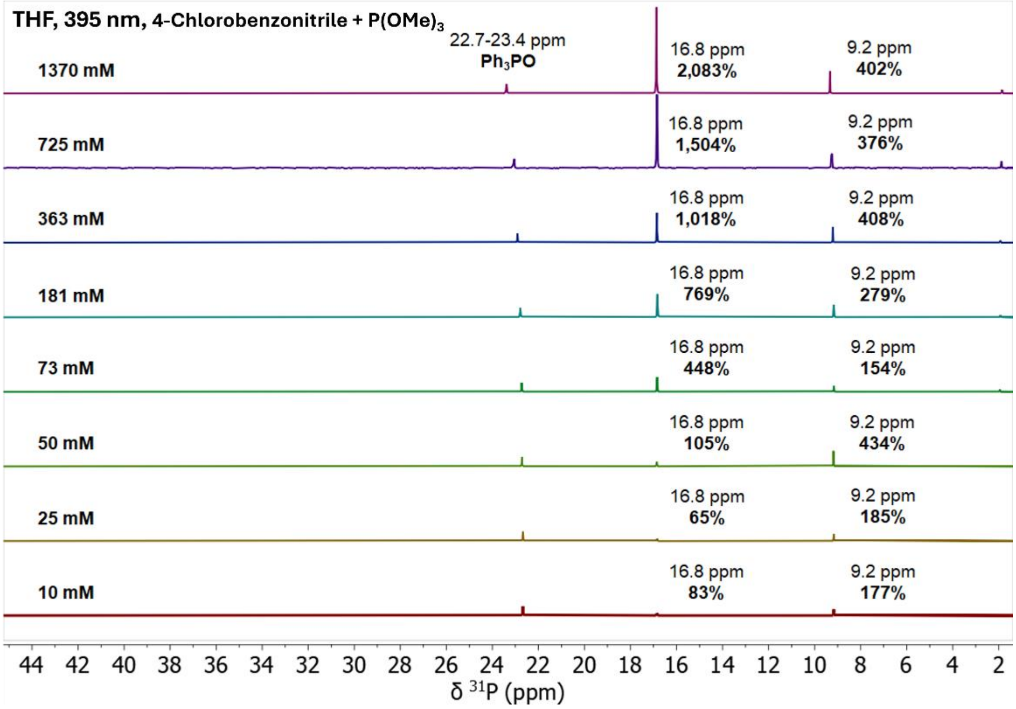

Figure S36: $^{31}P\{^{1}H\}$ NMR spectra of the reaction of **Acr•** (1.7 mM) in THF with 4-chlorobenzonitrile at various concentrations in the presence of $P(OMe)_3$ following irradiation at 395 nm for 16 h. Assignments: δ = 22.7-23.4 ppm, Ph_3PO; δ = 16.8 ppm, $NC(C_6H_4)PO(OMe)_2$; δ = 9.2 ppm, $HPO(OMe)_2$. Not shown: δ = 140.5 ppm, $P(OMe)_3$.

3.4 Super-stoichiometric reaction yields

We note that several of the reactions discussed afford superstoichiometric product yields, when we did not include a sacrificial reductant to regenerate the photocatalyst. We have reported similar behaviour in phosphite trapping experiments in the past, which we attributed to re-reduction of the photocatalyst by a phosphoranyl intermediate that was formed after addition of phosphite to the aryl radical.[3] This then provides an alternative route to eventually produce the same product by formal abstraction of a methyl cation by chloride, instead of homolytic cleavage of the Me–O bond. As the phosphoranyl should be similarly capable of reducing **Acr⁺** to **Acr•**, we propose that an equivalent mechanism results in superstoichiometric reactivity in **Acr•**, shown in Figure S37.

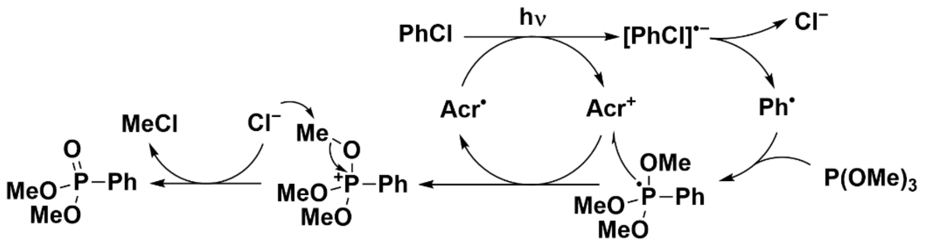

Figure S37: The proposed mechanism of re-reduction of **Acr⁺** by a phosphoranyl radical intermediate to form the active **Acr•** and eventually the aryl phosphonate product.

4 [Acr]$^+$[BF$_4$]$^-$ reactivity

4.1 NMR spectrum of [Acr]$^+$[BF$_4$]$^-$

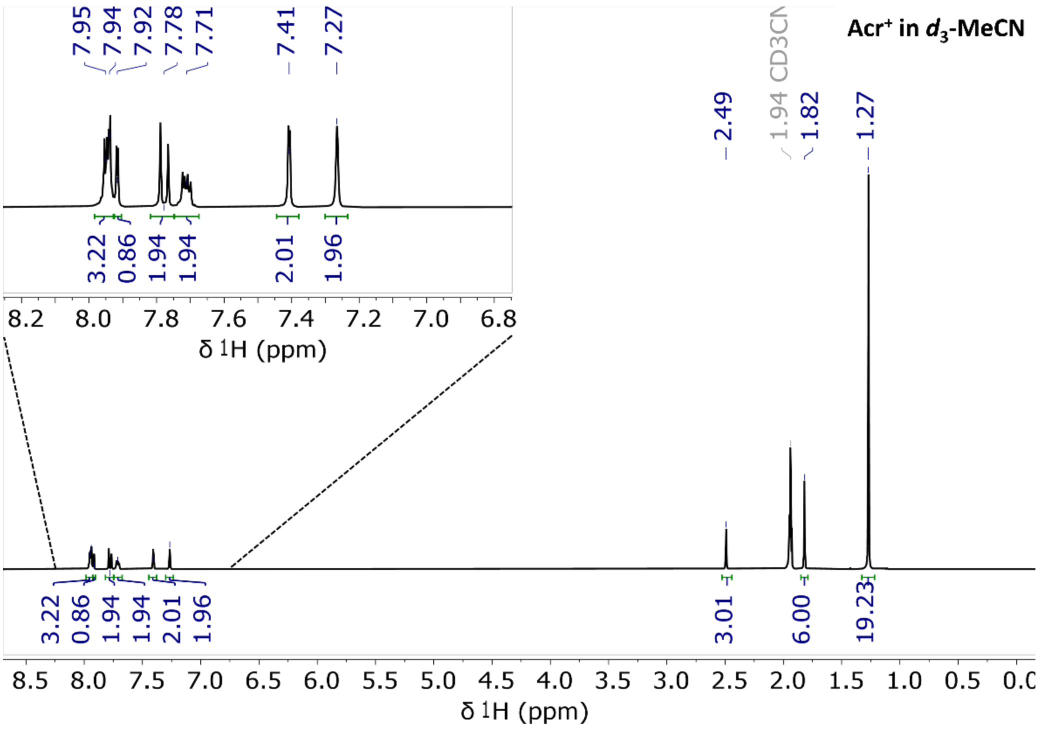

Figure S38: ^{1}H NMR spectrum of [**Acr**]$^+$[BF$_4$]$^-$ as recorded in d_3-MeCN. Inset: an expansion showing the aromatic region of the spectrum between 6.75 and 8.25 ppm.

4.2 UV-vis spectra of $[Acr]^{+}[BF_4]^{-}$

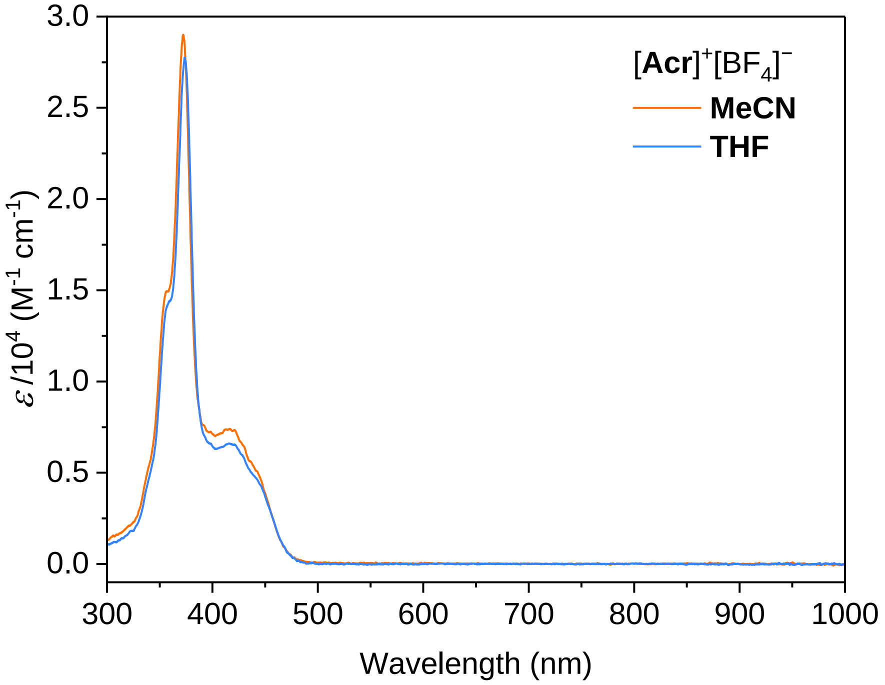

Figure S39: UV-vis spectra of [**Acr**]$^{+}$[BF$_4$]$^{-}$ recorded at 298 K on MeCN (orange) or THF (blue) solutions of 0.333 μM concentration.

4.3 Photostability

In MeCN or THF, [**Acr**]$^+$[BF_4]$^-$ (5 mM) was irradiated by 395 or 455 nm light for 16 h (note that [**Acr**]$^+$ does not absorb at 530 nm; see Figure S39). Significant photodecomposition was observed in the presence of P(OMe)$_3$ (50 mM) and/or THF by UV-vis. By ^{1}H NMR, no [**Acr**]$^+$ was detected following irradiation in either solvent (Figure S41 and Figure S42), despite the UV-vis indicating that there was minimal change in MeCN. It is possible that a small amount of solvent oxidation took place, and that degenerate electron exchange then resulted in broadening of the NMR signals. In the presence of P(OMe)$_3$ (Figure S43 and Figure S45), a reaction was also observed to occur by NMR, most notably in the ^{31}P{^{1}H} NMR spectra where several new ^{31}P-containing species were detected, consistent with photooxidation of P(OMe)$_3$ by ***Acr**$^+$ (Figure S44 and Figure S46). One of the new species observed is consistent with HPO(OMe)$_2$ (MeCN, δ = 11.9 ppm; THF, δ = 9.8 ppm), which was also observed during certain reactions of **Acr**$^•$ with ArCl/P(OMe)$_3$ in THF, despite no photoreaction occurring between **Acr**$^•$ and P(OMe)$_3$ in the absence of ArCl (see Section 3, above). It is therefore likely that the HPO(OMe)$_2$ observed in those reactions was the result of photooxidation of P(OMe)$_3$ by the **Acr**$^+$ generated upon initial photoelectron transfer from ***Acr**$^•$. Note, however, that in none of the reactions of **Acr**$^+$ presented here was HPO(OMe)$_2$ the major phosphorus-containing product.

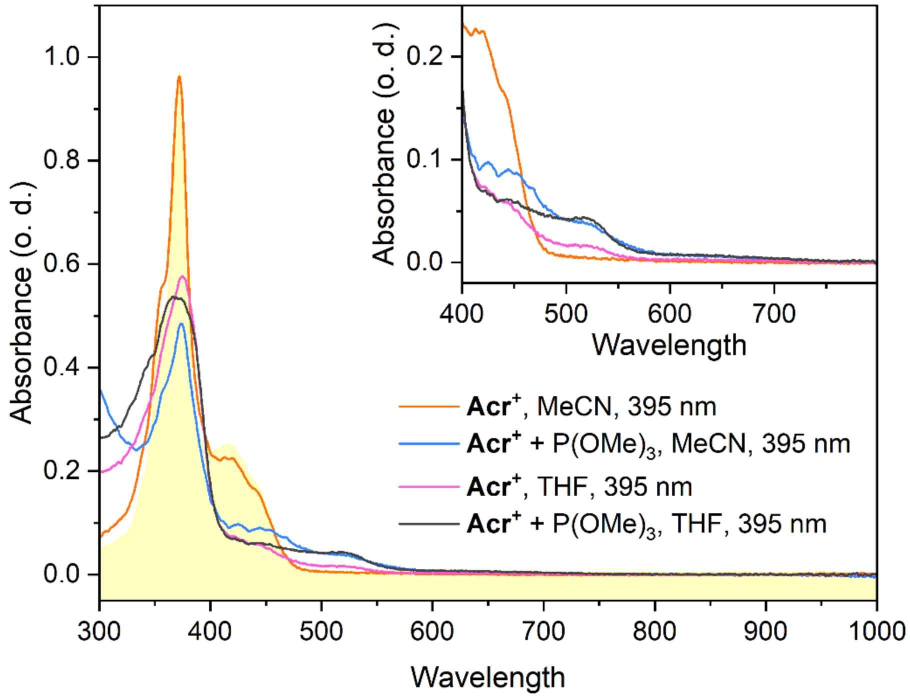

Figure S40: UV-vis spectra of [**Acr**]$^+$ solutions (5 mM) after irradiation with light of 395 nm in MeCN or THF for 16 h, either in the presence or absence of P(OMe)$_3$ (50 mM). Solutions were diluted to a nominal 333 μM concentration before recording. The absorbance spectrum of [**Acr**]$^+$ is included as a pale yellow underlay for reference.

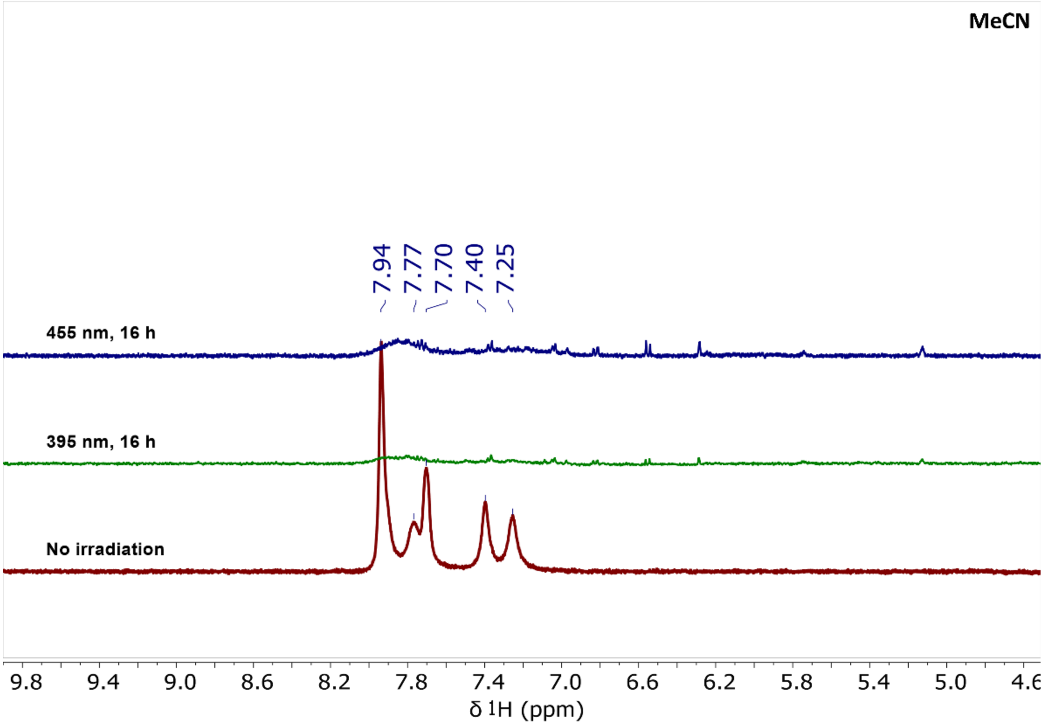

Figure S41: ^{1}H NMR spectrum of [**Acr**]$^+$[BF_4]$^-$ in MeCN following irradiation for 16 h.

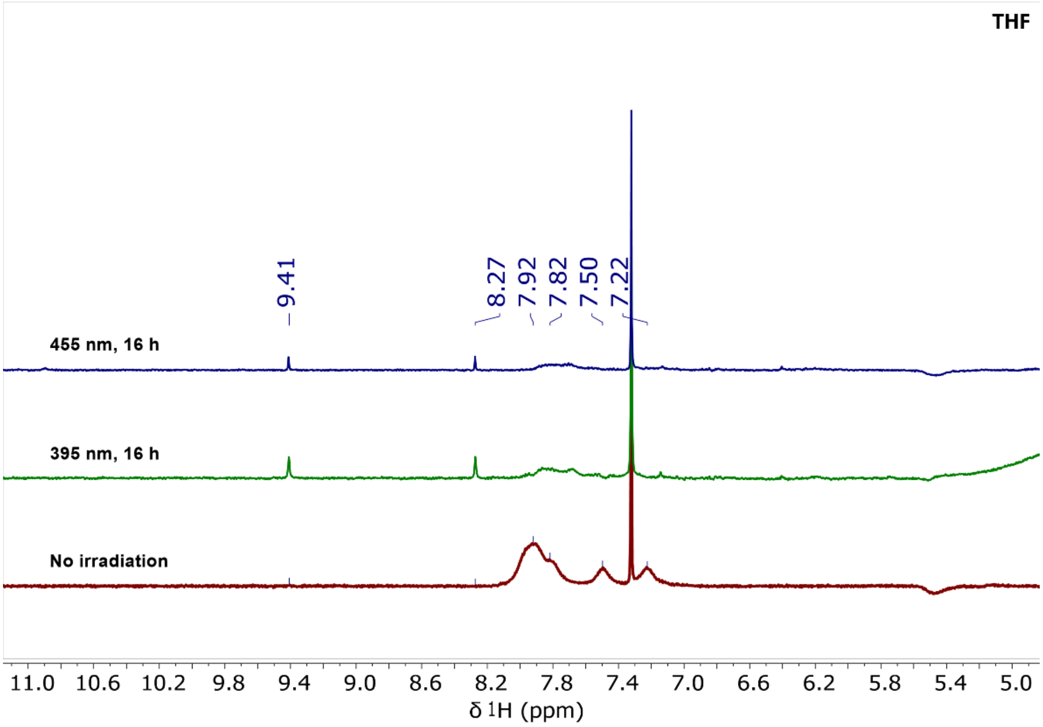

Figure S42: ^{1}H NMR spectrum of [**Acr**]$^+$[BF_4]$^-$ in THF following irradiation for 16 h.

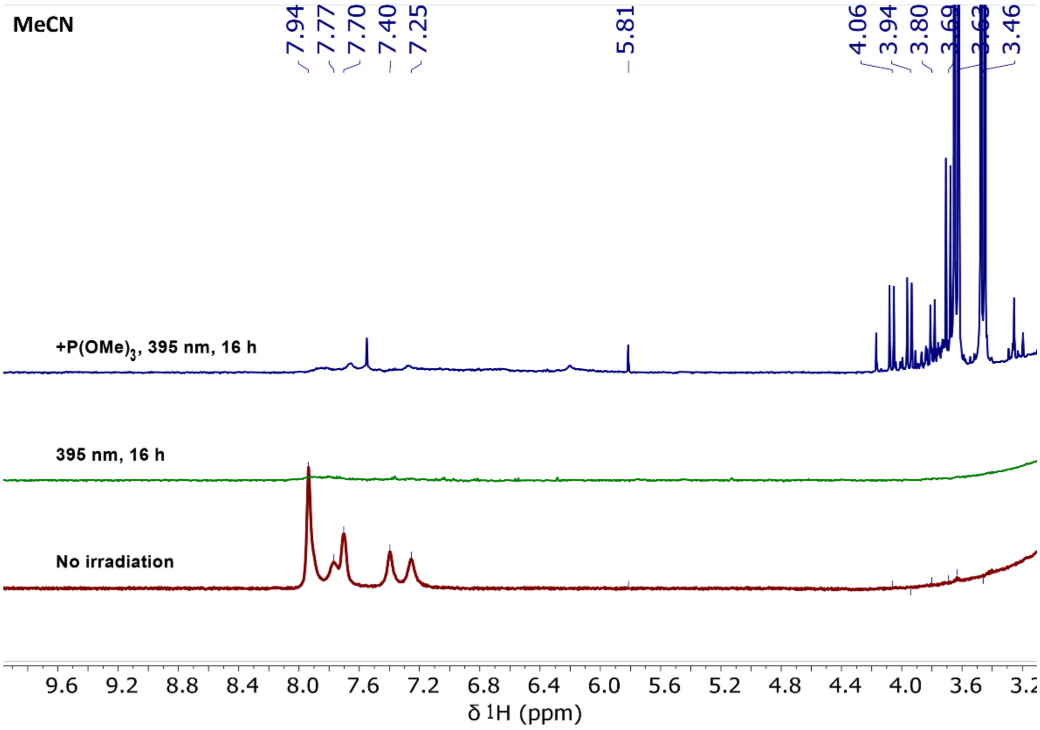

Figure S43: ^{1}H NMR spectrum showing the products of the reaction in MeCN between [**Acr**]$^{+}$ and $P(OMe)_3$ following irradiation at 395 nm for 16 h.

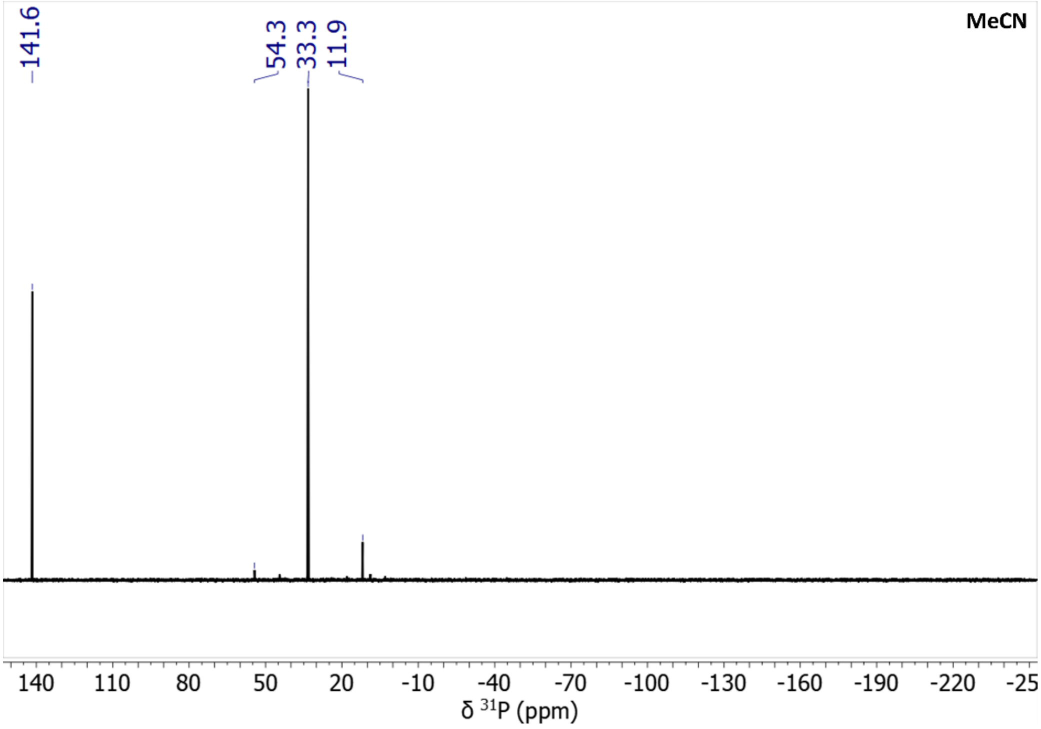

Figure S44: $^{31}P\{^{1}H\}$ NMR spectrum showing the products of the reaction in MeCN between [**Acr**]$^{+}$ and $P(OMe)_3$ following irradiation at 395 nm for 16 h. Assignments: δ = 141.6 ppm, $P(OMe)_3$; δ = 11.9 ppm, $HPO(OMe)_2$.

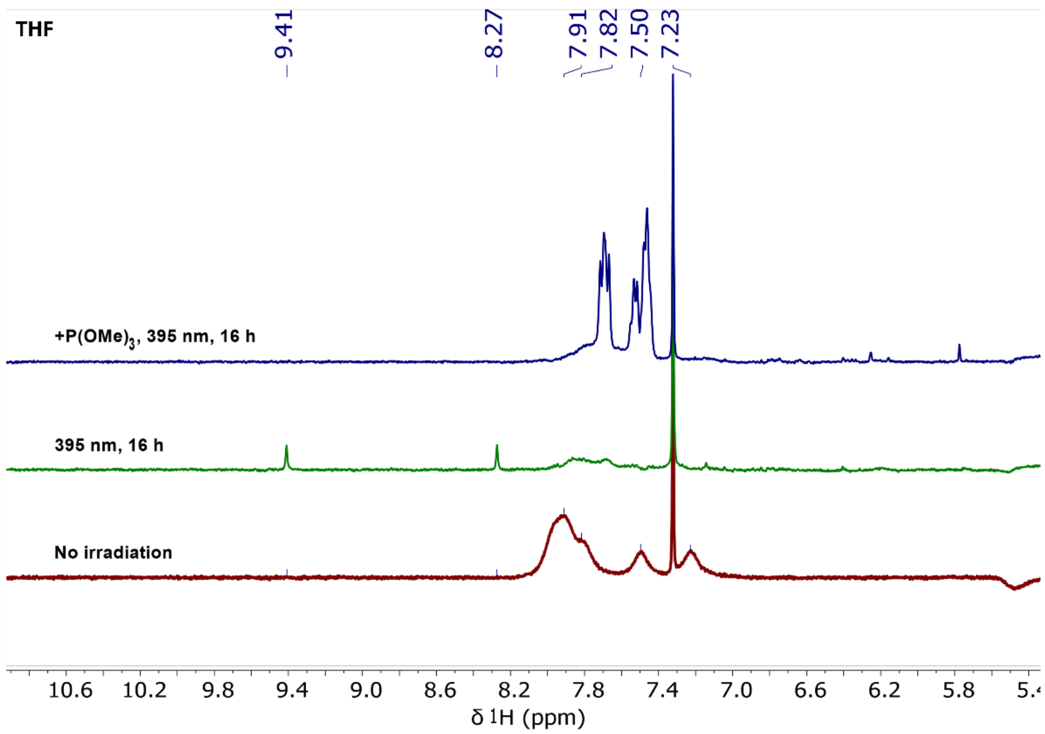

Figure S45: ^{1}H NMR spectrum showing the products of the reaction in THF between [**Acr**]$^{+}$ and $P(OMe)_3$ following irradiation at 395 nm for 16 h. Region <5.4 ppm not shown due to intense H_8-THF resonance obscuring detail.

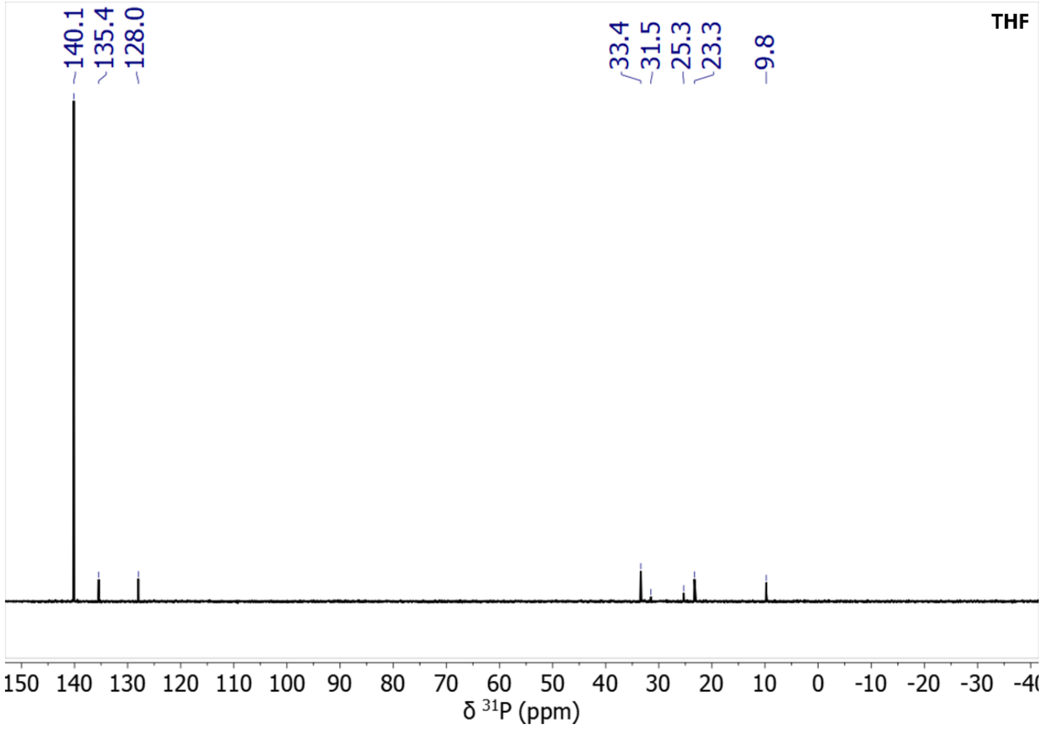

Figure S46: $^{31}P\{^{1}H\}$ NMR spectrum showing the products of the reaction in MeCN between [**Acr**]$^{+}$ and $P(OMe)_3$ following irradiation at 395 nm for 16 h. Assignments: δ = 140.1 ppm, $P(OMe)_3$; δ = 9.8 ppm, $HPO(OMe)_2$.

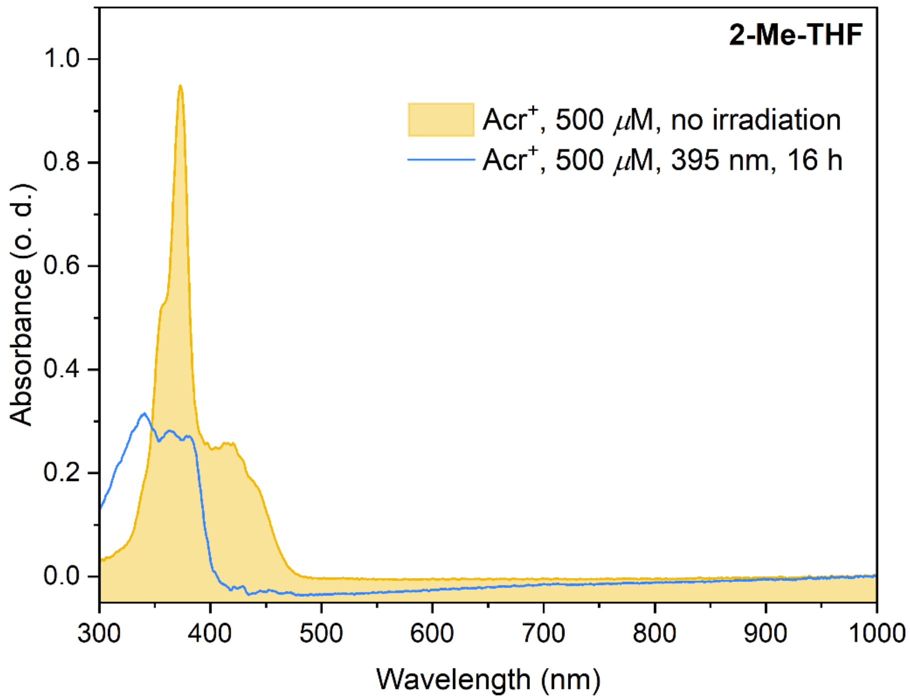

Figure S47: UV-vis spectra of [Acr]$^+$ solutions (5 mM) after irradiation with light of 395 nm in 2-Me-THF for 16 h. Solutions were diluted to a nominal 500 μM concentration before recording. The absorbance spectrum of [Acr]$^+$ in the same solvent is included as a pale yellow underlay for reference.

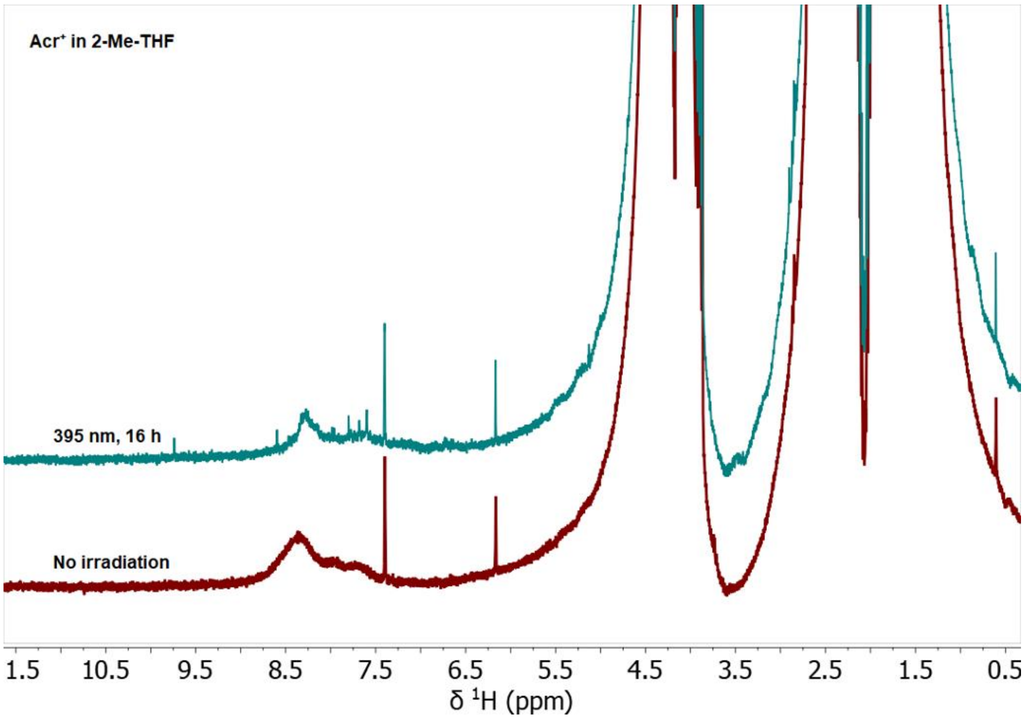

Figure S48: ^{1}H NMR spectra of [**Acr**]$^+$ in 2-Me-THF before and after irradiation with light of 395 nm for 16 h.

4.4 Photoreactivity with aryl chlorides

When [**Acr**]$^+$[BF_4]$^-$ was irradiated in the presence of both ArCl and P(OMe)$_3$, small quantities of aryl phosphonate products were also produced, echoing the photoreactivity of **Acr**$^\bullet$ (Section 3.3). However, the yields of these products were much lower (or non-existent) when starting from [**Acr**]$^+$ compared to **Acr**$^\bullet$, suggesting that this pathway is unlikely to be a significant contributor to the conversions measured when starting from **Acr**$^\bullet$. Furthermore, in addition to the phosphonate products, other unidentified species were consistently observed in the ^{31}P NMR spectra which were not observed when starting from **Acr**$^\bullet$.

In either MeCN or in THF (500 μL), [**Acr**]$^+$[BF_4]$^-$ (5 mM), ArCl (50 mM) and P(OMe)$_3$ (50 mM) were irradiated together in an NMR tube for 16 h. For quantification, Ph_3PO (1 eq., 10 mM, 125 μL) was added and the mixture sent for quantitative ^{31}P{^{1}H} NMR analysis (Figure S49 and Figure S50). % Yields are given *vs*. [**Acr**]$^+$ and were measured by integration against Ph_3PO.

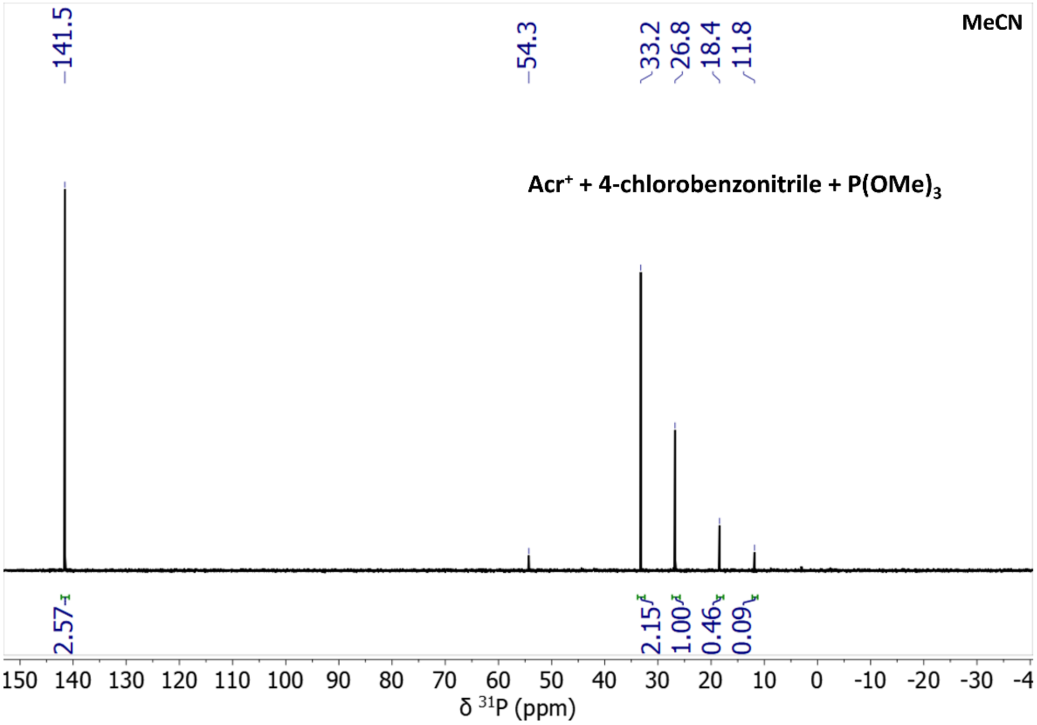

Figure S49: ^{31}P{^{1}H} NMR spectrum showing the products of the reaction in MeCN between [**Acr**]$^+$, 4-chlorobenzonitrile and P(OMe)$_3$ following irradiation at 395 nm for 16 h. Assignments: δ = 141.5 ppm, P(OMe)$_3$; δ = 26.8 ppm, Ph_3PO; δ = 18.4, NC(C_6H_4)PO(OMe)$_2$; δ = 11.8 ppm, HPO(OMe)$_2$. Yield of NC(C_6H_4)PO(OMe)$_2$: 46%.

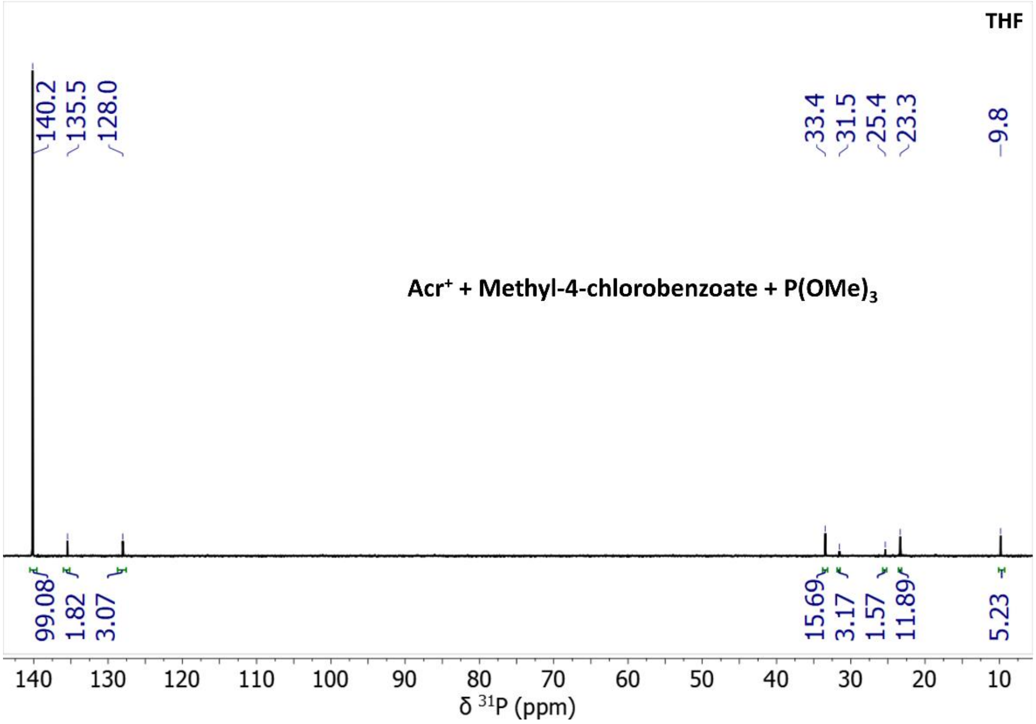

Figure S50: $^{31}P\{^{1}H\}$ NMR spectrum showing the products of the reaction in THF between [**Acr**]$^{+}$, methyl-4-chlorobenzonitrile and $P(OMe)_3$ following irradiation at 395 nm for 16 h. Assignments: δ = 140.2 ppm, $P(OMe)_3$; δ = 23.3 ppm, Ph_3PO. No product resonance was observed.

5 Fluorescence and transient absorption spectroscopy

5.1 Fluorescence spectroscopy

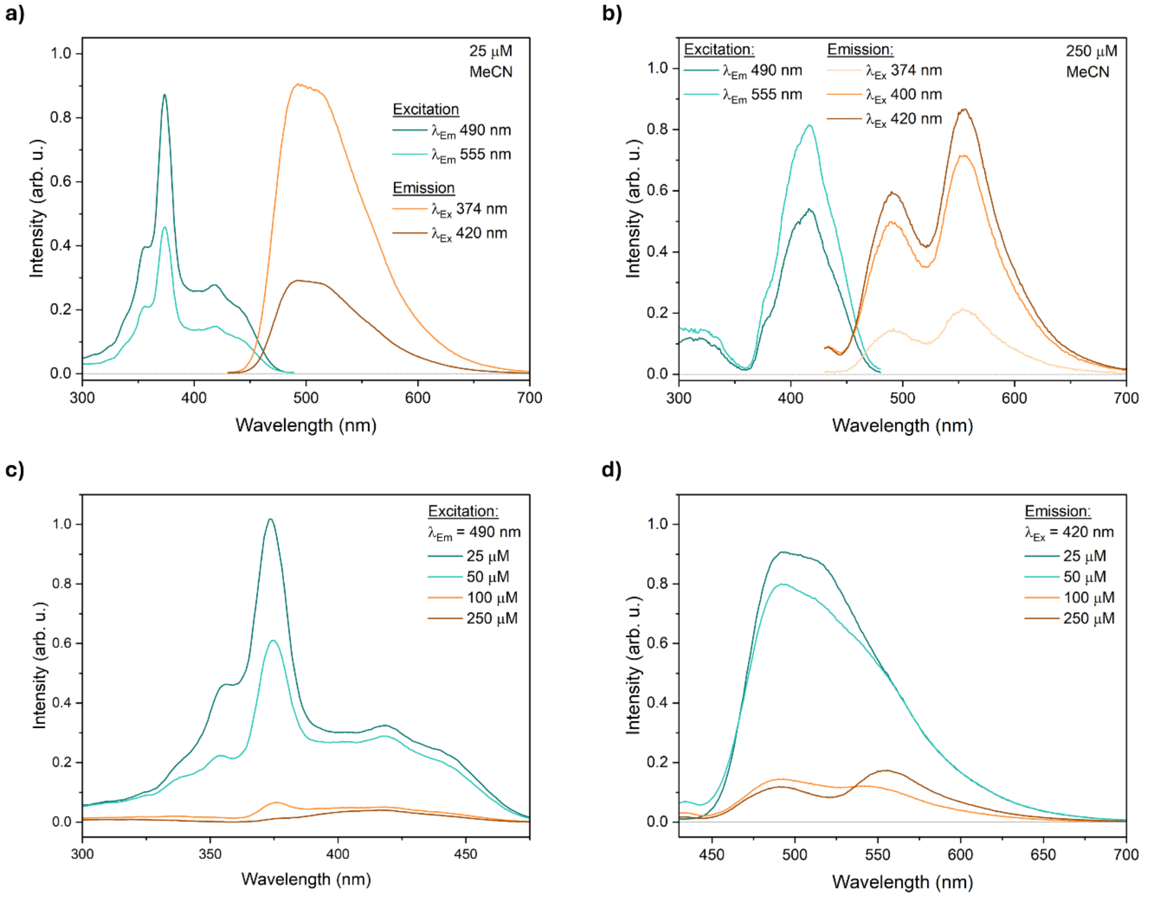

Figure S51: Fluorescence spectra of **Acr•** in MeCN solution at different concentrations, with excitation by light of different wavelengths: Excitation and emission spectra at **a)** 25 μM concentration and **b)** 250 μM concentration; **c)** excitation spectra following emission at 490 nm at different concentrations; and **d)** emission spectra following excitation at 420 nm at different concentrations. The abbreviation “arb. u.” is used to refer to the arbitrary units of the fluorescence measurements.

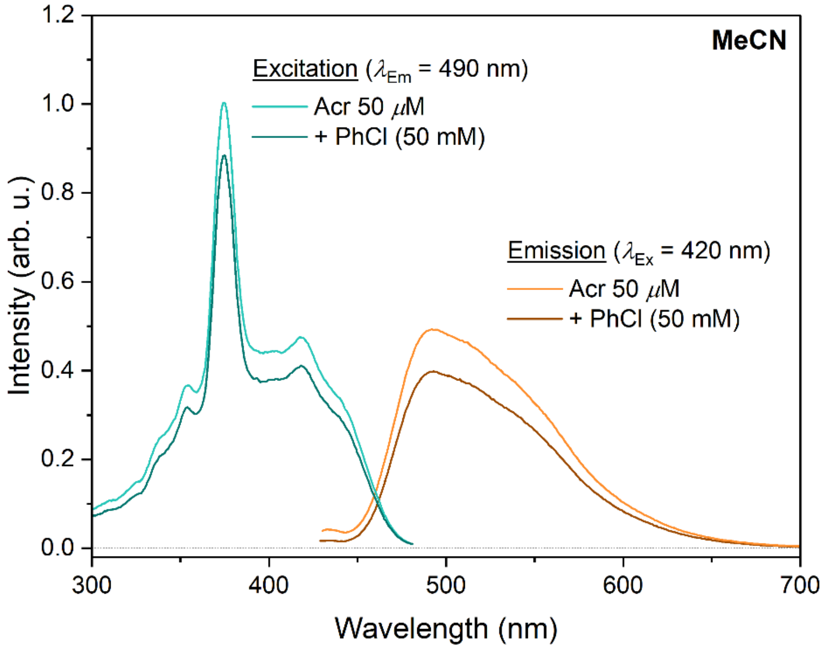

Figure S52: Fluorescence emission and excitation spectra of **Acr•** (50 μM) in MeCN solution and in the presence of chlorobenzene (50 mM). The abbreviation "arb. u." is used to refer to the arbitrary units of the fluorescence measurements.

5.2 Fluorescence lifetime analysis

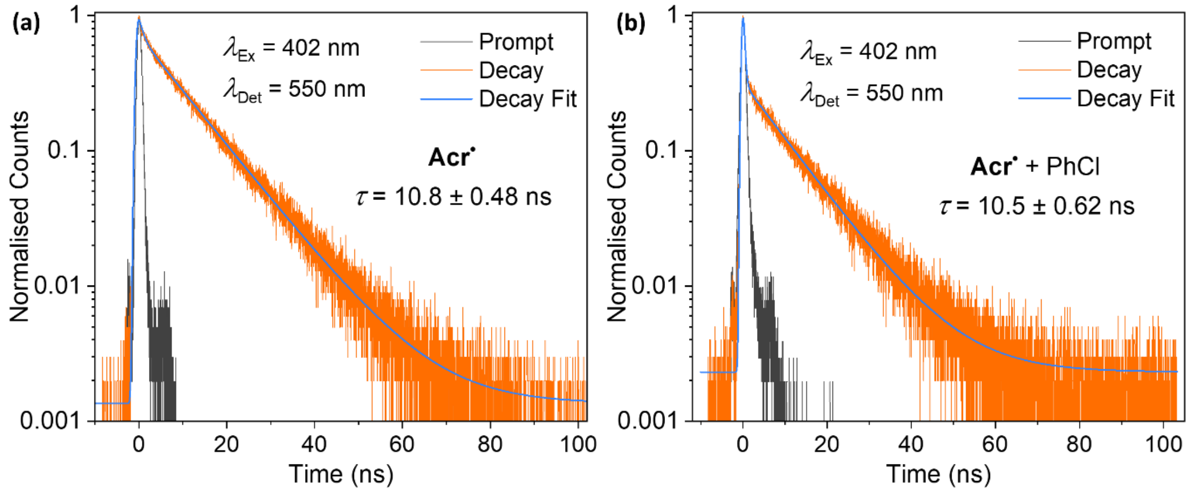

Figure S53: Fluorescence decay traces and fits for MeCN solutions of (a) **Acr•** and (b) **Acr•** in the presence of PhCl. Concentrations: **Acr•**, 0.5 mM; PhCl, 50 mM. λ_{Ex} = 402 nm, λ_{Em} = 550 nm.

5.3 Transient absorption spectroscopy

The lifetimes of the various deconvoluted evolution-associated spectra (EAS) components contained within the TA data are shown in Table S2 while the raw (chirp-corrected) plots of the TA data are shown as 2D matrices below (Figure S54-Figure S58).

Visual inspection of these matrices suggests that in some cases transient features with very short lifetimes, < 1 ps, may be present. However, due to the limited number of data points available in the sub-ps window, and the resolution of the data, we have limited our analysis to EAS components with lifetimes > 1 ps. This means that some initial, ultrafast processes may not be captured by our model, such as any immediate, partial relaxations $D_n \rightarrow D_m$ ($n > m \geq 1$), which can often occur on such ultrafast timeframes. This means that individual EAS traces observed experimentally can only be tentatively correlated with the D_n states calculated by TD-DFT (see Section 7, below). For example, based on calculated electronic energies and oscillator strengths, photoexcitation at 530 nm is expected to primarily populate the D_2 state. However, the "*[**Acr**$^•$]^{530}" EAS observed at this wavelength could plausibly correspond to either D_2 or D_1, with the latter being generated *via* unresolved, sub-ps relaxation of the former. However, it should be emphasised that the presence (or absence) of these additional, sub-ps transitions does not have any effect on the broader mechanistic interpretation of these spectra (i.e. reactivity occurring faster than the diffusion limit).

In all experiments, the TA data (> 1 ps) are well modelled as either monoexponential processes with a single EAS component, or as sequential biexponential processes with two EAS components. Residual plots show no evidence for significant, "unassigned" bleaches or absorptions (though see Section 6.2 for additional discussion). However, as discussed in the main manuscript, close visual inspection of the data for 395 nm irradiation of **Acr**$^•$ in MeCN in the presence of PhCl shows that one of the calculated EAS components in fact corresponds to two separate transients. In this case, the data quality does not allow for adequate deconvolution of these two transients from each other and from the other EAS component. As such, the lifetimes calculated for both EAS components should only be considered qualitatively accurate in this case.

In principle, a similar phenomenon would provide an alternative explanation for the almost identical shapes and lifetimes of the EAS components currently assigned to *[**Acr**$^•$]^{530} and *[**Acr**$^•$]^{395}. As discussed in the main manuscript, these have been assigned as separate states due to their divergent behaviour in the presence of PhCl, with a reduced lifetime and evolution into a new EAS component, *[**Acr**$^+$·PhCl$^{•-}$], observed only for *[**Acr**$^•$]^{395}. However, if these are not in fact different states (e.g. if they are both the result of sub-ps relaxation to D_1, as is common), this would imply that *[**Acr**$^+$·PhCl$^{•-}$] is instead generated from a third transient, which cannot be adequately deconvoluted from *[**Acr**$^•$]^{395} and *[**Acr**$^+$·PhCl$^{•-}$]. However, so far visual inspection of the TA data has not provided any direct evidence to support this alternative explanation. (Note that this alternative explanation would also still lead to the same overall mechanistic conclusions regarding preassembly.)

Table S2: Calculated lifetimes of the EAS components from TA. Concentrations: **Acr•**, 1 mM; chlorobenzene (PhCl), 50 mM; 4-chlorobenzonitrile (4-ClBzN), 50 mM. * indicates a component with a very short lifetime ($\tau \leq$ 1 ps), not discussed in the text due to low data quality and certainty at this timescale. † indicates a very minor long-lived component (see Section 6.2). Note that lifetimes approaching or exceeding the timescale of the experiment (2500 ps) should not be considered precise.

Experiment	Solvent	λ_{Ex} (nm)	EAS	τ (ps)	2σ (ps)	k (ps^{-1})	σ (ps^{-1})
**Acr•**	MeCN	395	EAS1	30.1	0.44	0.0332	2.48×10^{-4}
			EAS2	>>2500 ps		1×10^{-4}	0.999
**Acr•**	MeCN	530	EAS1*	0.43	0.0084	2.33	0.023
			EAS2	40.3	0.27	0.0248	8.42×10^{-5}
			EAS3†	>>2500 ps		1×10^{-4}	0.999
**Acr•** + PhCl	MeCN	395	EAS1	29.5	0.64	0.0340	3.70×10^{-4}
			EAS2	2109	79	4.74×10^{-4}	8.90×10^{-6}
**Acr•** + PhCl	MeCN	530	EAS1*	0.38	0.0055	2.62	0.019
			EAS2	37.3	0.24	0.0268	8.77×10^{-5}
			EAS3†	>>2500 ps		1×10^{-4}	0.999
**Acr•** + 4ClBzN	MeCN	530	EAS1	21.7	0.30	0.0460	3.20×10^{-4}
			EAS2†	>>2500 ps		1×10^{-4}	0.999
**Acr•**	THF	395	EAS1	45.0	0.46	0.0222	1.15×10^{-4}
**Acr•**	THF	530	EAS1*	1.13	0.029	0.879	0.0112
			EAS2	42.5	0.17	0.0236	4.76×10^{-5}
**Acr•** + PhCl	THF	395	EAS1	40.4	0.60	0.0248	1.85×10^{-4}
			EAS2	>2500 ps		2.22×10^{-4}	9.40×10^{-6}
**Acr•** + PhCl	THF	530	EAS1*	0.61	0.16	1.633	0.0216
			EAS2	41.4	0.16	0.0242	6.67×10^{-5}
**Acr•**	9 MeCN : 1 THF	395	EAS1	37.0	0.36	0.0270	1.33×10^{-4}
			EAS2	1794	13	5.57×10^{-4}	2.01×10^{-6}
**Acr•**	1 MeCN : 1 THF	395	EAS1	42.7	0.38	0.0234	1.04×10^{-4}
			EAS2	>>2500 ps		1×10^{-4}	0.999
**Acr•**	1 MeCN : 9 THF	395	EAS1	35.7	0.25	0.0280	9.70×10^{-5}

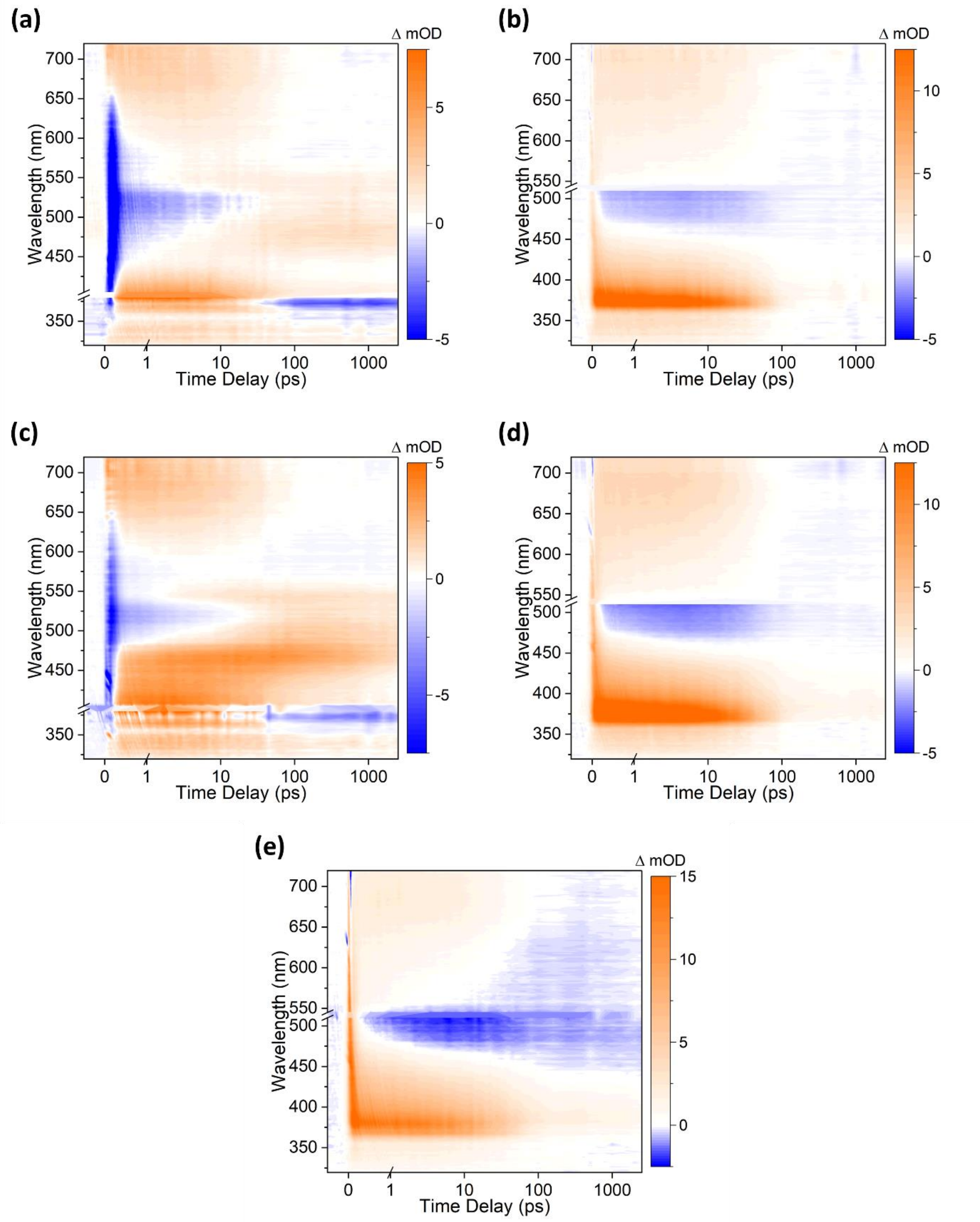

Figure S54: Contour plots of TA spectra for **Acr•** (1 mM) in MeCN solution with: (a) 395 nm excitation; (b) 530 nm excitation; (c) 395 nm excitation with chlorobenzene (50 mM); (d) 530 nm excitation with chlorobenzene; (e) 530 nm excitation with 4-chlorobenzonitrile (50 mM). Time Delay is plotted linearly up to 1 ps, then on a log scale >1 ps.

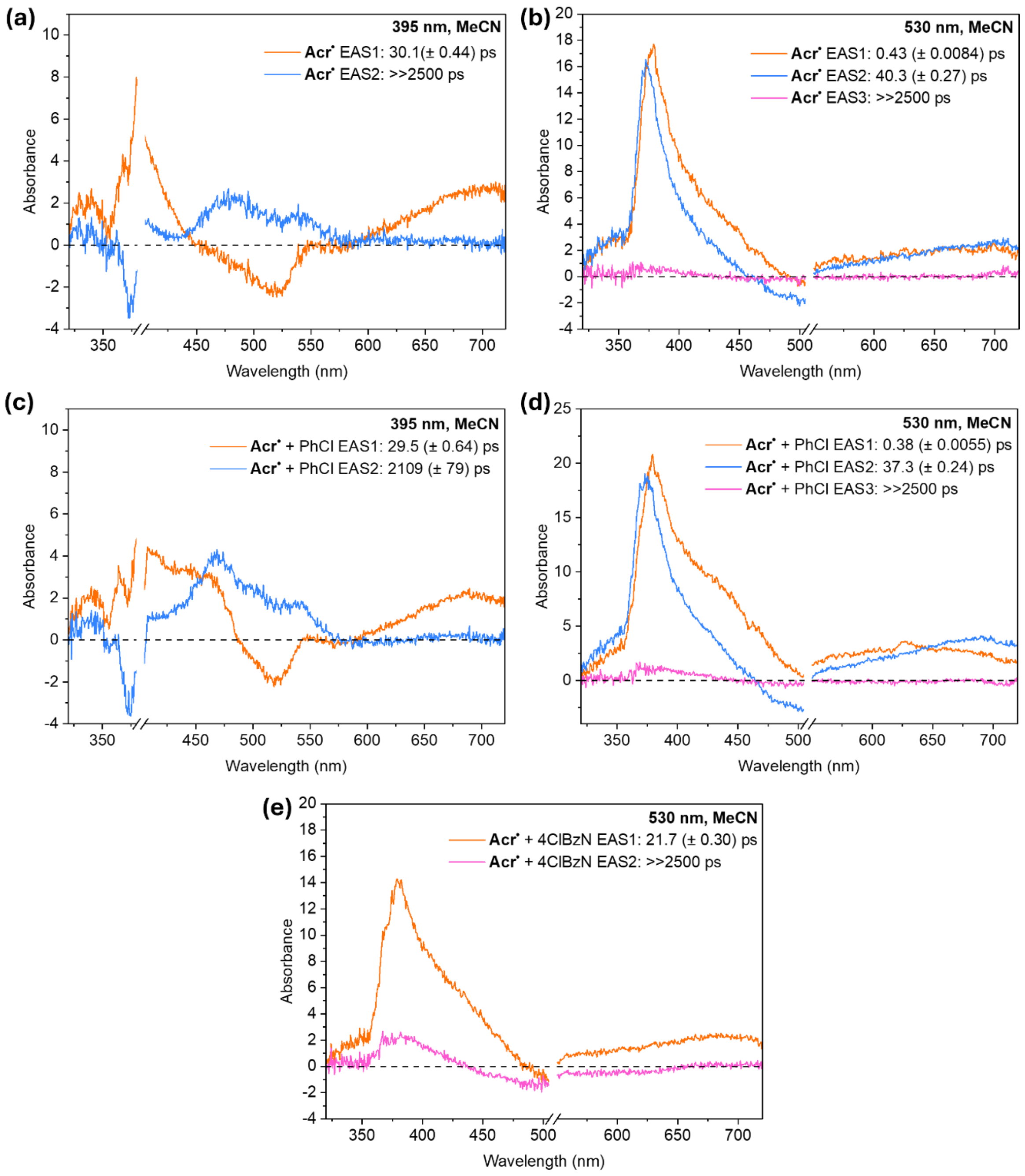

Figure S55: EAS spectra for Acr• (1 mM) in MeCN solution with: (a) 395 nm excitation; (b) 530 nm excitation; (c) 395 nm excitation with chlorobenzene (50 mM); (d) 530 nm excitation with chlorobenzene; (e) 530 nm excitation with 4-chlorobenzonitrile (50 mM).

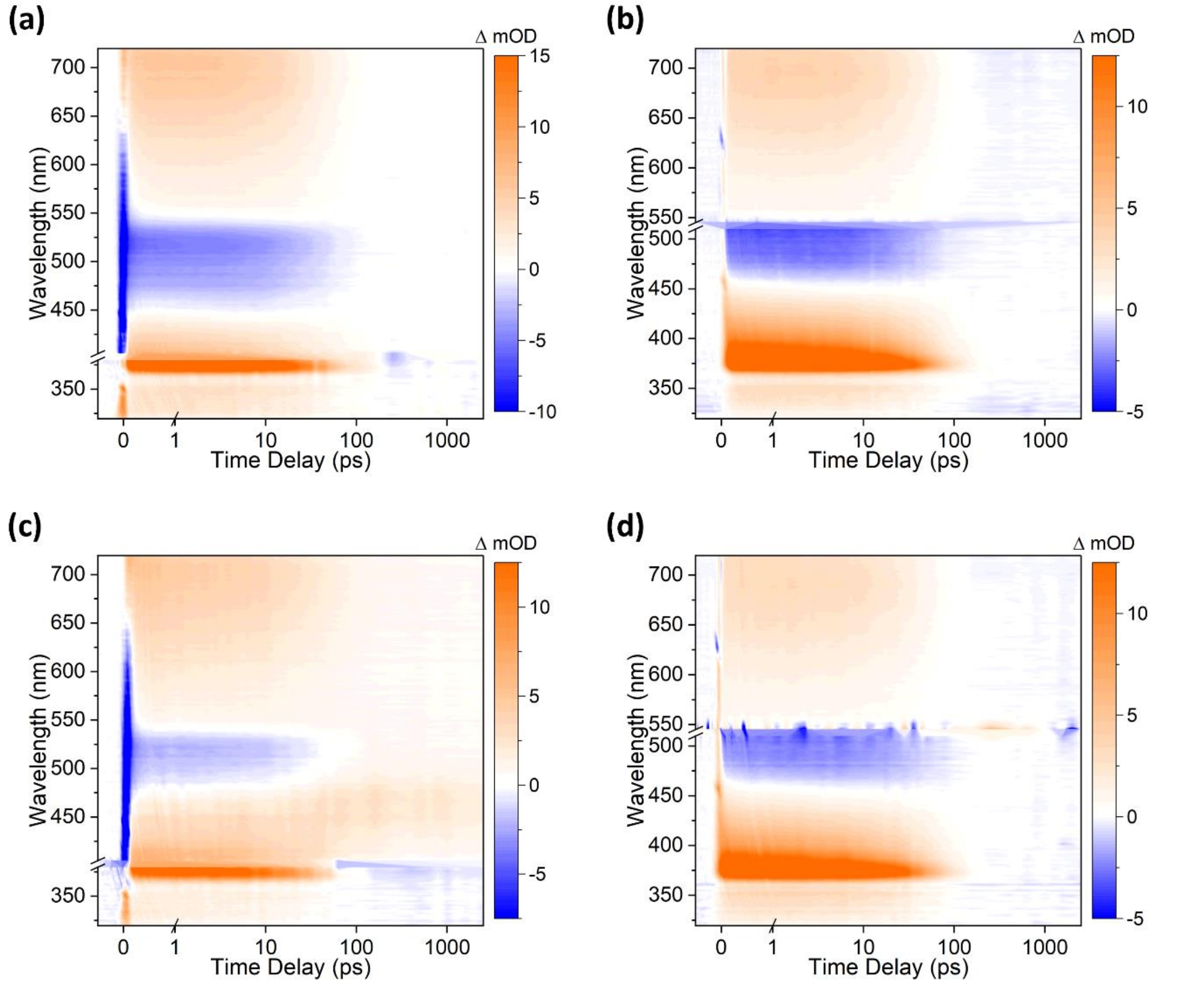

Figure S56: Contour plots of TA spectra for **Acr•** (1 mM) in THF solution with: (a) 395 nm excitation; (b) 530 nm excitation; (c) 395 nm excitation with chlorobenzene (50 mM); (d) 530 nm excitation with chlorobenzene. Time Delay is plotted linearly up to 1 ps, then on a log scale >1 ps.

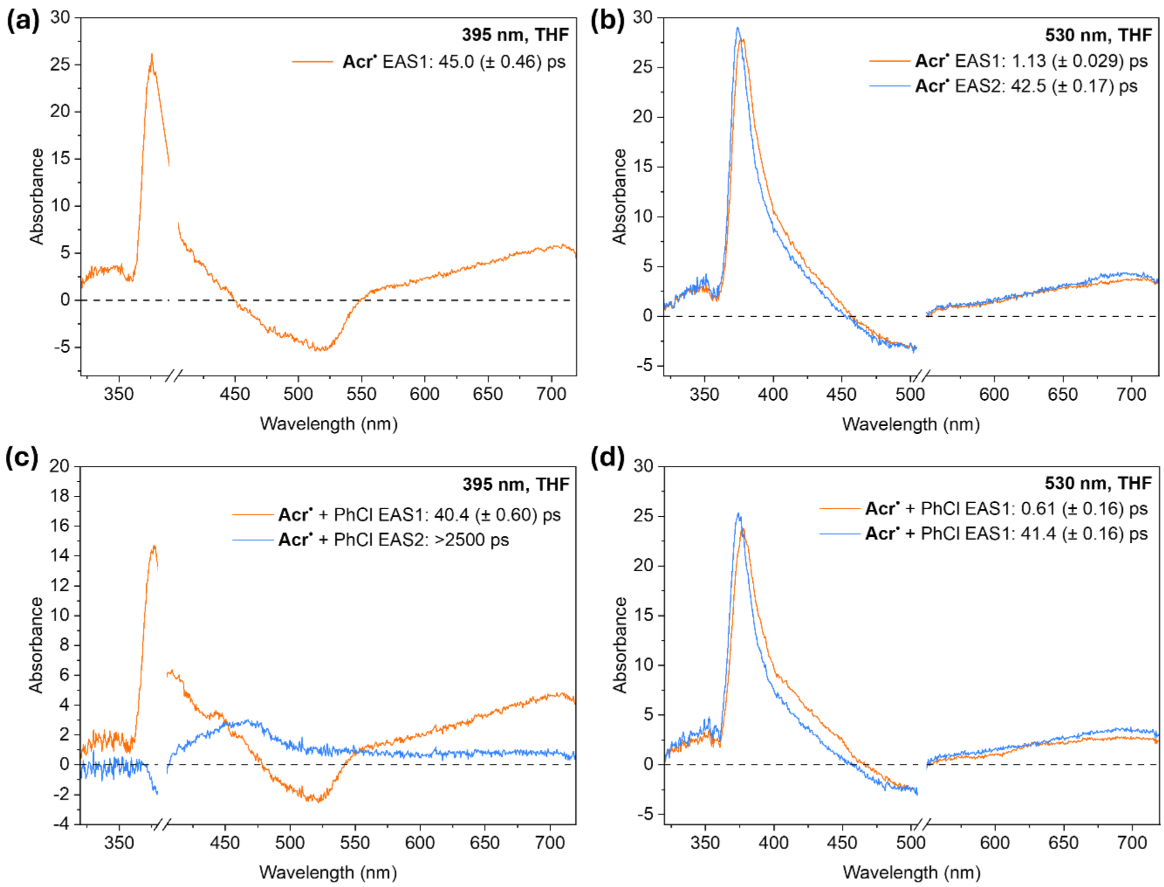

Figure S57: EAS spectra for Acr• (1 mM) in THF solution with: (a) 395 nm excitation; (b) 530 nm excitation; (c) 395 nm excitation with chlorobenzene (50 mM); (d) 530 nm excitation with chlorobenzene.

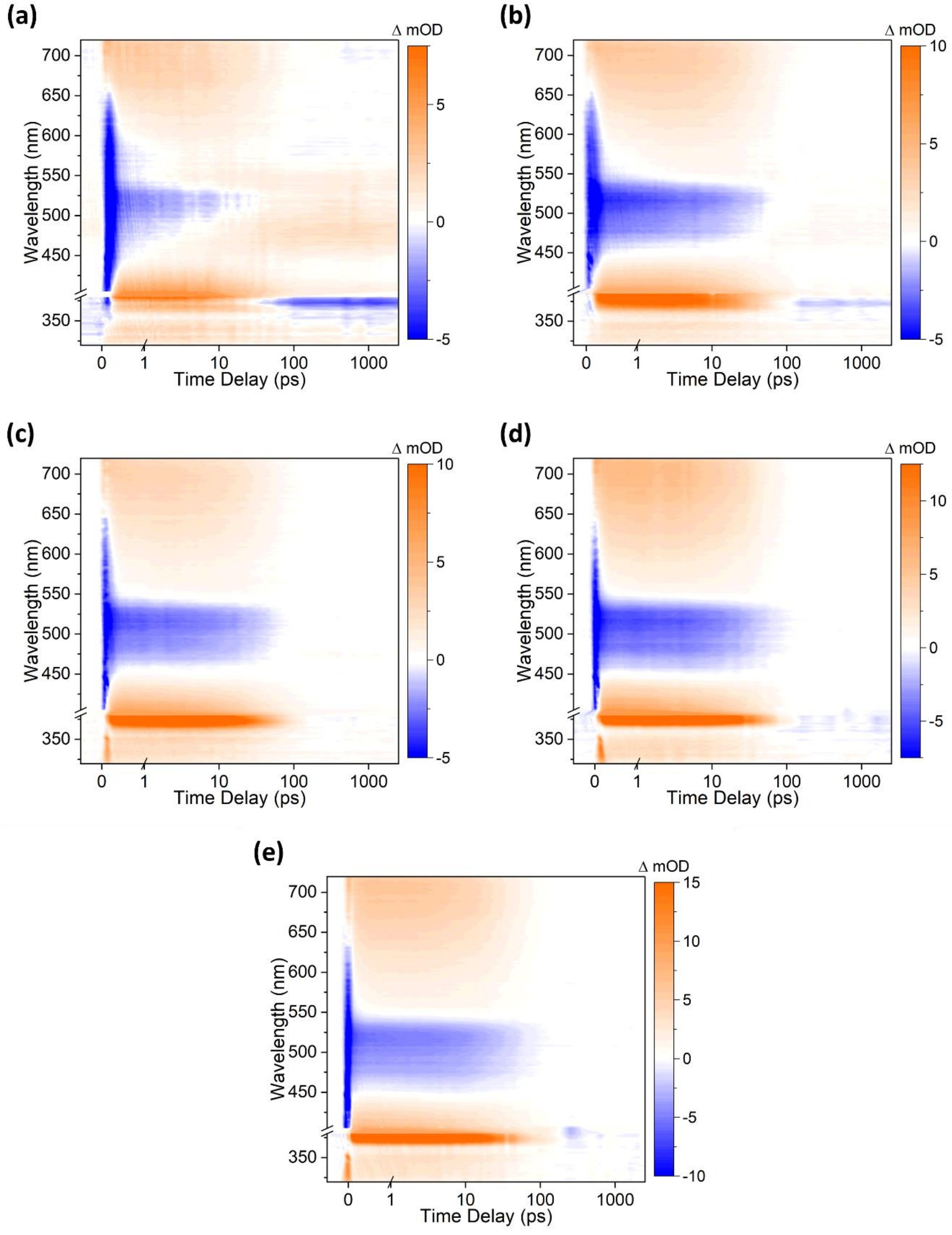

Figure S58: Contour plots of TA spectra for **Acr•** (1 mM) with 395 nm excitation in MeCN:THF in: (a) 100% MeCN; (b) 90% MeCN with 10% THF; (c) 50% MeCN and 50% THF; (d) 10% MeCN in 90% THF; (e) 100% THF. Time Delay is plotted linearly up to 1 ps, then on a log scale >1 ps.

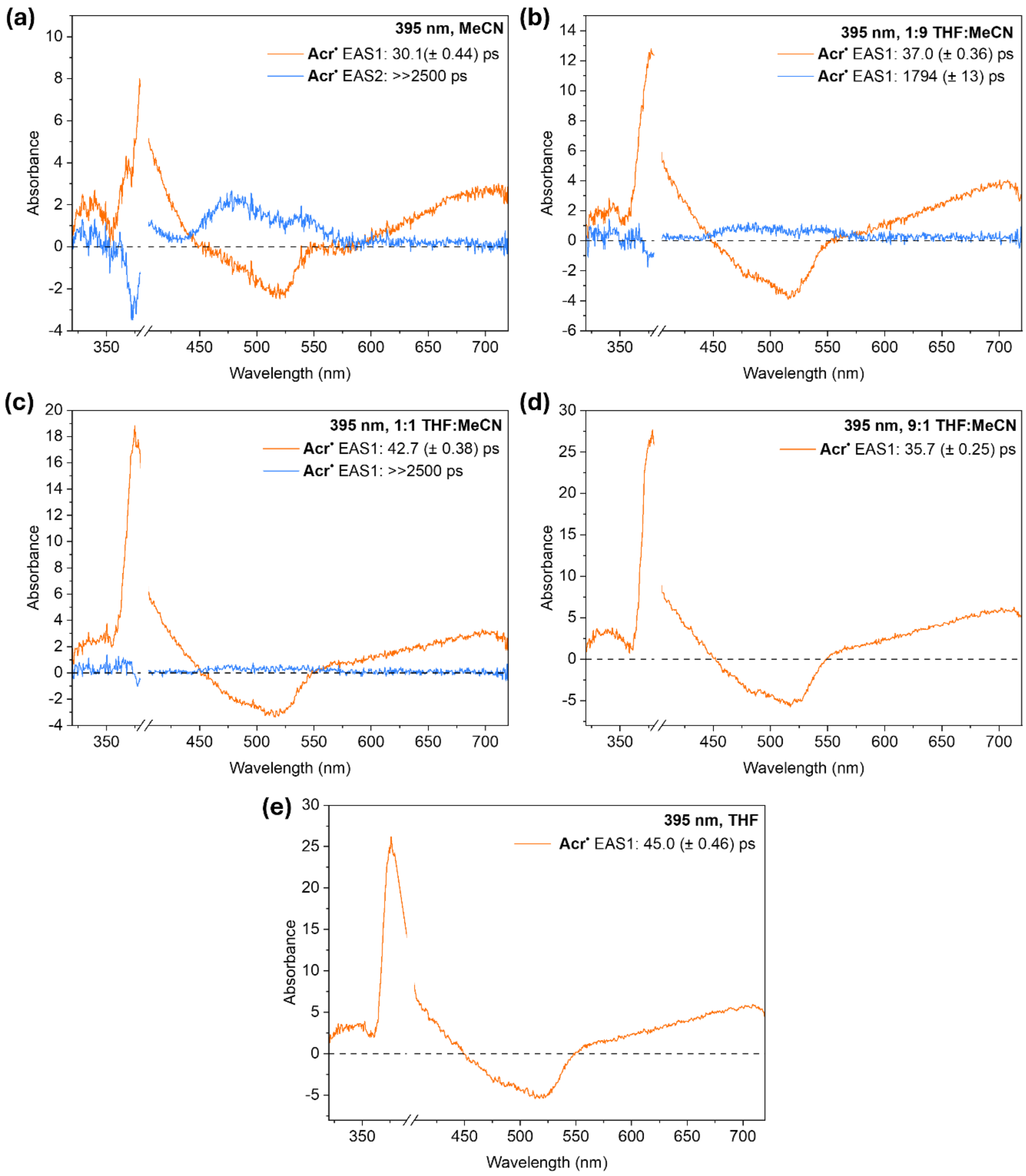

Figure S59: EAS spectra for Acr$^{\bullet}$ (1 mM) with 395 nm excitation in MeCN:THF in: (a) 100% MeCN; (b) 90% MeCN with 10% THF; (c) 50% MeCN and 50% THF; (d) 10% MeCN in 90% THF; (e) 100% THF.

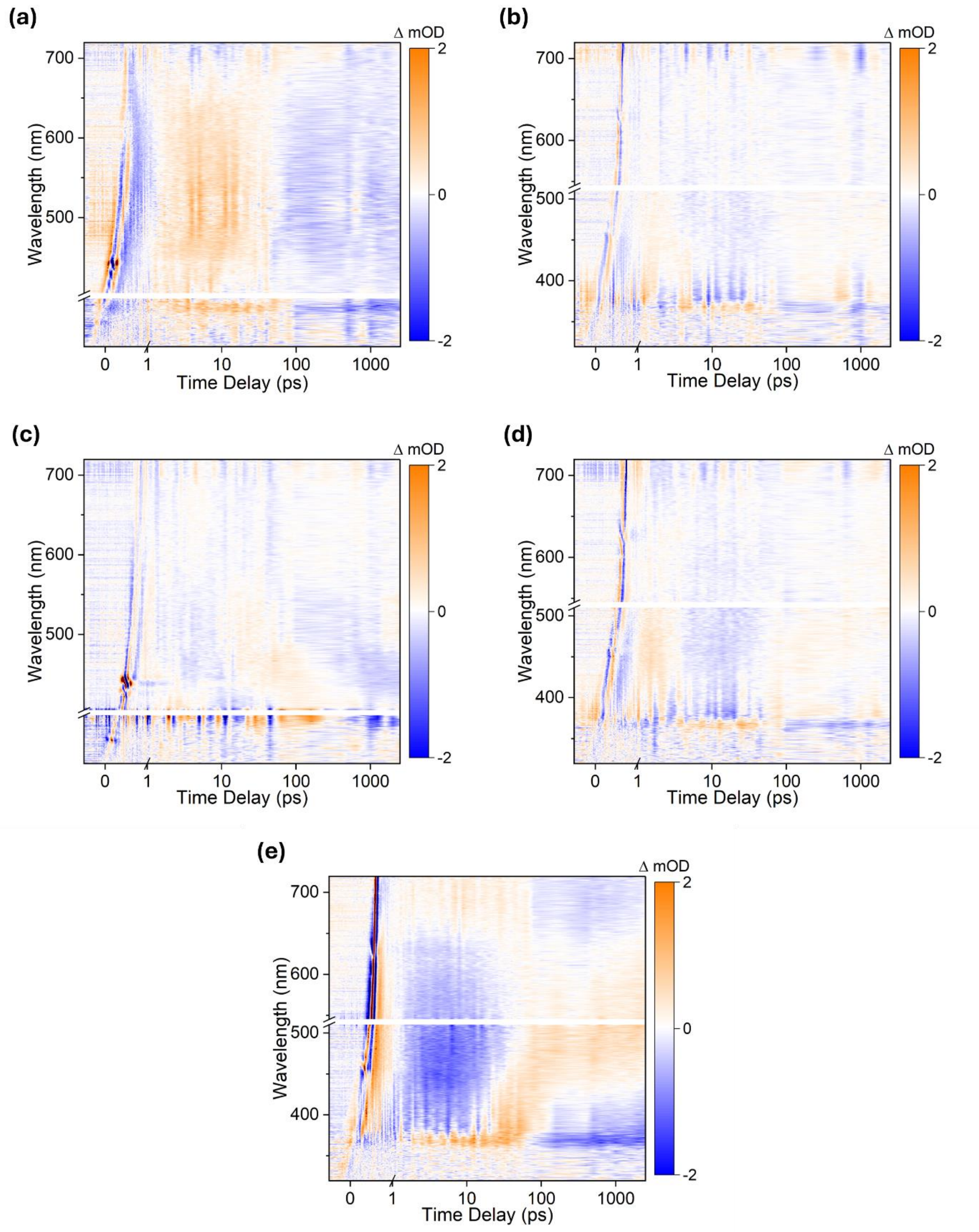

Figure S60: Residual matrices for **Acr•** (1 mM) in MeCN solution under (a) 395 nm excitation; (b) 530 nm excitation; (c) 395 nm excitation with chlorobenzene (50 mM); (d) 530 nm excitation with chlorobenzene; (e) 530 nm excitation with 4-chlorobenzonitrile (50 mM). Time Delay is plotted linearly up to 1 ps, then on a log scale >1 ps.

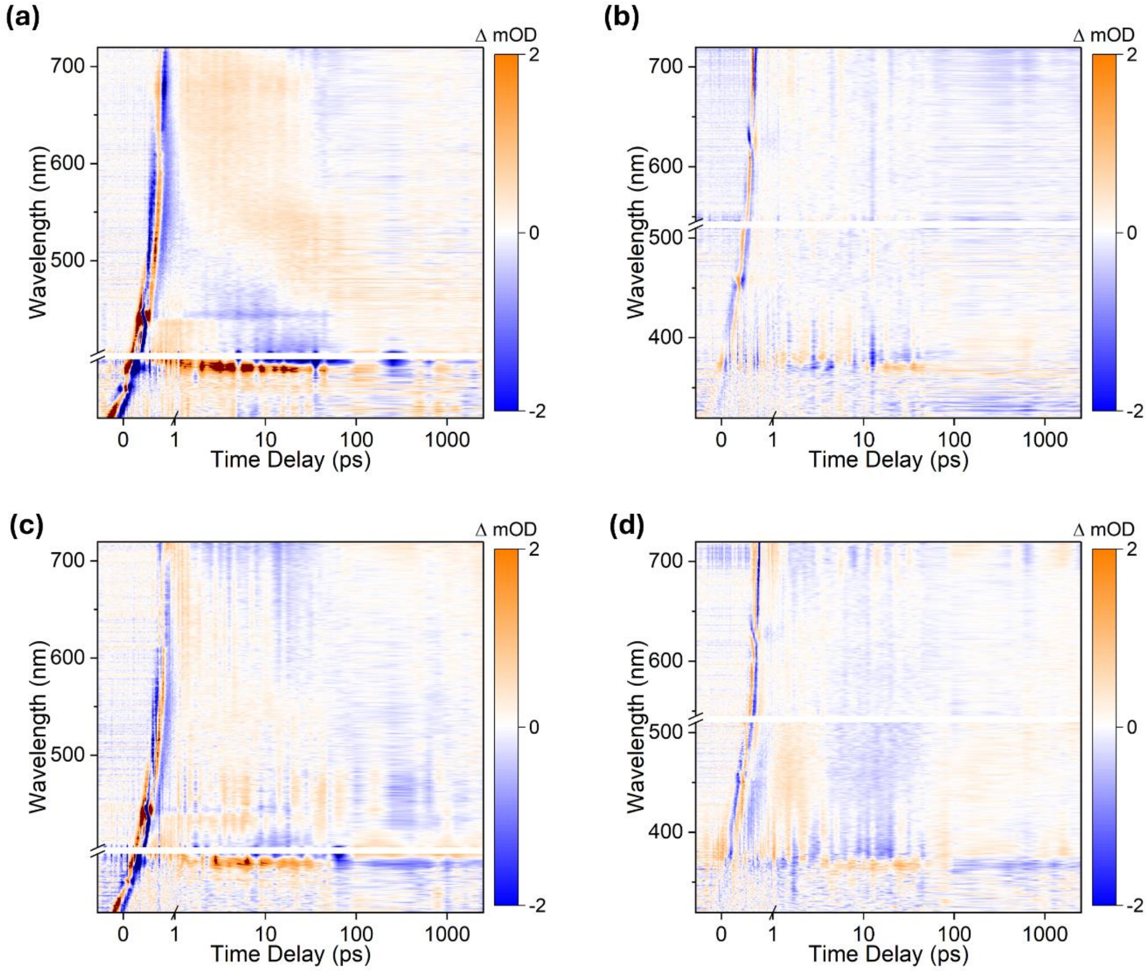

Figure S61: Residual matrices for Acr$^{\bullet}$ (1 mM) in THF solution under (a) 395 nm excitation; (b) 530 nm excitation; (c) 395 nm excitation with chlorobenzene (50 mM); (d) 530 nm excitation with chlorobenzene. Time Delay is plotted linearly up to 1 ps, then on a log scale >1 ps.

6 Additional discussion

6.1 Solvent-dependent Acr$^\bullet$ fluorescence

As discussed in the main manuscript, close inspection of the combined spectroscopic data obtained for the emissive state, *[**Acr**$^\bullet$]^{EM}, allows it to be assigned as the excited state of the oxidised acridinium, *[**Acr**$^+$], which arises *via* transient formation of solvated electrons, $e^-_{(MeCN)}$. This assignment directly accounts for the observed differences in emission between MeCN and THF (which is far less prone to reduction). Nevertheless, for the sake of thoroughness, we have also considered several alternative explanations for the strong solvent dependence of the emissive behaviour observed upon photoexcitation of **Acr**$^\bullet$.

Solvent-dependent emission has been reported in several other contexts to be attributable to differences in aggregation, which can either induce or quench this behaviour.[9] However, so far, we have not observed any evidence for aggregation of **Acr**$^\bullet$ in solution, either in THF or MeCN. In particular, there are no significant differences in the steady-state UV-vis spectra of **Acr**$^\bullet$ in these two solvents (Figure S7), nor are any significant changes detected following changes in concentration (Figure S8) or temperature (down to −35 °C; Figure S9, Figure S12). In the absence of such changes, we currently consider aggregation induced emission/quenching to be unlikely.

We have also considered whether the emission observed in MeCN could occur from a species other than **Acr**$^{\bullet/+}$. Given its spectroscopic purity and stability (including photostability on the timescales required for fluorescence or transient absorption spectroscopy experiments), the presence of an emissive impurity in the isolated **Acr**$^\bullet$ is unlikely. Such an impurity would also itself have to show solvent-dependent emission. Formation of an emissive species due to exposure of **Acr**$^\bullet$ to air during sample preparation (which has led to false assignments during previous studies of other PC$^{\bullet-}$)[10] can be ruled out for similar reasons, as well as the fact that spectroscopic samples were prepared under rigorously air-free conditions inside air-tight cuvettes in well-maintained gloveboxes (see general details in Section 1.1).

Another possibility would be a 'non-innocent' photoreaction of **Acr**$^\bullet$ with the MeCN solvent to transiently generate a new, emissive species *in situ*. Given previous literature discussion of the potential importance of [PC·H]$^-$ states,[11] we have specifically considered the potential for H atom transfer from MeCN to ***Acr**$^\bullet$ to transiently form the closed-shell acridane, **Acr**·H and the MeCN-derived radical [H_2CCN]$^\bullet$ (which would then revert to MeCN and ground-state **Acr**$^\bullet$ *via* back-H atom transfer). DFT calculations suggest that this H atom transfer step would be thermodynamically feasible (see Sections 3.2.1, 8.2). However, this mechanistic pathway should be even easier in THF, which possesses C–H bonds weaker than those in MeCN, suggesting that this is unlikely to account for the observed emission.* We have also found no experimental evidence for the existence of such 'non-innocent' pathways so far (see also Section 3.2). As such, we consider this to also be an unlikely explanation for the observed solvent-dependent fluorescence. (It should also be emphasised that these alternative explanations would not affect the overall mechanistic conclusions of this study, as the emissive state is unreactive.)

*(Assuming H atom transfer *is* easier in THF, it is also possible that, rather than leading to a more emissive [PC·H]$^-$ state, transient H atom transfer actually provides an alternative, non-emissive quenching mechanism for ***Acr**$^\bullet$ in THF.)

6.2 Photoreduction of solvent

In addition to the analysis discussed in Section 5.3, above, and in the main manuscript, close inspection of the TA data obtained under 530 nm irradiation in MeCN suggests the presence of a very minor additional component with a relatively long lifetime (>> 2500 ps; marked with † in Table S2). Given the very low intensity (pink traces in Figure S55) we do not expect this to contribute significantly to the overall photophysical or photochemical behaviour of **Acr**$^{•}$. However, for the sake of thoroughness, this trace is discussed here.

The profile of this trace is very different to those assigned either to the long-lived emissive state that follows irradiation at 395 nm (*[**Acr**$^{•}$]^{EM} = *[**Acr**$^{+}$]) or to the similarly long-lived charge-separated complex that forms in the presence of substrate (*[**Acr**$^{+}$·PhCl$^{•-}$]), as it features none of the excited-state absorptions around *ca.* 450-550 nm. Instead, it features an excited state absorption at 380 nm and a bleach at 450-550 nm. While the intensity of these components is very low, making definitive assignment difficult, these features could be consistent with oxidation of **Acr**$^{•}$ to **Acr**$^{+}$, which has a stronger absorption at 370 nm but no absorption above 500 nm.

It is therefore possible that this contribution is due to minor, transient, photoinduced oxidation of **Acr**$^{•}$. In the absence of substrate the most likely explanation for this behaviour is transient photoreduction of the MeCN solvent (and eventual subsequent charge recombination), directly analogous to what is proposed to occur at shorter wavelengths (e.g. 395 nm), but occurring at 530 nm to only a much lesser degree. This is supported by two observations. Firstly, no such component is observed in THF, which is expected to be more challenging to reduce than MeCN. Secondly, this component appears to be qualitatively more intense in the presence of 4-chlorobenzonitrile, which is known from stoichiometric reactivity studies to be reduced under 530 nm irradiation (producing initially **Acr**$^{+}$ and [ArCl]$^{•-}$), but no more intense in the presence of chlorobenzene (which is not reduced under these conditions). These observations lend further weight to the broader conclusion that transient solvent reduction can provide a secondary pathway for ArCl reduction in MeCN.

Note that we have not been able to observe a similar component under 395 nm irradiation, even though it would be expected if the above hypothesis is correct. However, under these conditions another long-lived species is observed (*[**Acr**$^{•}$]^{EM} = *[**Acr**$^{+}$]) that has a much higher relative intensity and similar lifetime, and it is therefore likely that the new transient simply cannot be deconvoluted from this stronger signal.

6.3 Estimation of collision rate and quenching efficiency

To assess the validity of the 1 ns 'rule of thumb' for diffusion-limited excited state reactivity, we have estimated the lifetime for diffusive encounters between **Acr•** and PhCl under the conditions used for the reactivity studies described in Section 3. From Smoluchowski and Stokes-Einstein we can predict the rate constant for diffusive encounters, k_D, using the standard equation

$$k_D = \frac{4k_B T}{\beta\eta}\left[2 + \frac{r_1}{r_2} + \frac{r_2}{r_1}\right]$$

where r_1 and r_2 are the hydrodynamic radii of **Acr•** and ArCl, η is the solvent viscosity, and β = 6 assuming ideal diffusion (Stokes diffusion, continuous medium).

Using DFT-calculated cavity volumes of 535 Å^3 and 131 Å^3 for **Acr•** and PhCl, respectively, r_1 and r_2 can be estimated as 4.8 Å and 3.3 Å (modelled as simple spheres). Given also the viscosity of MeCN (3.43 x 10^{-4} N s m^{-2}),[12] this gives

$$k_D = 3.3 \times 10^{-17}\ \mathrm{m^3\ s^{-1}}$$

or, in molar units

$$k_D = 2.0 \times 10^{10}\ \mathrm{M^{-1}\ s^{-1}}$$

Given that [ArCl] = 50 mM, this then gives a normalised rate of collision (k_0 = Rate/[**Acr•**]) of

$$k_0 = k_D[\mathrm{ArCl}] = 1.0 \times 10^9\ \mathrm{s^{-1}}$$

or, expressed as lifetime, τ,

$$\tau = \frac{1}{k_0} = 1.0 \times 10^{-9}\ \mathrm{s} = 1.0\ \mathrm{ns}$$

which is remarkably consistent with the 1 ns rule of thumb. This rate/lifetime can be used to calculate the *maximum* possible quenching efficiency for ***Acr•**, assuming that every collision results in quenching

$$\eta_{\max} = \frac{k_D[\mathrm{ArCl}]}{\frac{1}{\tau_0} + k_D[\mathrm{ArCl}]}$$

where τ_0 refers to the ***Acr•** lifetime in the absence of substrate. Using τ_0 values of *ca.* 40 ps obtained from transient absorption spectroscopy gives

$$\eta_{\max} \approx 4\%$$

although as already noted, this value assumes perfectly efficient quenching for every collision. In practice, this is very unlikely, and so actual diffusion-limited efficiencies would be expected to be on the order of *ca.* 1% (or lower).[13]

By comparison, the experimental quenching efficiencies observed *via* transient absorption spectroscopy, η, can be calculated using the ***Acr•** lifetimes measured in the presence (τ) and absence (τ_0) of ArCl

$$\eta = \frac{\tau_0 - \tau}{\tau_0}$$

and are found to be as high as 46% (lifetime reduced from 40.3 ps to 21.7 ps using 4-chlorobenzonitrile in MeCN). Thus, the maximum quenching efficiency observed experimentally is much higher (by over an order of magnitude) than can reasonably be accounted for by diffusion-limited reactivity.

7 Crystallographic information

7.1 General crystallographic information

Crystallographic data were collected at 150 K on a SuperNova diffractometer at the University of Bath. The structure was solved and refined in Olex2 using the ShelXL plugin.[14,15] CCDC number 2433659 contains the crystallographic data for this structure.

7.2 XRD data for Acr•

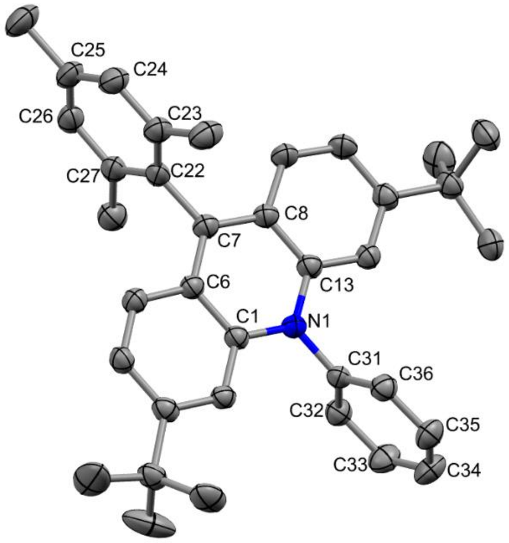

Figure S62: Solid-state molecular structure of **Acr•** as crystallised from a 1:1 mixture of pentane and hexane. Ellipsoids drawn at 50% probability. H atoms, half a hexane molecule, and positional disorder in one tert-butyl group omitted for clarity. Atom colours: C, grey; N, blue.

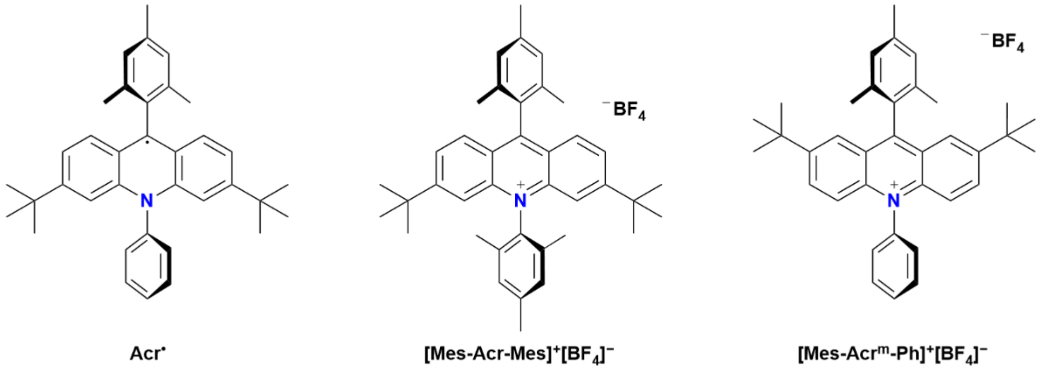

Figure S63: Structures of **Acr•**, **[Mes-Acr-Mes]+[BF4]−**, and **[Mes-Acrm-Ph]+[BF4]−** used for structural data comparisons.

Table S3: Selected structural data for **Acr•**, **[Mes-Acr-Mes]+[BF4]−**,[16] and **[Mes-Acrm-Ph]+[BF4]−**.[17] Ring twist angles are between planes calculated using the mean of six atoms. Parameters for **Acr•** highlighted in **red** feature significant differences from the other structures.

	Structure		
**Structural parameter**	**Acr•**	**[Mes-Acr-Mes]+[BF4]−**	**[Mes-Acrm-Ph]+[BF4]−**
**N1-C1, N1-C13**	**1.397(2)-1.401(2)**	1.373(5)-1.383(5)	1.363(5)-1.365(5)
**C1-C6, C8-C13**	1.418(2)-1.423(2)	1.417(5)-1.431(5)	1.411(5)-1.424(5)
**C6-C7, C7-C8**	1.418(2)-1.420(2)	1.395(5)-1.410(6)	1.400(4)-1.412(5)
***C*-Ar-Acr <**	**88.3°**	76.4°-81.1°	74.9°
***N*-Ar-Acr <**	87.1°	86.7°-89.4°	89.2°

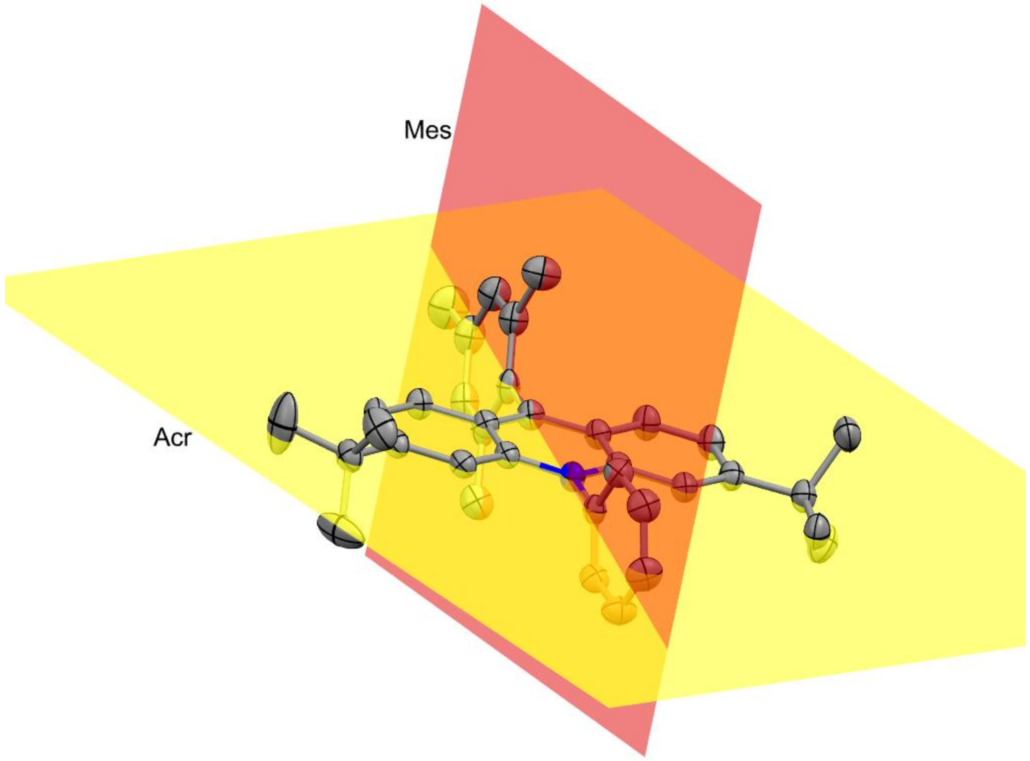

Figure S64: The intersection of the planes calculated from the mean of the Mesityl (Mes) and central acridine (Acr) rings. Interplanar angle = 88.3°.

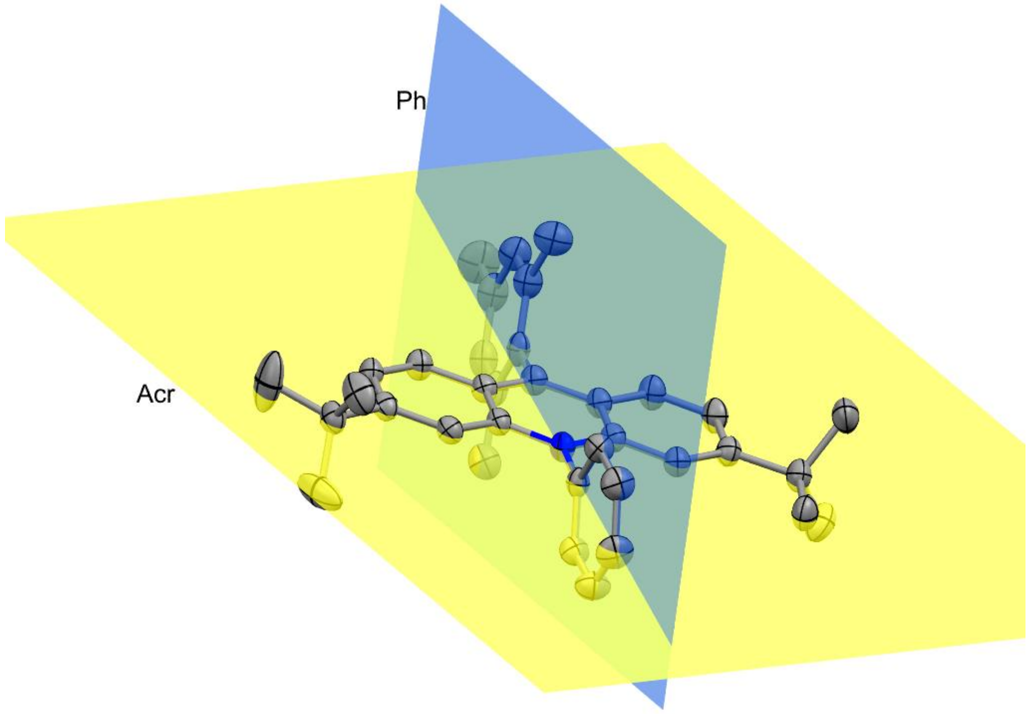

Figure S65: The intersection of the planes calculated from the mean of the central acridine (Acr) and phenyl (Ph) rings. Interplanar angle = 87.1°.

Table S4: Crystallographic collection data and structure refinement details for **Acr**$^{\bullet}$·$(C_6H_{14})_{0.5}$.

**Empirical formula**	$C_{39}H_{47}N$
**Formula weight**	529.815
**Temperature/K**	150.00(10)
**Crystal system**	triclinic
**Space group**	P-1
**a/Å**	10.0665(4)
**b/Å**	11.4895(5)
**c/Å**	14.6052(6)
**α/°**	99.373(4)
**β/°**	101.903(3)
**γ/°**	91.385(3)
**Volume/Å^3**	1627.91(12)
**Z**	2
**ρ_{calc}g/cm^3**	1.081
**μ/mm^{-1}**	0.456
**F(000)**	577.6
**Crystal size/mm^3**	0.15 × 0.15 × 0.1
**Radiation**	Cu Kα (λ = 1.54184)
**2Θ range for data collection/°**	7.82 to 145.88
**Index ranges**	-9 ≤ h ≤ 12, -14 ≤ k ≤ 14, -16 ≤ l ≤ 18
**Reflections collected**	10907
**Independent reflections**	6335 [R_{int} = 0.0305, R_{sigma} = 0.0519]
**Data/restraints/parameters**	6335/92/405
**Goodness-of-fit on F^2**	1.061
**Final R indexes [I>=2σ (I)]**	R_1 = 0.0613, wR_2 = 0.1699
**Final R indexes [all data]**	R_1 = 0.0787, wR_2 = 0.1898
**Largest diff. peak/hole / e Å^{-3}**	0.37/-0.25

Table S5: Tabulated bond distances in **Acr**$^{\bullet}$.

**Atom**	**Atom**	**Length/Å**	**Atom**	**Atom**	**Length/Å**
N1	C1	1.398(2)	C14	C17B	1.502(13)
N1	C13	1.401(2)	C18	C19	1.533(3)
N1	C31	1.438(2)	C18	C20	1.538(2)
C1	C2	1.398(2)	C18	C21	1.523(3)
C1	C6	1.423(2)	C22	C23	1.396(3)
C2	C3	1.401(2)	C22	C27	1.405(2)
C3	C4	1.395(2)	C23	C24	1.396(3)
C3	C14	1.536(2)	C23	C28	1.510(3)
C4	C5	1.382(2)	C24	C25	1.393(3)
C5	C6	1.408(2)	C25	C26	1.378(3)
C6	C7	1.420(2)	C25	C29	1.520(3)
C7	C8	1.419(2)	C26	C27	1.391(3)
C7	C22	1.492(2)	C27	C30	1.502(3)
C8	C9	1.413(2)	C31	C32	1.387(2)
C8	C13	1.418(2)	C31	C36	1.386(2)
C9	C10	1.377(2)	C32	C33	1.382(3)
C10	C11	1.401(3)	C33	C34	1.391(3)
C11	C12	1.392(2)	C34	C35	1.382(3)
C11	C18	1.529(2)	C35	C36	1.390(3)
C12	C13	1.397(2)	C37	C38	1.516(5)
C14	C15	1.522(3)	C37B	C38B	1.53(3)
C14	C15B	1.530(13)	C38	C39	1.513(5)
C14	C16	1.542(3)	C38B	C39B	1.49(3)
C14	C16B	1.471(15)	C39	C39[1]	1.513(6)
C14	C17	1.539(4)	C39B	C39B[1]	1.38(5)

[1]1-X,-Y,-Z

Table S6: Tabulated bond angles in **Acr•**.

Atom	Atom	Atom	Angle/°	Atom	Atom	Atom	Angle/°
C13	N1	C1	122.24(14)	C17	C14	C15B	134.4(7)
C31	N1	C1	119.74(13)	C17	C14	C16	106.7(2)
C31	N1	C13	118.01(13)	C17	C14	C16B	85.1(9)
C2	C1	N1	121.02(14)	C17B	C14	C3	105.7(6)
C6	C1	N1	118.67(14)	C17B	C14	C15	79.0(9)
C6	C1	C2	120.31(15)	C17B	C14	C15B	106.6(10)
C3	C2	C1	121.82(15)	C17B	C14	C16	136.9(9)
C4	C3	C2	117.76(14)	C17B	C14	C16B	118.6(13)
C14	C3	C2	119.60(15)	C17B	C14	C17	36.8(9)
C14	C3	C4	122.63(15)	C19	C18	C11	108.84(15)
C5	C4	C3	121.12(15)	C20	C18	C11	109.18(15)
C6	C5	C4	122.25(15)	C20	C18	C19	109.23(17)
C5	C6	C1	116.75(14)	C21	C18	C11	112.63(15)
C7	C6	C1	120.43(15)	C21	C18	C19	108.69(16)
C7	C6	C5	122.82(15)	C21	C18	C20	108.21(17)
C8	C7	C6	119.22(15)	C23	C22	C7	120.45(15)
C22	C7	C6	121.08(14)	C27	C22	C7	119.37(16)
C22	C7	C8	119.69(14)	C27	C22	C23	120.16(16)
C9	C8	C7	122.66(15)	C24	C23	C22	119.28(17)
C13	C8	C7	120.33(15)	C28	C23	C22	120.45(16)
C13	C8	C9	117.00(15)	C28	C23	C24	120.26(18)
C10	C9	C8	121.85(16)	C25	C24	C23	121.14(19)
C11	C10	C9	121.11(15)	C26	C25	C24	118.63(17)
C12	C11	C10	117.90(15)	C29	C25	C24	120.7(2)
C18	C11	C10	119.58(15)	C29	C25	C26	120.67(19)
C18	C11	C12	122.50(16)	C27	C26	C25	122.05(17)
C13	C12	C11	121.85(16)	C26	C27	C22	118.74(18)
C8	C13	N1	118.93(15)	C30	C27	C22	121.17(16)
C12	C13	N1	120.82(15)	C30	C27	C26	120.09(16)
C12	C13	C8	120.25(15)	C32	C31	N1	119.19(14)
C15	C14	C3	112.47(18)	C36	C31	N1	119.53(15)
C15B	C14	C3	107.0(7)	C36	C31	C32	121.20(15)
C15B	C14	C15	27.7(6)	C33	C32	C31	119.52(15)
C16	C14	C3	109.82(16)	C34	C33	C32	119.86(17)
C16	C14	C15	108.2(2)	C35	C34	C33	120.16(17)
C16	C14	C15B	85.8(6)	C36	C35	C34	120.50(16)
C16B	C14	C3	113.2(9)	C35	C36	C31	118.75(16)
C16B	C14	C15	122.6(9)	C39	C38	C37	111.9(3)
C16B	C14	C15B	105.2(10)	C39B	C38B	C37B	111(2)
C16B	C14	C16	22.3(9)	C39[1]	C39	C38	113.7(4)
C17	C14	C3	109.23(17)	C39B[1]	C39B	C38B	123(4)
C17	C14	C15	110.3(3)				

[1]1-X,-Y,-Z

8 Computational Studies

8.1 TD-DFT details

The wavefunction analysis was performed using the Gaussian16 and Multiwfn software packages.[18,19] The geometry optimizations were performed with the empirical dispersion (D3 with BJ damping) corrected B3LYP functional along with the TZVP basis set, and employing the SMD implicit solvent model for acetonitrile and THF.[20-23] The stationary points were confirmed by harmonic frequency calculations, which showed zero imaginary frequencies. The first 30 vertical transitions were calculated using TD-DFT. These calculations also included the effect of implicit solvation via the nonequilibrium linear response formalism. Note that this approach has already been benchmarked and yields spectra that closely match experimental spectra.[24,25] The obtained wavefunctions were used in the hole-electron analysis to calculate the density overlap between hole and electron (S_r index), the distance between centroid of hole and electron (D index), the C_{hole}/C_{ele} functions (used in figures) together with the MO contributions to hole and electron for states below 3.5 eV (above 350 nm).[26]

The ORCA software package was used for the calculation of relaxation rates.[27,28] This requires relaxed geometries in ground and excited states together with their Hessians. The ground state structures of the acridine radical and its PhCl adduct were optimized using the empirical dispersion (D3 with BJ damping) corrected "B3LYP/G" (parametrization used in Gaussian16) functional together with the TZVP basis set employing the RIJCOSX approximation for two-electron integrals. To account for the solvent effects of acetonitrile and THF, the SMD implicit solvent model was used. Conformational analysis (GOAT method) based on XTB energies[29] was used to determine the initial structure of the adduct before DFT optimization. Frequency calculations were employed to obtain their Hessians. The ground state optimized geometries and Hessians were used in the ESD(ABS) module involving the Vertical Gradient (VG) approach to obtain approximate excited state geometries and Hessians.[30] The excited state calculations employed the def2-TZVP basis set with RIJCOSX and the Tamm-Dancoff Approximation (TDA) turned on, while the Herzberg-Teller effect was not included to ensure faster computation.[31,32] The ESD(FLUOR) and ESD(IC) modules were utilized to compute radiative and nonradiative relaxation rates. For the internal conversion, the TDA approximation was turned off and additional nonadiabatic coupling matrix elements (NACMEs) with electron-translation factors were calculated.

The geometry optimised molecular structures of **Acr**$^{\bullet}$, **Acr-H** and **Acr**$^{\bullet}$·PhCl are shown in Figure S66. Note that for simplicity, only the lowest energy adduct structure between **Acr**$^{\bullet}$ and PhCl was considered for energy level calculations. In practice, this system consists of an ensemble of adduct geometries of similar energies, which together comprise "**Acr**$^{\bullet}$·PhCl". The calculated absorption spectra, with comparisons to the experimental spectra are shown in Figure S67 and Figure S68. Full tables containing the DFT-optimised atomic coordinates, and various TD-DFT calculated parameters (k, rate; τ, lifetime; S_r, hole-electron overlap; D index, hole-electron distance; energies; f_{0n}, oscillator strength) can be found in Section 8.2. Selected information is additionally presented in Table S7 and Table S8 for ease of comparison. The molecular orbitals calculated for **Acr**$^{\bullet}$ and **Acr**$^{\bullet}$·PhCl are shown in Figure S69 and Figure S70.

The thermodynamics of **Acr**$^{\bullet}$·PhCl adduct formation and H-atom transfer processes (see section 3.2.1) were estimated from Gibbs free energies computed at B3LYP(D3BJ)/TZVP/SMD level for standard state conditions in solution phase (T = 298.15 K and c = 1 mol/L) using the ideal gas—rigid rotor—harmonic oscillator (RRHO) approximation as implemented in Gaussian16. The optimised geometries of relevant species are reported in Table S16Table S27, and the corresponding total energy data are compiled in Table S28.

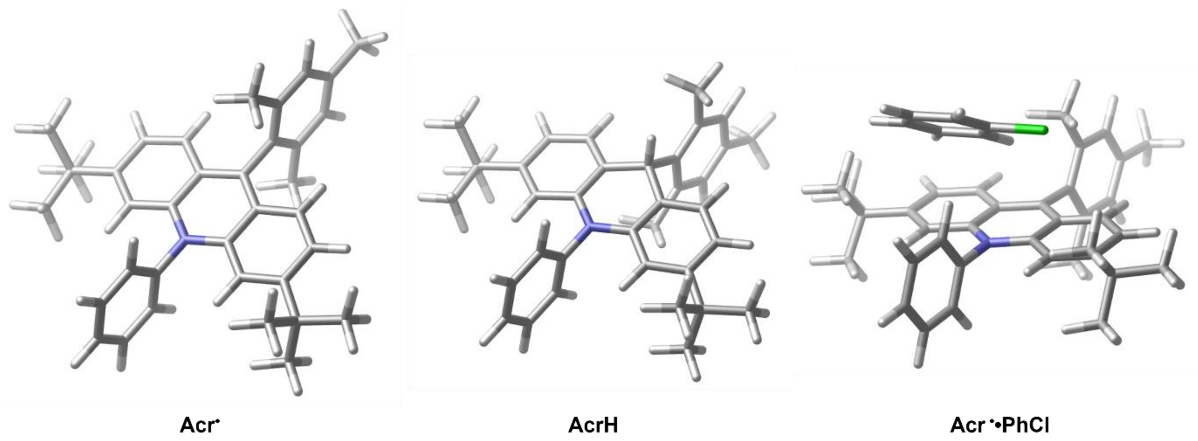

Figure S66: DFT-optimised molecular structures of **Acr•**, **Acr-H** and [**Acr•**·PhCl]

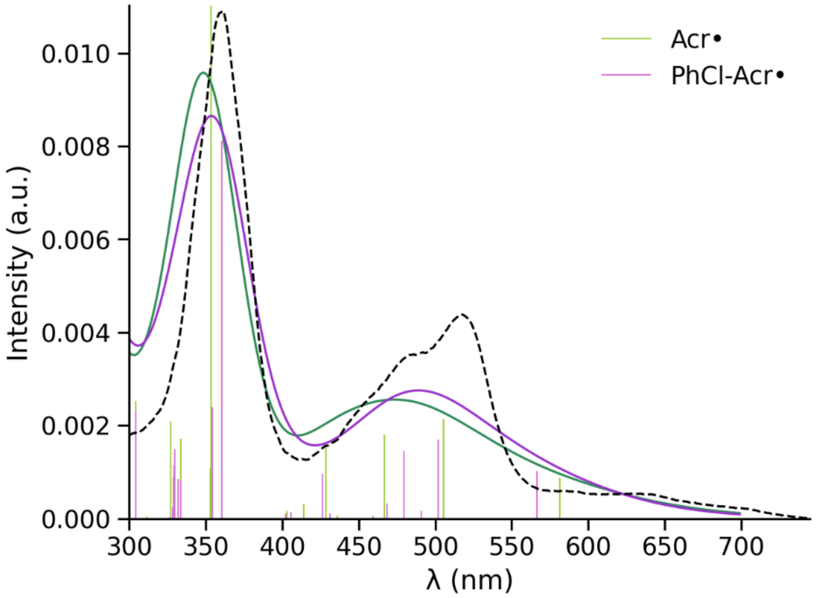

Figure S67: Experimental (dashed line in black) and computed (**Acr•** in green, [**Acr•**·PhCl] in violet) UV-vis spectra in MeCN. The sticks represent the computed vertical excitations with heights scaled by their oscillator strengths. The line spectra are the same vertical excitations but convoluted with Gaussian functions as described in ref. 26 using parameters Δ = 0.2 eV and α = 1.

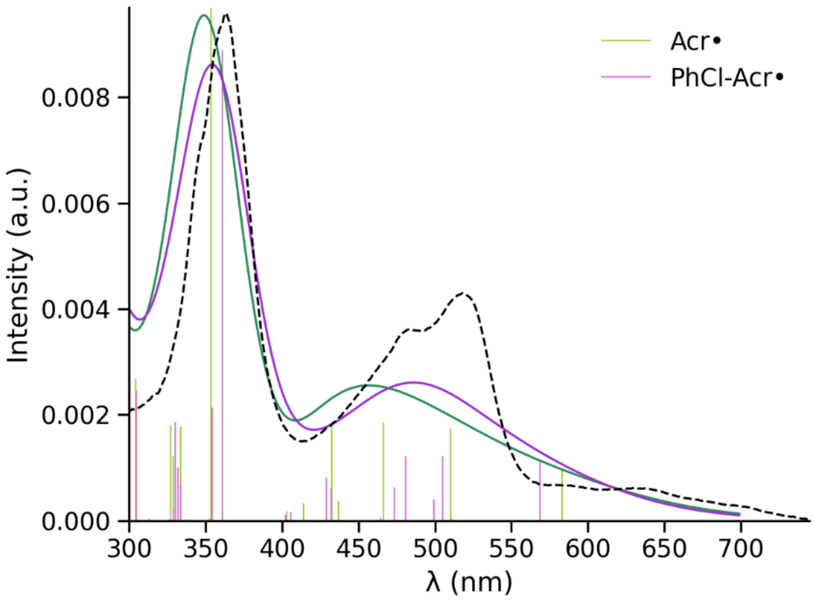

Figure S68: Experimental (dashed line in black) and computed (**Acr**$^{•}$ in green, [**Acr**$^{•}$·PhCl] in violet) UV-vis spectra in THF. The sticks represent the computed vertical excitations with heights scaled by their oscillator strengths. The line spectra are the same vertical excitations but convoluted with Gaussian functions as described in ref. 26 using parameters Δ = 0.2 eV and α = 1.

Table S7: Calculated ES energies and oscillator strengths (f) from TD-DFT in MeCN solution for **Acr•** and for preassembly complex [**Acr•**·PhCl].

Calculated $D_0 \rightarrow D_n$ for Acr•				Calculated $D_0 \rightarrow D_n$ for [**Acr•**·PhCl]			
D_n	ΔE /eV	λ /nm	f_{0n} /10[2]	f_{0n} /10[2]	λ /nm	ΔE /eV	D_n
D_1	2.132	582	1.93	2.26	567	2.188	D_1
D_2	2.453	505	4.73	3.77	502	2.471	D_2
D_3	*2.486*	*499*	*0*	*0.36*	*491*	*2.525*	*D_3*
D_4	2.656	467	3.99	3.24	479	2.586	D_4
-	-	-	-	**0.71**	**469**	**2.646**	**$D_{PhCl,1}$**
-	-	-	-	**0.10**	**459**	**2.699**	**$D_{PhCl,2}$**
D_5	*2.844*	*436*	*0.14*	*0.24*	*431*	*2.876*	*D_5*
D_6	2.891	429	3.57	2.14	426	2.909	D_6
D_7	2.995	414	0.68	0.30	406	3.057	D_7
D_8	*3.076*	*403*	*0.36*	*0.25*	*402*	*3.082*	*D_8*
D_9	3.509	353	25.60	18.04	361	3.438	D_9
D_{10}	*3.511*	*353*	*2.38*	*5.33*	*355*	*3.497*	*D_{10}*

Entries in **bold** denote an intermolecular charge transfer, those in *italics* denote an intramolecular charge transfer. Blank lines included to highlight similar entries across all columns.

Table S8: Calculated ES energies and oscillator strengths (f) from TD-DFT in THF solution for **Acr•** and for preassembly complex [**Acr•**·PhCl].

Calculated $D_0 \rightarrow D_n$ for Acr•				Calculated $D_0 \rightarrow D_n$ for [**Acr•**·PhCl]			
D_n	ΔE /eV	λ /nm	f_{0n} /10[2]	f_{0n} /10[2]	λ /nm	ΔE /eV	D_n
D_1	2.126	583	2.16	2.44	569	2.180	D_1
D_2	*2.427*	*511*	*0.12*	2.71	505	2.456	D_2
D_3	2.429	510	3.85	*0.09*	*499*	*2.483*	*D_3*
D_4	2.658	467	4.10	2.69	481	2.578	D_4
-	-	-	-	**1.41**	**473**	**2.620**	**$D_{PhCl,1}$**
-	-	-	-	**0.12**	**465**	**2.667**	**$D_{PhCl,2}$**
D_5	*2.837*	*437*	*0.81*	1.35	432	2.869	D_5
D_6	2.867	432	3.98	1.78	429	2.890	D_6
D_7	2.995	414	0.72	0.34	406	3.055	D_7
D_8	*3.075*	*403*	*0.37*	*0.24*	*402*	*3.082*	*D_8*
D_9	3.505	354	29.33	19.76	361	3.435	D_9
D_{10}	*3.516*	*353*	*0.06*	*4.74*	*355*	*3.497*	*D_{10}*

Entries in **bold** denote an intermolecular charge transfer, those in *italics* denote an intramolecular charge transfer. Blank lines included to highlight similar entries across all columns.

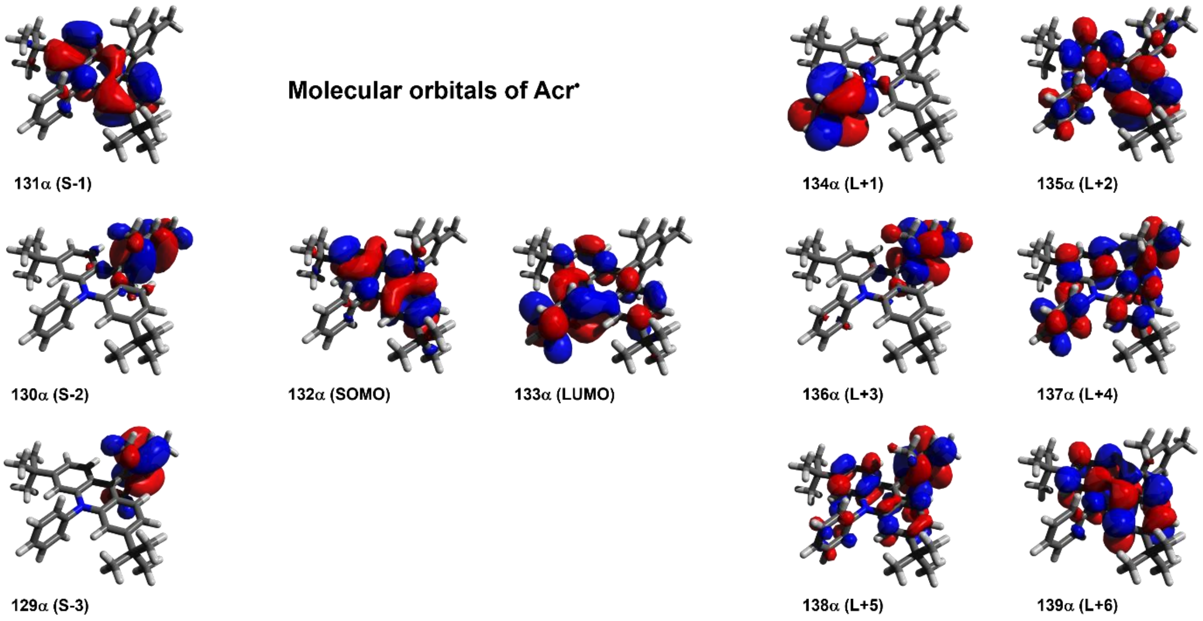

Figure S69: Calculated molecular orbitals of **Acr•**.

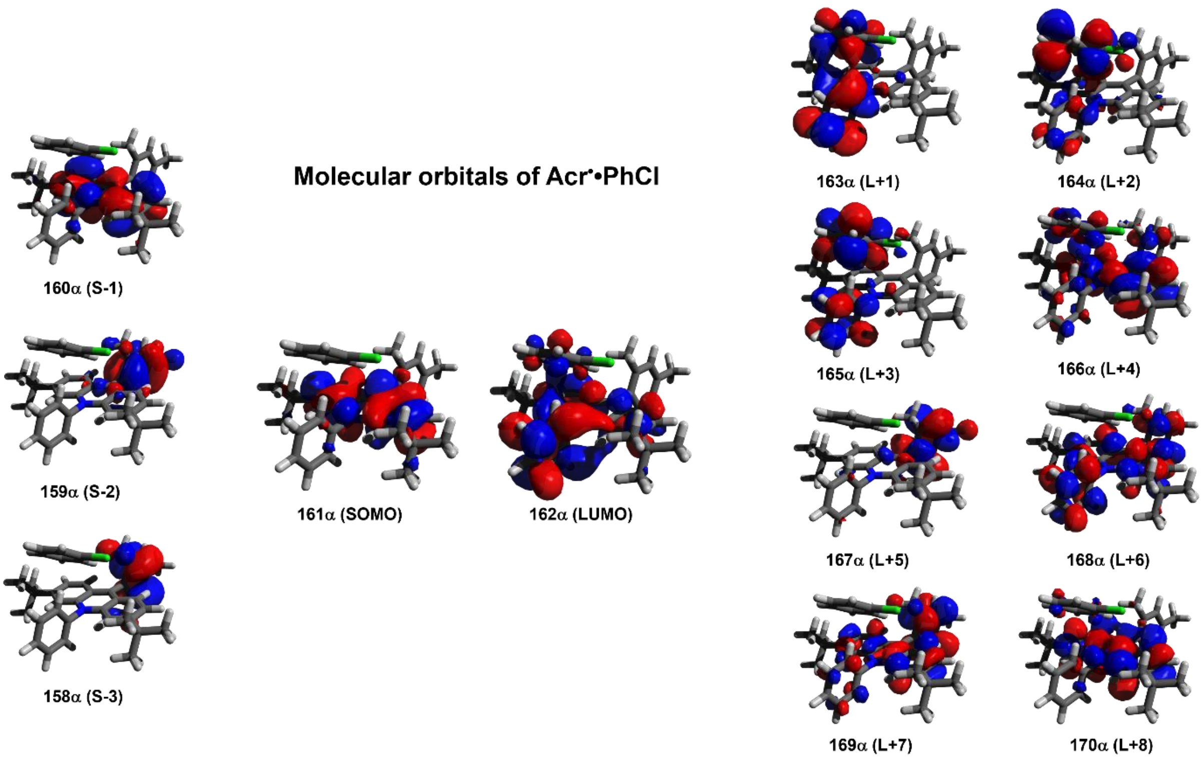

Figure S70: Calculated molecular orbitals of [**Acr•**·PhCl].

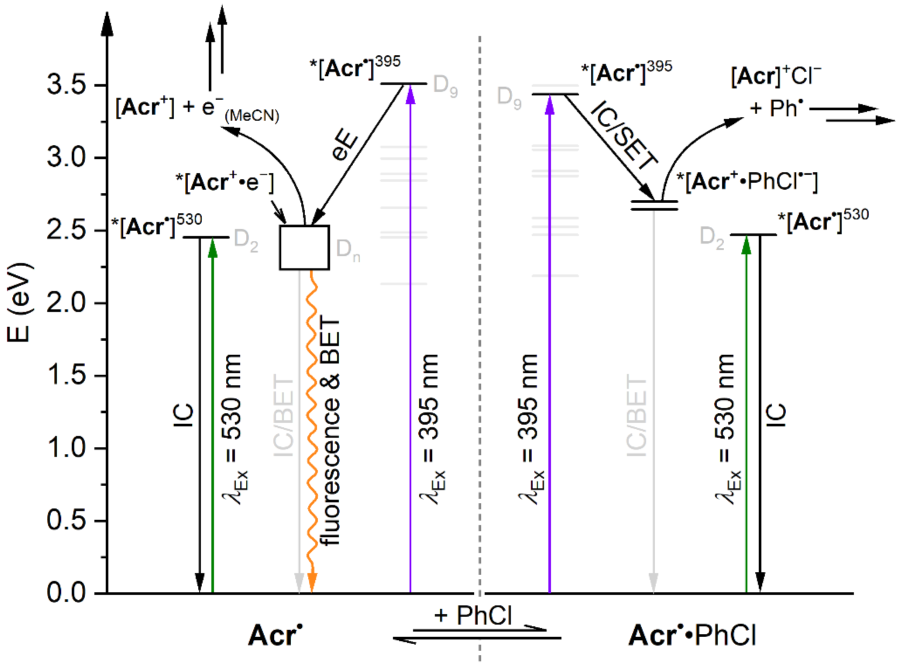

Figure S71: Energy level diagram for Acr• and the hypothesised [**Acr•**·PhCl] encounter complex in MeCN solvent, highlighting the key energy transfer processes undergone following irradiation by either 530 or 395 nm light. Energy levels are from TD-DFT, except for the fluorescence energies which are depicted as a range based on the observed fluorescence. IC = internal conversion; BET = back-electron transfer; eE = electron ejection.

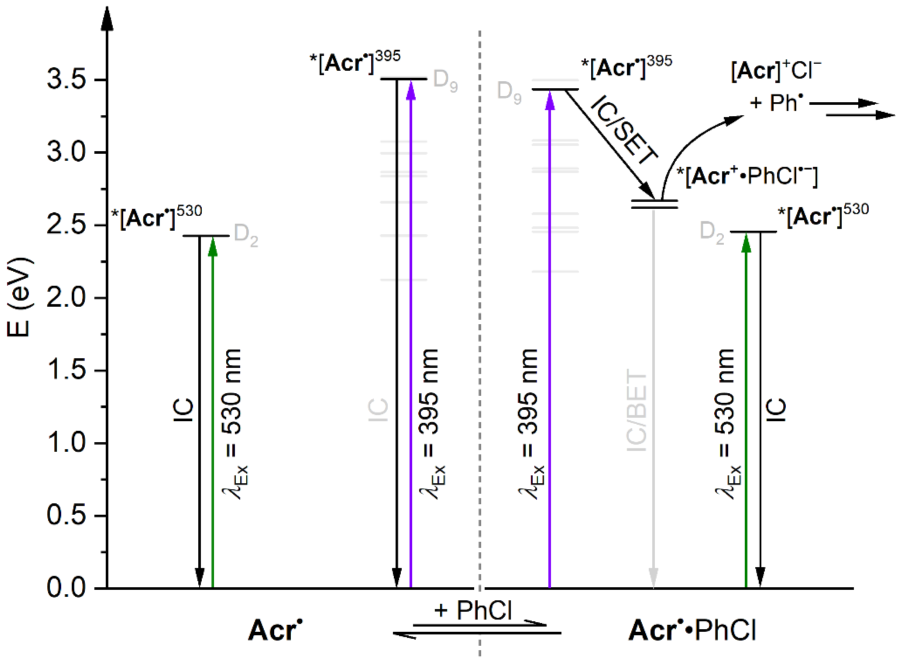

Figure S72: *Reproduced from MS for ease of comparison.* Energy level diagram for Acr• and the hypothesised [**Acr•**·PhCl] encounter complex in THF solvent, highlighting the key energy transfer processes undergone following irradiation by either 530 or 395 nm light. Energy levels are from TD-DFT. IC = internal conversion; BET = back-electron transfer.

8.1.1 Preassembly structures

When determining the lowest energy preassembly structures, hundreds of potential energy minima were calculated. The lowest energy structures calculated in MeCN are typified by the selection shown in Figure S73, which each include some form of π-π interaction (edge-on, slipped face-to-face). The examples shown are not the six lowest in energy but rather a representative selection of the different structures observed, and the majority of the lower-energy structures omitted were rotamers of the pictured structures.

The abundance of π-π interactions in the calculated complexes, as well as preassemblies predicted previously both by DFT[33] and by determination of equilibrium constants,[34] suggests that π-stacking may be a general driving force for preassemblies of this type, and therefore worthy of explicit consideration during reaction design. Stronger non-covalent interactions (such as ionic or H-bonding) would presumably produce an even stronger preference for preassembly, potentially enhancing ultrafast reactivity.

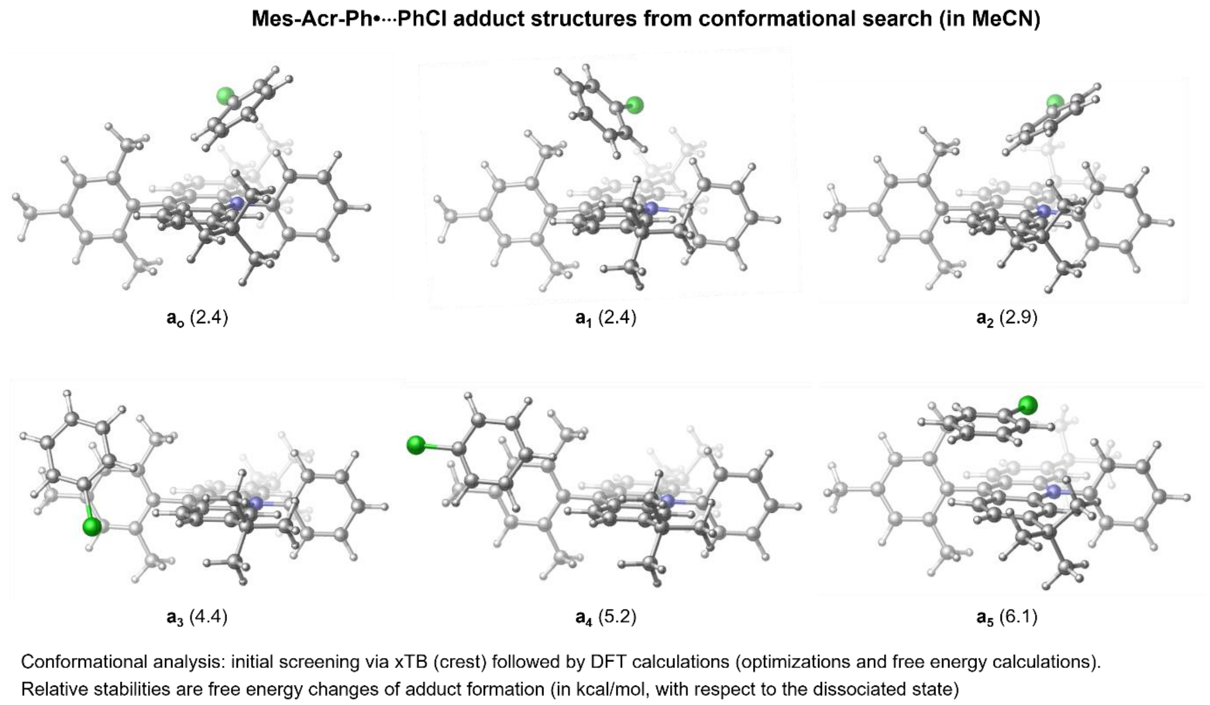

Figure S73: A representative sample of the lowest-energy preassembly structures of **Acr$^{\bullet}$** with PhCl, showing their DFT-calculated free energies of formation in MeCN solvent in kcal mol^{-1} relative to the dissociated state.

8.1.2 Origin of emission from *[Acr⁺]

Photooxidation of **Acr•** to **Acr**$^+$ (*via* loss of $e^-_{(MeCN)}$) could lead to generation of the *[**Acr**$^+$] excited state *via* two possible mechanisms:

either

- secondary photoexcitation of **Acr**$^+$ *after* generation

i.e. $$\mathrm{\mathbf{Acr^{\bullet}}} \xrightarrow{h\nu} {}^*[\mathrm{\mathbf{Acr^{\bullet}}}] \xrightarrow{-\,e^-\,(MeCN)} \mathrm{\mathbf{Acr^{+}}} \xrightarrow{h\nu} {}^*[\mathrm{\mathbf{Acr^{+}}}] \xrightarrow{emission} \mathrm{\mathbf{Acr^{+}}}$$

or

- direct generation *during* photoionisation

i.e. $$\mathrm{\mathbf{Acr^{\bullet}}} \xrightarrow{h\nu} {}^*[\mathrm{\mathbf{Acr^{\bullet}}}] \xrightarrow{-\,e^-\,(MeCN)} {}^*[\mathrm{\mathbf{Acr^{+}}}] \xrightarrow{emission} \mathrm{\mathbf{Acr^{+}}}$$

Chemical intuition suggests that, of these two options, the former is more likely on energetic grounds. This is particularly true for steady-state spectroscopic experiments, where there would be ample time for a steady state concentration of photogenerated **Acr**$^+$ to undergo further photoexcitation.

Nevertheless, the latter possibility cannot be fully excluded, especially during non-steady-state measurements. Although the need for both photoionisation and generation of excited *[**Acr**$^+$] imply a large energetic cost (calculated *[**Acr**$^+$] S_1 energy 2.808 eV, consistent with the experimental emission λ_{max} = 490 nm, 2.53 eV), the overall energy of this process would also be heavily impacted by the nature of any residual Coulombic or other interaction within the *[**Acr**$^+$]/$e^-_{(MeCN)}$ ion pair, making a robust estimate of the wavelength required for photoionisation difficult. It is also noteworthy that, while 395 nm excitation of **Acr•** is expected to initially populate mainly the D_9 state, the D_{10} state is calculated to be essentially identical in energy (calculated λ = 353 nm for both) and only has a *ca.* 10-fold lower calculated oscillator strength in MeCN, suggesting *ca.* 10% initial population of this state, as well. Significantly, D_{10} can be characterised as a charge transfer state (Figure S74), with an electron being excited from the pendant mesityl group to the central acridinyl core. This confirms that 395 nm excitation of **Acr•** can plausibly induce electron transfer from the Mes moiety even to a very poor electron acceptor (in this case, the already reduced acridinyl motif). It also suggests that subsequent loss of an electron to the solvent could plausibly occur from the now doubly reduced acridin<u>ide</u> motif. This would leave an electronic structure consisting of an oxidised Mes moiety and a singly reduced acridinyl moiety; i.e. the *[**Acr**$^+$] charge transfer state (Scheme S1).

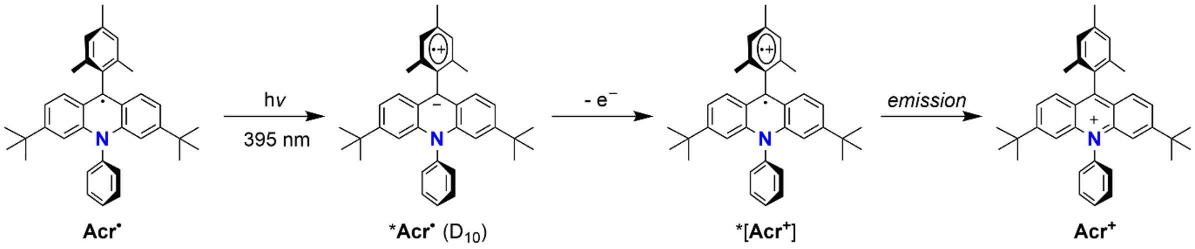

Scheme S1: Possible mechanism for photoionisation of **Acr•** directly to *[**Acr**$^+$] and subsequent emission.

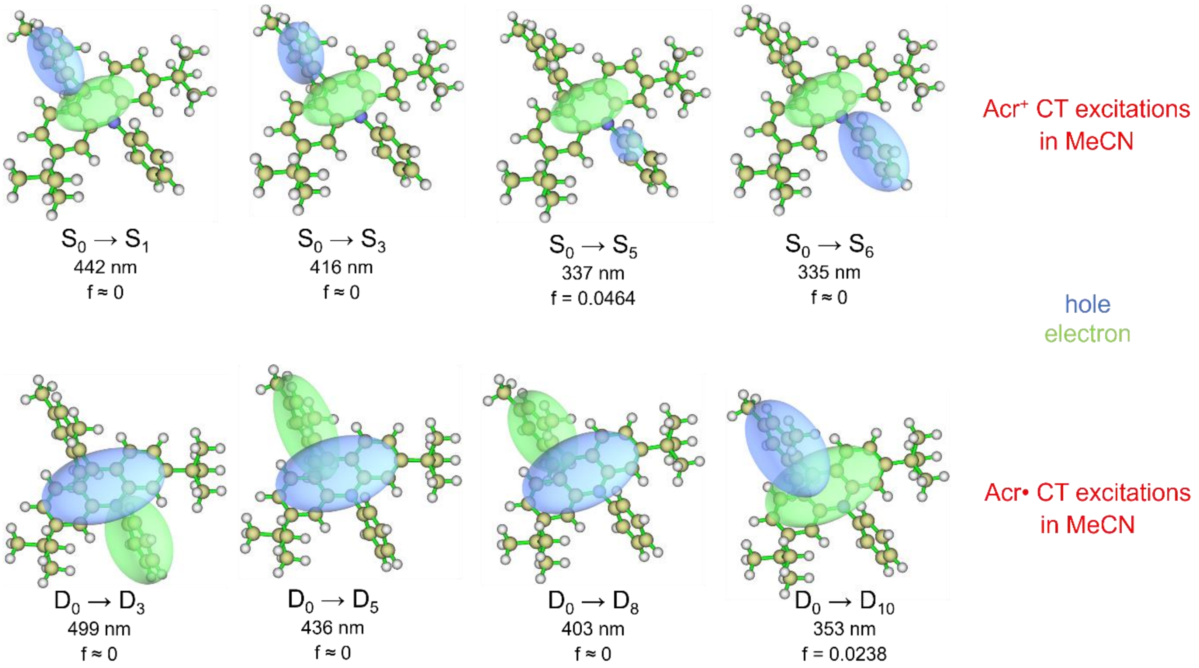

Figure S74: Visual representations of CT excitations of **Acr**$^+$ and **Acr**$^\bullet$.

8.2 TD-DFT information tables

Table S9: TD-DFT calculated parameters for **Acr•** in MeCN solution.

Transition	k_{fluor} (s^{-1})	τ_{fluor} (ps)	k_{IC} (s^{-1})	τ_{IC} (ps)	τ (ps)	S_r (a.u.)	D index (Å)	Hole (MO %)	Electron (MO %)	ΔE (eV)	λ (nm)	f_{0n}
**$D_1 \rightarrow D_0$**	8.68E+06	1.15E+05	1.81E+11	5.54E+00	5.53488	0.69456	0.646	132A (97%)	133A (63%), 135A (31%)	2.132	582	0.0193
**$D_2 \rightarrow D_0$**	2.86E+07	3.50E+04	8.34E+10	1.20E+01	11.98119	0.60724	1.696	132A (98%)	133A (29%), 135A (65%)	2.453	505	0.0473
**$D_3 \rightarrow D_0$**	3.94E+04	2.54E+07	6.98E+08	1.43E+03	1433.081	0.15392	4.115	132A (99%)	134A (100%)	2.486	499	0
**$D_4 \rightarrow D_0$**	3.34E+07	3.00E+04	1.05E+10	9.54E+01	95.1146	0.83159	0.592	132A (7%), 131B (91%)	139A (5%), 132B (91%)	2.656	467	0.0399
**$D_5 \rightarrow D_0$**	8.17E+05	1.22E+06	4.41E+09	2.27E+02	226.943	0.24939	3.532	132A (99%)	136A (97%)	2.844	436	0.0014
**$D_6 \rightarrow D_0$**	5.23E+07	1.91E+04	4.99E+09	2.00E+02	198.3998	0.62788	1.608	132A (91%), 129B (5%)	133A (6%), 137A (76%), 138A (8%), 132B (7%)	2.891	429	0.0357
**$D_7 \rightarrow D_0$**	1.09E+07	9.17E+04	8.73E+10	1.15E+01	11.45838	0.84034	0.411	132A (91%), 131B (5%)	139A (89%), 132B (5%)	2.995	414	0.0068
**$D_8 \rightarrow D_0$**	7.87E+06	1.27E+05	5.32E+09	1.88E+02	187.5171	0.34091	3.524	132A (98%)	137A (12%), 138A (84%)	3.076	403	0.0036
**$D_9 \rightarrow D_0$**	1.97E+07	5.07E+04	6.40E+10	1.56E+01	15.61932	0.86674	0.052	131A (5%), 132A (9%), 127B (4%), 129B (52%), 130B (9%), 131B (5%)	137A (7%), 132B (74%), 135B (5%)	3.509	353	0.256
**$D_{10} \rightarrow D_0$**	5.09E+08	1.96E+03	1.72E+08	5.81E+03	1467.708	0.37279	3.254	129B (6%), 130B (90%)	132B (97%)	3.511	353	0.0238

Table S10: TD-DFT calculated parameters for **Acr+** in MeCN solution.

Transition	S_r (a.u.)	D index (Å)	Hole (MO %)	Electron (MO %)	ΔE (eV)	λ (nm)	f_{0n}
**$S_1 \rightarrow S_0$**	0.1941	3.836	130 (98%)	132 (100%)	2.808	441.54	0
**$S_2 \rightarrow S_0$**	0.77637	0.459	131 (98%)	132 (99%)	2.9589	419.02	0.1012
**$S_3 \rightarrow S_0$**	0.12844	3.93	129 (99%)	132 (100%)	2.9828	415.66	0.0004
**$S_4 \rightarrow S_0$**	0.76617	0.639	128 (88%), 131 (6%)	132 (93%), 133 (6%)	3.5247	351.76	0.4328
**$S_5 \rightarrow S_0$**	0.43927	3.233	127 (95%)	132 (98%)	3.6761	337.27	0.0464
**$S_6 \rightarrow S_0$**	0.15144	4.235	126 (95%)	132 (100%)	3.7051	334.63	0
**$S_7 \rightarrow S_0$**	0.64153	1.063	125 (95%)	132 (96%)	4.0332	307.41	0.0107
**$S_8 \rightarrow S_0$**	0.7673	0.312	124 (89%)	132 (90%), 133 (8%)	4.7633	260.29	0.1498

Table S11: TD-DFT calculated parameters for [**Acr**•·PhCl] in MeCN solution.

Transition	k_{fluor} (s^{-1})	τ_{fluor} (ps)	k_{IC} (s^{-1})	τ_{IC} (ps)	τ (ps)	S_r (a.u.)	D index (Å)	Hole (MO %)	Electron (MO %)	ΔE (eV)	λ (nm)	f_{0n}
**$D_1 \rightarrow D_0$**	1.33E+07	7.52E+04	3.32E+11	3.01E+00	3.011767	0.69508	0.769	161A (96%)	162A (64%), 166A (22%)	2.188	567	0.0226
**$D_2 \rightarrow D_0$**	2.43E+07	4.12E+04	1.09E+11	9.18E+00	9.18015	0.6159	1.533	161A (97%)	162A (24%), 164A (18%), 166A (40%)	2.471	502	0.0377
**$D_3 \rightarrow D_0$**	2.07E+06	4.84E+05	4.39E+09	2.28E+02	227.7034	0.27268	3.811	161A (99%)	163A (81%), 164A (10%)	2.525	491	0.0036
**$D_4 \rightarrow D_0$**	9.11E+06	1.10E+05	3.03E+10	3.30E+01	33.00998	0.84241	0.442	161A (19%), 160B (78%)	164A (8%), 161B (78%)	2.586	479	0.0324
**$D_{PhCl,1} \rightarrow D_0$**	2.88E+07	3.47E+04	1.57E+10	6.38E+01	63.69096	0.52182	3.122	161A (84%), 160B (15%)	163A (7%), 164A (44%), 165A (21%), 161B (15%)	2.646	469	0.0071
**$D_{PhCl,2} \rightarrow D_0$**	5.53E+05	1.81E+06	1.45E+09	6.88E+02	687.8064	0.18375	4.206	161A (100%)	164A (15%), 165A (66%), 166A (15%)	2.699	459	0.001
**$D_5 \rightarrow D_0$**	2.26E+06	4.43E+05	1.20E+10	8.36E+01	83.5901	0.28351	3.271	161A (99%)	167A (97%)	2.876	431	0.0024
**$D_6 \rightarrow D_0$**	3.84E+07	2.60E+04	6.04E+10	1.66E+01	16.55061	0.59999	1.641	161A (91%), 158B (6%)	162A (7%), 168A (76%), 169A (6%), 161B (8%)	2.909	426	0.0214
**$D_7 \rightarrow D_0$**	4.11E+06	2.44E+05	3.44E+10	2.91E+01	29.08231	0.8013	0.394	161A (94%)	170A (87%)	3.057	406	0.003
**$D_8 \rightarrow D_0$**	6.10E+06	1.64E+05	3.56E+09	2.81E+02	280.7144	0.36093	3.342	161A (98%)	168A (10%), 165A (81%)	3.082	402	0.0025
**$D_9 \rightarrow D_0$**	2.90E+08	3.44E+03	5.81E+10	1.72E+01	17.12749	0.81684	0.536	160A (5%), 161A (9%), 158B (29%), 159B (40%), 160B (6%)	168A (7%), 161B (77%)	3.438	361	0.1804
**$D_{10} \rightarrow D_0$**	1.67E+08	5.99E+03	6.54E+09	1.53E+02	149.1233	0.51012	2.678	158B (31%), 159B (57%)	161B (93%)	3.4973	355	0.0533

Table S12: TD-DFT calculated parameters for **Acr•** in THF solution.

Transition	k_{fluor} (s^{-1})	τ_{fluor} (ps)	k_{IC} (s^{-1})	τ_{IC} (ps)	τ (ps)	S_r (a.u.)	D index (Å)	Hole (MO %)	Electron (MO %)	ΔE (eV)	λ (nm)	f_{0n}
**$D_1 \rightarrow D_0$**	8.89E+06	1.12E+05	1.68E+11	5.97E+00	5.965644	0.68576	0.787	132A (97%)	133A (62%), 135A (32%)	2.1258	583	0.0216
**$D_2 \rightarrow D_0$**	2.12E+07	4.72E+04	8.32E+10	1.20E+01	12.01316	0.19372	4.057	132A (99%)	134A (97%)	2.4271	511	0.0012
**$D_3 \rightarrow D_0$**	5.00E+05	2.00E+06	9.39E+08	1.06E+03	1064.19	0.57817	1.911	132A (98%)	133A (30%), 135A (63%)	2.4289	510	0.0385
**$D_4 \rightarrow D_0$**	3.12E+07	3.20E+04	9.84E+09	1.02E+02	101.2564	0.83184	0.588	132A (7%), 131B (90%)	139A (5%), 132B (90%)	2.6578	467	0.041
**$D_5 \rightarrow D_0$**	2.88E+06	3.47E+05	1.73E+10	5.80E+01	57.94378	0.35958	2.764	132A (98%)	136A (90%)	2.8366	437	0.0081
**$D_6 \rightarrow D_0$**	5.66E+07	1.77E+04	1.73E+10	5.77E+01	57.52135	0.62205	0.651	132A (92%)	136A (8%), 137A (74%), 138A (6%), 132B (6%)	2.8668	432	0.0398
**$D_7 \rightarrow D_0$**	1.12E+07	8.92E+04	7.65E+10	1.31E+01	13.07809	0.8431	0.365	132A (91%)	139A (90%), 132B (6%)	2.9947	414	0.0072
**$D_8 \rightarrow D_0$**	6.79E+06	1.47E+05	3.78E+09	2.65E+02	264.1475	0.33195	3.575	132A (98%)	137A (10%), 138A (85%)	3.0745	403	0.0037
**$D_9 \rightarrow D_0$**	8.12E+05	1.23E+06	6.46E+09	1.55E+02	154.6824	0.88903	0.36	131A (6%), 132A (10%), 127B (5%), 129B (59%), 131B (8%)	137A (8%), 132B (73%), 135B (6%)	3.5051	354	0.2933
**$D_{10} \rightarrow D_0$**	5.21E+08	1.92E+03	1.34E+07	7.47E+04	1870.564	0.22649	3.593	130B (99%)	132B (100%)	3.5162	353	0.0006

Table S13: TD-DFT calculated parameters for **Acr+** in THF solution.

Transition	S_r (a.u.)	D index (Å)	Hole (MO %)	Electron (MO %)	ΔE (eV)	λ (nm)	f_{0n}
**$S_1 \rightarrow S_0$**	0.19504	3.859	130 (94%)	132 (100%)	2.7396	452.57	0.0001
**$S_2 \rightarrow S_0$**	0.13346	3.92	129 (99%)	132 (100%)	2.911	425.92	0.0004
**$S_3 \rightarrow S_0$**	0.77237	0.484	131 (94%)	132 (99%)	2.9523	419.95	0.1037
**$S_4 \rightarrow S_0$**	0.76134	0.656	128 (89%), 131 (6%)	132 (93%), 133 (6%)	3.5131	352.92	0.4568
**$S_5 \rightarrow S_0$**	0.43408	3.256	127 (96%)	132 (98%)	3.6686	337.96	0.0475
**$S_6 \rightarrow S_0$**	0.15048	4.234	126 (100%)	132 (100%)	3.6956	335.49	0
**$S_7 \rightarrow S_0$**	0.64059	1.04	125 (95%)	132 (96%)	4.0298	307.67	0.0112
**$S_8 \rightarrow S_0$**	0.21048	4.343	130 (94%)	133 (98%)	4.7181	262.78	0.0008

Table S14: TD-DFT calculated parameters for [**Acr**$^{\bullet}$·PhCl]in THF solution.

Transition	k_{fluor} (s^{-1})	τ_{fluor} (ps)	k_{IC} (s^{-1})	τ_{IC} (ps)	τ (ps)	S_r (a.u.)	D index (Å)	Hole (MO %)	Electron (MO %)	ΔE (eV)	λ (nm)	f_{0n}
**$D_1 \rightarrow D_0$**	1.26E+07	7.91E+04	1.20E+11	8.35E+00	8.351447	0.686	0.903	161A (96%)	162A (65%), 166A (22%)	2.1803	569	0.0244
**$D_2 \rightarrow D_0$**	1.66E+07	6.02E+04	6.88E+10	1.45E+01	14.53088	0.55799	1.979	161A (98%)	162A (23%), 163A (17%), 164A (14%), 165A (8%), 164A (34%)	2.4557	505	0.0271
**$D_3 \rightarrow D_0$**	4.62E+06	2.16E+05	3.89E+09	2.57E+02	256.7991	0.33905	3.538	161A (99%)	163A (74%), 164A (13%), 166A (10%)	2.4831	499	0.009
**$D_4 \rightarrow D_0$**	5.15E+06	1.94E+05	5.08E+09	1.97E+02	196.6374	0.81636	0.973	161A (38%), 160B (60%)	164A (19%), 165A (8%), 166A (6%), 161B (60%)	2.5779	481	0.0269
**$D_{PhCl,1} \rightarrow D_0$**	3.23E+07	3.10E+04	1.34E+10	7.47E+01	74.52593	0.6528	2.447	161A (65%), 160B (34%)	164A (36%), 165A (19%), 166A (5%), 161B (34%)	2.6202	473	0.0141
**$D_{PhCl,2} \rightarrow D_0$**	3.20E+06	3.13E+05	1.49E+09	6.71E+02	669.4085	0.21984	4.135	161A (99%)	164A (14%), 165A (63%), 166A (19%)	2.6673	465	0.0012
**$D_5 \rightarrow D_0$**	4.87E+06	2.05E+05	2.53E+10	3.96E+01	39.57444	0.46282	1.343	161A (96%)	167A (76%), 168A (13%)	2.8693	432	0.0135
**$D_6 \rightarrow D_0$**	3.87E+07	2.59E+04	3.72E+10	2.69E+01	26.88745	0.53629	0.489	161A (94%)	167A (22%), 168A (64%), 161B (5%)	2.8898	429	0.0178
**$D_7 \rightarrow D_0$**	4.13E+06	2.42E+05	3.32E+10	3.01E+01	30.11203	0.81272	0.493	161A (94%)	170A (92%)	3.0551	406	0.0034
**$D_8 \rightarrow D_0$**	5.26E+06	1.90E+05	1.03E+09	9.72E+02	967.0608	0.32872	3.567	161A (98%)	168A (9%), 169A (86%)	3.0815	402	0.0024
**$D_9 \rightarrow D_0$**	1.76E+08	5.69E+03	2.16E+10	4.64E+01	45.9827	0.83185	0.402	160A (5%), 161A (10%), 158B (32%), 159B (36%), 160B (6%)	168A (8%), 161B (76%)	3.4346	361	0.1976
**$D_{10} \rightarrow D_0$**	2.69E+08	3.72E+03	3.97E+09	2.52E+02	236.1288	0.49278	2.808	158B (28%), 159B (61%)	161B (94%)	3.4969	355	0.0474

Table S15: The first 30 vertical transitions for **Acr$^{\bullet}$** and for the [**Acr$^{\bullet}$**·PhCl] preassembly complex as calculated by TD-DFT in MeCN or THF solution. Lifetime predictions were not carried out for transitions higher than the 10th (**Acr$^{\bullet}$**) or 12th ([**Acr$^{\bullet}$**·PhCl]).

**Acr$^{\bullet}$ in MeCN**			**[Acr$^{\bullet}$·PhCl] in MeCN**			**Acr$^{\bullet}$ in THF**			**[Acr$^{\bullet}$·PhCl] in THF**		
Wavelength (nm)	f	S**2	Wavelength (nm)	f	S**2	Wavelength (nm)	f	S**2	Wavelength (nm)	f	S**2
581.5	0.0193	0.825	566.64	0.0226	0.825	583.23	0.0216	0.823	568.65	0.0244	0.823
505.54	0.0473	0.812	501.8	0.0377	0.817	510.83	0.0012	0.808	504.88	0.0271	0.817
498.65	0	0.807	491.13	0.0036	0.81	510.45	0.0385	0.814	499.3	0.009	0.811
466.78	0.0399	0.817	479.38	0.0324	0.815	466.5	0.041	0.817	480.96	0.0269	0.81
436.03	0.0014	0.778	468.64	0.0071	0.805	437.08	0.0081	0.783	473.19	0.0141	0.81
428.81	0.0357	0.808	459.41	0.001	0.796	432.48	0.0398	0.803	464.84	0.0012	0.797
414	0.0068	0.846	431.05	0.0024	0.78	414.02	0.0072	0.846	432.11	0.0135	0.792
403.04	0.0036	0.782	426.25	0.0214	0.812	403.26	0.0037	0.782	429.04	0.0178	0.799
353.31	0.256	1.035	405.64	0.003	0.836	353.72	0.2933	1.056	405.83	0.0034	0.839
353.17	0.0238	0.801	402.34	0.0025	0.785	352.61	6E-4	0.777	402.35	0.0024	0.783
338.96	3E-4	2.759	360.66	0.1804	1.016	339.17	3E-4	2.759	360.98	0.1976	1.025
333.58	0.038	1.378	354.52	0.0533	0.855	333.79	0.0395	1.379	354.55	0.0474	0.848
328.86	0.0198	0.969	338.77	2E-4	2.759	328.79	0.0269	1.024	338.95	2E-4	2.753
327.24	0.0464	1.078	333.56	0.0179	1.124	327.24	0.04	1.031	333.93	0.0155	1.073
326.48	3E-4	2.76	331.81	0.0187	0.939	326.61	3E-4	2.76	331.97	0.0223	0.952
311.39	8E-4	0.791	329.99	0.0329	1.149	313.16	6E-4	0.792	330.34	0.0185	1.191
304.12	0.056	2.521	329.56	0.0256	1.113	304.36	0.0596	2.531	330.05	0.0413	1.099
288.99	0.0034	1.973	328.48	0.0052	2.65	291.28	0	1.795	328.91	0.0052	2.664
287.36	0	1.795	326.32	5E-4	2.745	290.68	0.0033	1.922	326.4	5E-4	2.748
283	0.0678	2.123	304.42	0.0512	2.521	284.69	1E-4	0.795	305.33	4E-4	0.804
280.92	1E-4	0.794	303.67	3E-4	0.797	283.34	0.0718	2.112	304.78	0.0548	2.527
277.72	0	0.784	294.82	8E-4	0.848	279.56	0.0026	0.792	294.63	0.0019	1.888
276.45	0.0023	0.794	293.13	0.0029	1.991	275.63	0.0905	1.244	293.58	0.0013	0.914
274.96	0	2.768	290.01	0.0011	1.816	275.03	0	2.769	293.02	5E-4	1.812
274.01	0	2.767	285.38	0.0543	2.103	274.06	0	2.768	285.9	0.0533	2.113
273.68	0.0862	1.166	280.36	2E-4	0.789	273.92	0	0.784	281.21	0.0016	0.827
267.36	0.0467	0.973	277.96	8E-4	0.873	269.43	0	1.708	279.24	0.0276	1.654
266.5	0.0094	0.867	277.42	0.0295	1.537	268.61	1E-4	0.791	278.69	0.0649	1.369
266.4	0.045	1.164	276.68	0.0615	1.425	266.65	0.0606	1.299	277.32	0.0025	0.803
265.96	0	1.709	274.96	7E-4	1.991	264.87	0.0042	2.698	276.86	0.0016	0.797

Table S16: Atomic coordinates (in Å) for geometry optimised **Acr•** in MeCN solution.

Atom	X	Y	Z	Atom	X	Y	Z
**C**	0.09874	5.89307	-1.25448	**C**	-3.61143	-0.64176	1.21604
**H**	-0.99146	5.92843	-1.2819	**C**	2.38828	-4.61236	-6.8E-4
**H**	0.4453	5.39462	-2.163	**C**	-0.03802	-3.74274	-6E-5
**H**	0.46928	6.92138	-1.26747	**H**	-2.00876	-2.91134	6.9E-4
**C**	2.1441	5.21864	-7.6E-4	**H**	5.31004	0.91688	-2.14692
**H**	2.5673	4.73926	-0.88608	**C**	5.46504	0.94309	-0.00167
**H**	2.46658	6.26189	-9.1E-4	**H**	5.31178	0.9168	2.1437
**C**	0.61145	5.16066	6.4E-4	**C**	-2.88476	-0.5077	-2.52461
**H**	2.56894	4.73902	0.88365	**C**	-4.98685	-0.87616	-1.19551
**C**	0.08087	3.7247	7.6E-4	**C**	-4.98715	-0.88124	1.1971
**C**	0.10102	5.89235	1.25711	**C**	-2.88314	-0.51791	2.52761
**C**	0.93096	2.61971	3E-4	**C**	1.75195	-6.00755	-0.00112
**C**	-1.30313	3.48585	0.00151	**C**	3.27326	-4.48352	-1.25576
**H**	0.44923	5.39337	2.16471	**C**	3.27309	-4.48421	1.25461
**H**	0.47156	6.92066	1.27003	**H**	-0.41408	-4.7557	-2E-4
**H**	-0.98913	5.92765	1.2865	**H**	6.53201	1.12845	-0.0021
**C**	0.44135	1.31037	4.6E-4	**H**	-2.09892	-1.26155	-2.61693
**H**	1.99783	2.76708	-2.2E-4	**H**	-3.57043	-0.62641	-3.3641
**C**	-1.80516	2.20012	0.00169	**H**	-2.40143	0.46835	-2.61456
**H**	-1.99554	4.31768	0.00193	**H**	-5.51785	-0.96696	-2.13751
**N**	1.3168	0.22223	1E-5	**C**	-5.69258	-1.0013	0.00227
**C**	-0.95969	1.06763	0.00121	**H**	-5.51714	-0.97589	2.1388
**H**	-2.87527	2.0403	0.00232	**H**	-2.09938	-1.27406	2.619
**C**	0.86519	-1.09992	9E-5	**H**	-2.39752	0.45665	2.62133
**C**	2.73244	0.46926	-5.7E-4	**H**	-3.57015	-0.63744	3.3659
**C**	-1.45556	-0.26353	0.00113	**H**	1.13313	-6.17005	-0.88611
**C**	1.76427	-2.16336	-2.9E-4	**H**	2.53907	-6.76448	-0.0014
**C**	-0.53865	-1.34868	7.1E-4	**H**	1.13319	-6.17066	0.88379
**C**	3.40862	0.58657	-1.20919	**H**	4.02316	-5.27863	-1.27035
**C**	3.4096	0.58652	1.2075	**H**	3.79766	-3.52695	-1.28111
**C**	-2.92011	-0.51885	0.00214	**H**	2.67232	-4.56522	-2.16468
**C**	1.33903	-3.49722	-3.8E-4	**H**	2.67201	-4.56639	2.16339
**H**	2.82146	-1.94784	-5.7E-4	**H**	4.02297	-5.27935	1.26888
**C**	-0.9481	-2.69765	3.7E-4	**H**	3.79748	-3.52766	1.28052
**H**	2.86026	0.49128	-2.13779	**C**	-7.17636	-1.25737	-0.00566
**C**	4.77915	0.82437	-1.20768	**H**	-7.41526	-2.17802	-0.54488
**H**	2.862	0.4912	2.13655	**H**	-7.7138	-0.44556	-0.50355
**C**	4.78013	0.82433	1.2049	**H**	-7.56701	-1.34829	1.00865
**C**	-3.61438	-0.63722	-1.21439				

Table S17: Atomic coordinates (in Å) for geometry optimised [**Acr**$^{•}$·PhCl] in MeCN solution.

**Atom**	**X**	**Y**	**Z**	**Atom**	**X**	**Y**	**Z**
**C**	0.8756	5.16174	-2.3827	**H**	5.2656	0.9285	0.23081
**H**	0.10295	5.01355	-3.14101	**C**	-3.53355	-0.71503	-2.66029
**H**	1.66701	4.43123	-2.55803	**C**	-5.46316	-1.00642	-1.07616
**H**	1.30141	6.15903	-2.52023	**C**	-5.18182	-0.89733	1.29623
**C**	1.4068	5.23817	0.06967	**C**	-2.94051	-0.48684	2.35416
**H**	2.20575	4.50423	-0.04685	**C**	1.38522	-6.09283	-0.55883
**H**	1.8439	6.23312	-0.04792	**C**	2.68822	-4.66129	-2.12775
**C**	0.28485	5.02774	-0.96561	**C**	3.05817	-4.48269	0.34949
**H**	1.01721	5.15535	1.08672	**H**	-0.75189	-4.83262	-0.21192
**C**	-0.31277	3.62832	-0.79476	**H**	5.98203	0.96872	-2.14253
**C**	-0.76089	6.13248	-0.77218	**H**	-2.77466	-1.48669	-2.81212
**C**	0.51968	2.51232	-0.93232	**H**	-4.31749	-0.85797	-3.40467
**C**	-1.66285	3.40265	-0.50509	**H**	-3.05172	0.24672	-2.85254
**H**	-1.19766	6.1049	0.22838	**H**	-6.09935	-1.14334	-1.94415
**H**	-0.28631	7.10742	-0.90191	**C**	-6.02393	-1.06872	0.1984
**H**	-1.57028	6.0564	-1.50132	**H**	-5.59643	-0.94871	2.2975
**C**	0.04535	1.21177	-0.78407	**H**	-2.43442	0.48112	2.34053
**H**	1.5651	2.65858	-1.15453	**H**	-3.52773	-0.54977	3.27083
**C**	-2.15534	2.11554	-0.36197	**H**	-2.16393	-1.25406	2.39508
**H**	-2.34491	4.23268	-0.38971	**H**	0.63414	-6.3111	-1.32113
**N**	0.9008	0.11757	-0.92376	**H**	2.16924	-6.84905	-0.63616
**C**	-1.33068	0.97959	-0.49253	**H**	0.91759	-6.1982	0.42229
**H**	-3.20375	1.96553	-0.14084	**H**	3.43483	-5.45647	-2.1976
**C**	0.46455	-1.19694	-0.75509	**H**	3.19505	-3.71019	-2.2979
**C**	2.28035	0.35272	-1.25202	**H**	1.96036	-4.80604	-2.92978
**C**	-1.81674	-0.34822	-0.35249	**H**	2.59952	-4.51504	1.34024
**C**	1.35411	-2.26243	-0.85837	**H**	3.81908	-5.26606	0.30074
**C**	-0.90873	-1.43593	-0.45806	**H**	3.55768	-3.51927	0.23772
**C**	2.67144	0.37518	-2.58559	**C**	-7.5021	-1.28518	0.38482
**C**	3.20703	0.55195	-0.23609	**H**	-7.93523	-1.81803	-0.4633
**C**	-3.26918	-0.59804	-0.1486	**H**	-8.02778	-0.32913	0.47405
**C**	0.95271	-3.5879	-0.66399	**H**	-7.70911	-1.85466	1.29286
**H**	2.39003	-2.05194	-1.07302	**C**	2.72214	-1.57315	2.99471
**C**	-1.29769	-2.77867	-0.27606	**C**	3.39025	-0.47615	3.53171
**H**	1.93178	0.21837	-3.36049	**C**	2.77381	0.77133	3.58013
**C**	4.00704	0.59766	-2.90495	**C**	1.48497	0.89399	3.07963
**H**	2.88059	0.53204	0.79296	**C**	0.80315	-0.18246	2.53324
**C**	4.54127	0.774	-0.55927	**C**	1.43074	-1.42379	2.49942
**C**	-4.10104	-0.77337	-1.26705	**H**	3.20884	-2.53897	2.95645
**C**	-3.81548	-0.66253	1.14316	**H**	4.39731	-0.58405	3.91468
**C**	2.00001	-4.70123	-0.75	**H**	3.28726	1.62798	3.995
**C**	-0.39458	-3.82531	-0.3707	**H**	-0.18898	-0.05532	2.12918
**H**	-2.33529	-2.98572	-0.04972	**H**	0.90956	-2.26702	2.06717
**H**	4.31631	0.61534	-3.94255	**Cl**	0.69045	2.47761	3.12451
**C**	4.9425	0.79632	-1.89249				

Table S18: Atomic coordinates (in Å) for geometry optimised **Acr•** in THF solution.

Atom	X	Y	Z	Atom	X	Y	Z
C	0.09898	5.89289	-1.25476	C	-3.61123	-0.64144	1.21597
H	-0.99113	5.92858	-1.28363	C	2.38746	-4.61205	-6.8E-4
H	0.44456	5.39376	-2.16308	C	-0.03848	-3.74187	3.7E-4
H	0.46896	6.92146	-1.26947	H	-2.00906	-2.91015	0.00141
C	2.14396	5.21884	-7.9E-4	H	5.30931	0.91529	-2.14727
H	2.56766	4.73985	-0.88603	C	5.46465	0.94148	-0.00236
H	2.46791	6.26168	-0.00101	H	5.31191	0.91539	2.14273
C	0.61123	5.16071	6.7E-4	C	-2.88282	-0.50697	-2.52363
H	2.56937	4.7397	0.88355	C	-4.98567	-0.87596	-1.19541
C	0.08074	3.72466	9.6E-4	C	-4.98654	-0.88121	1.19649
C	0.10137	5.89246	1.25733	C	-2.88235	-0.51743	2.52708
C	0.93075	2.61989	4.4E-4	C	1.75103	-6.00722	-8.6E-4
C	-1.30276	3.48551	0.00189	C	3.27179	-4.48371	-1.25625
H	0.44869	5.39301	2.16482	C	3.27233	-4.48427	1.25459
H	0.47136	6.92103	1.27169	H	-0.41504	-4.7546	3.2E-4
H	-0.98868	5.92811	1.28829	H	6.5317	1.1263	-0.00302
C	0.4412	1.31089	7.1E-4	H	-2.09678	-1.26058	-2.61539
H	1.99761	2.76694	-2.3E-4	H	-3.56718	-0.62564	-3.36431
C	-1.80494	2.20024	0.0022	H	-2.39952	0.46907	-2.6132
H	-1.99564	4.31694	0.00238	H	-5.51667	-0.96689	-2.13746
N	1.31654	0.22237	1.7E-4	C	-5.69142	-1.00146	0.00184
C	-0.95956	1.06805	0.00164	H	-5.51681	-0.97614	2.13802
H	-2.87496	2.04003	0.00303	H	-2.09837	-1.2733	2.61816
C	0.86489	-1.10003	3.5E-4	H	-2.3969	0.45716	2.62071
C	2.73163	0.46902	-6.9E-4	H	-3.56841	-0.63706	3.36625
C	-1.45528	-0.26309	0.00162	H	1.13223	-6.1702	-0.88572
C	1.76357	-2.16333	-1.7E-4	H	2.53692	-6.76546	-0.00125
C	-0.53869	-1.34844	0.00117	H	1.13269	-6.17065	0.88425
C	3.40809	0.58602	-1.20914	H	4.0221	-5.2785	-1.27272
C	3.40956	0.58608	1.20692	H	3.7965	-3.5274	-1.28318
C	-2.9198	-0.51835	0.00237	H	2.67037	-4.56432	-2.16478
C	1.33826	-3.49687	-1.7E-4	H	2.6713	-4.56531	2.16333
H	2.82057	-1.94721	-6.3E-4	H	4.02267	-5.27905	1.27036
C	-0.94832	-2.69706	9.2E-4	H	3.79702	-3.52795	1.28171
H	2.85897	0.4907	-2.13723	C	-7.17501	-1.25821	-0.00682
C	4.77853	0.82309	-1.20795	H	-7.41374	-2.17917	-0.54563
H	2.86157	0.4908	2.13569	H	-7.71321	-0.44644	-0.50403
C	4.78	0.82315	1.20406	H	-7.56686	-1.34976	1.007
C	-3.61361	-0.63675	-1.2142				

Table S19: Atomic coordinates (in Å) for geometry optimised [**Acr**$^{\bullet}$·PhCl] in THF solution.

Atom	X	Y	Z	Atom	X	Y	Z
**C**	1.0081	5.12323	-2.40051	**H**	5.2953	0.75856	0.22818
**H**	0.2267	4.99503	-3.15332	**C**	-3.54376	-0.6622	-2.657
**H**	1.77662	4.36982	-2.58082	**C**	-5.4841	-0.88128	-1.07398
**H**	1.46189	6.10733	-2.54443	**C**	-5.20544	-0.74312	1.29654
**C**	1.55712	5.18974	0.04868	**C**	-2.95678	-0.37334	2.35375
**H**	2.3359	4.43497	-0.07039	**C**	1.21739	-6.13669	-0.54358
**H**	2.02068	6.17237	-0.07219	**C**	2.56826	-4.74083	-2.10342
**C**	0.42357	5.00836	-0.97918	**C**	2.92972	-4.57564	0.37631
**H**	1.17245	5.11758	1.06805	**H**	-0.88328	-4.81632	-0.19461
**C**	-0.21202	3.6264	-0.80334	**H**	6.006	0.79421	-2.14676
**C**	-0.58957	6.14224	-0.78099	**H**	-2.80721	-1.45737	-2.79669
**C**	0.59004	2.48828	-0.93451	**H**	-4.32909	-0.79211	-3.40245
**C**	-1.56847	3.43837	-0.51835	**H**	-3.03388	0.28263	-2.8601
**H**	-1.02166	6.12743	0.22184	**H**	-6.1219	-1.01446	-1.94135
**H**	-0.08959	7.10413	-0.9131	**C**	-6.04899	-0.90979	0.19949
**H**	-1.40443	6.0897	-1.50605	**H**	-5.6236	-0.76831	2.29733
**C**	0.0798	1.20207	-0.78525	**H**	-2.43357	0.58532	2.3317
**H**	1.64004	2.60484	-1.1521	**H**	-3.54546	-0.41538	3.27074
**C**	-2.09641	2.1663	-0.37302	**H**	-2.19543	-1.15501	2.40486
**H**	-2.22812	4.28687	-0.40774	**H**	0.46464	-6.33289	-1.31014
**N**	0.90566	0.08392	-0.91935	**H**	1.97908	-6.91574	-0.6179
**C**	-1.30242	1.00836	-0.49669	**H**	0.74201	-6.23116	0.43488
**H**	-3.14921	2.04512	-0.15526	**H**	3.2908	-5.55806	-2.17268
**C**	0.43333	-1.21755	-0.74779	**H**	3.10509	-3.8058	-2.27013
**C**	2.29047	0.27998	-1.24903	**H**	1.84055	-4.86055	-2.90947
**C**	-1.82432	-0.30535	-0.35133	**H**	2.46509	-4.60295	1.36444
**C**	1.29323	-2.30738	-0.84557	**H**	3.67266	-5.37622	0.32832
**C**	-0.94631	-1.41781	-0.45176	**H**	3.45256	-3.62367	0.27357
**C**	2.67889	0.29924	-2.58346	**C**	-7.53253	-1.08517	0.38577
**C**	3.22568	0.44489	-0.23478	**H**	-7.98053	-1.60719	-0.46137
**C**	-3.28301	-0.51517	-0.14741	**H**	-8.03214	-0.11521	0.4745
**C**	0.85551	-3.62058	-0.64745	**H**	-7.75598	-1.64886	1.29363
**H**	2.33441	-2.12552	-1.0601	**C**	2.63744	-1.62577	3.011
**C**	-1.37199	-2.74863	-0.265	**C**	3.3486	-0.55536	3.54562
**H**	1.93225	0.16891	-3.35652	**C**	2.78794	0.71842	3.57813
**C**	4.01922	0.48486	-2.90543	**C**	1.51035	0.8954	3.06495
**H**	2.90171	0.4266	0.79497	**C**	0.78712	-0.15494	2.52076
**C**	4.56468	0.63018	-0.56077	**C**	1.35888	-1.42298	2.50215
**C**	-4.11642	-0.68595	-1.26508	**H**	3.08066	-2.61266	2.98517
**C**	-3.8336	-0.54566	1.14363	**H**	4.34593	-0.70504	3.93997
**C**	1.87195	-4.7626	-0.7294	**H**	3.33426	1.55507	3.99182
**C**	-0.49781	-3.81973	-0.35488	**H**	-0.19454	0.0135	2.10638
**H**	-2.41546	-2.92601	-0.04056	**H**	0.80493	-2.24559	2.07105
**H**	4.32599	0.50022	-3.9438	**Cl**	0.78714	2.51105	3.09033
**C**	4.96267	0.64995	-1.89471				

Table S20: Atomic coordinates (in Å) for geometry optimised **AcrH** in MeCN solution.

Atom	X	Y	Z	Atom	X	Y	Z
**C**	1.03322	5.32827	0.98557	**H**	6.31153	-3.12876	-1.10381
**C**	1.00828	5.22215	-0.39881	**H**	3.50462	-4.1819	-0.52849
**C**	0.77106	4.00491	-1.04077	**H**	4.94067	-4.44518	0.45969
**C**	0.54883	2.85087	-0.27521	**H**	3.47412	-3.80592	1.19998
**C**	0.56714	2.94039	1.12988	**H**	4.82969	-1.83285	2.10275
**C**	0.80725	4.1729	1.72977	**H**	6.26309	-2.58561	1.38581
**C**	0.28772	1.51621	-0.96523	**H**	5.87077	-0.90203	1.0255
**C**	1.37423	0.49551	-0.68141	**H**	1.87026	-2.77154	0.05289
**C**	1.07748	-0.83917	-0.39121	**H**	4.76527	0.315	-0.5845
**N**	-0.25624	-1.27108	-0.30432	**H**	2.94631	1.91026	-0.94283
**C**	-1.33162	-0.37218	-0.39406	**H**	-2.79727	-1.86092	0.04416
**C**	-1.10433	0.98077	-0.68347	**H**	-0.64064	-3.27031	-1.98861
**C**	2.71261	0.87216	-0.7399	**H**	-0.45796	-2.24952	2.14809
**C**	3.74172	-0.03022	-0.52772	**H**	-2.02654	2.88068	-0.94176
**C**	3.4575	-1.36873	-0.23889	**H**	-1.1092	-5.64169	-1.39384
**C**	2.12309	-1.74991	-0.17401	**H**	-0.92525	-4.61395	2.76462
**C**	-2.19985	1.83028	-0.74146	**H**	-4.31179	2.09076	-0.58964
**C**	-3.49921	1.38225	-0.53183	**H**	-1.25032	-6.30719	0.98689
**C**	-3.73736	0.03913	-0.24718	**H**	-0.2014	3.64656	-2.94911
**C**	-2.63973	-0.81802	-0.18219	**H**	0.9507	4.97818	-2.94689
**C**	4.599	-2.36244	1.13E-4	**H**	1.52617	3.31415	-2.95588
**C**	5.44165	-1.88907	1.20032	**H**	1.17596	6.10814	-1.00146
**C**	-0.52301	-2.63208	0.04603	**H**	0.81644	4.23126	2.81308
**C**	-0.70556	-3.58041	-0.9538	**H**	-0.64927	1.29921	1.82868
**C**	-0.96729	-4.9036	-0.61454	**H**	1.07287	0.9618	1.82959
**C**	-1.04656	-5.27709	0.72322	**H**	0.38663	2.02625	3.06223
**C**	-0.86385	-4.3257	1.72279	**H**	-6.2504	1.00471	-1.09365
**C**	-0.6021	-3.00332	1.38485	**H**	-7.20456	0.10059	0.08139
**C**	-5.13926	-0.52809	7.69E-4	**H**	-6.08054	1.34079	0.63566
**C**	-5.2006	-1.14235	1.4129	**H**	-6.44086	-2.03174	-0.88445
**C**	0.76019	3.98055	-2.55141	**H**	-4.72629	-2.44134	-0.98478
**C**	0.33134	1.74111	2.01156	**H**	-5.39706	-1.20929	-2.05464
**C**	1.31797	6.64167	1.66502	**H**	-4.98248	-0.38777	2.17123
**C**	5.4889	-2.42564	-1.25628	**H**	-6.19709	-1.54643	1.60789
**C**	4.09077	-3.77898	0.29959	**H**	-4.48083	-1.95322	1.53035
**C**	-6.22639	0.54941	-0.10187	**H**	1.18316	7.47975	0.98007
**C**	-5.44123	-1.61994	-1.04401	**H**	2.34737	6.67798	2.03345
**H**	5.91867	-1.45199	-1.49346	**H**	0.66079	6.79558	2.52327
**H**	4.91127	-2.75649	-2.12165	**H**	0.32492	1.70187	-2.04036

Table S21: Atomic coordinates (in Å) for geometry optimised **AcrH** in THF solution.

Atom	X	Y	Z	Atom	X	Y	Z
**C**	0	0	0	**H**	7.29728	-6.70994	2.63018
**C**	0	0	1.39014	**H**	8.76659	-4.1768	1.78038
**C**	1.1859	0.00136	2.12935	**H**	8.70157	-5.69606	0.88734
**C**	2.42083	0.00427	1.45904	**H**	8.27053	-4.18751	0.08199
**C**	2.43893	0.00718	0.05014	**H**	6.03054	-5.2378	-0.58953
**C**	1.23323	0.0057	-0.64876	**H**	6.58574	-6.72748	0.18836
**C**	3.71921	0.00826	2.267	**H**	5.02322	-6.03057	0.62568
**C**	4.53845	-1.25034	2.05401	**H**	7.61596	-2.35913	1.17546
**C**	5.88328	-1.21066	1.67146	**H**	4.13362	-4.623	2.21267
**N**	6.52924	0.01585	1.45844	**H**	2.90115	-2.53334	2.52712
**C**	5.87983	1.24011	1.67235	**H**	7.60274	2.40474	1.17832
**C**	4.53052	1.27275	2.05596	**H**	8.69858	0.01748	2.98514
**C**	3.94608	-2.49677	2.24161	**H**	7.33415	0.02518	-1.06223
**C**	4.64205	-3.68084	2.05688	**H**	2.88819	2.54358	2.52869
**C**	5.98621	-3.6538	1.6654	**H**	11.05777	0.0275	2.19976
**C**	6.58452	-2.41312	1.48014	**H**	9.68782	0.03498	-1.86544
**C**	3.93345	2.5122	2.24367	**H**	4.10348	4.64018	2.21788
**C**	4.6226	3.70637	2.05941	**H**	11.54765	0.03625	-0.22883
**C**	5.96194	3.68581	1.66907	**H**	1.56083	0.8835	4.07735
**C**	6.56873	2.44389	1.48351	**H**	0.0412	0.00208	3.94514
**C**	6.75107	-4.96495	1.45225	**H**	1.56311	-0.87513	4.07932
**C**	6.05168	-5.78821	0.35405	**H**	-0.94638	6.23E-4	1.92006
**C**	7.88838	0.02014	1.00448	**H**	1.26084	0.01052	-1.73343
**C**	8.92903	0.0211	1.92723	**H**	4.32891	0.8927	-0.51051
**C**	10.24679	0.02688	1.482	**H**	4.33311	-0.86212	-0.52064
**C**	10.5214	0.03194	0.1171	**H**	3.50944	0.02095	-1.80796
**C**	9.47665	0.03118	-0.80325	**H**	5.60418	6.28628	2.71082
**C**	8.1583	0.02539	-0.36002	**H**	6.59298	7.10712	1.50301
**C**	6.78445	4.95814	1.43425	**H**	5.10569	6.29622	1.01259
**C**	7.28519	4.98084	-0.02246	**H**	8.58739	5.87451	2.23488
**C**	1.08683	0.00309	3.63613	**H**	8.64709	4.10969	2.21767
**C**	3.72233	0.01517	-0.73861	**H**	7.66961	4.945	3.42913
**C**	-1.2854	-0.02969	-0.78267	**H**	6.44586	4.96776	-0.72179
**C**	6.75519	-5.77037	2.76532	**H**	7.86838	5.88674	-0.20725
**C**	8.206	-4.73282	1.02594	**H**	7.92099	4.12198	-0.24311
**C**	5.96601	6.23102	1.68197	**H**	-2.13379	0.26473	-0.16296
**C**	7.99526	4.96828	2.38647	**H**	-1.48847	-1.03488	-1.16513
**H**	5.7429	-6.01157	3.09315	**H**	-1.24225	0.64047	-1.64407
**H**	7.24356	-5.20764	3.56441	**H**	3.42908	0.00684	3.31707

Table S22: Atomic coordinates (in Å) for geometry optimised PhCl in MeCN solution.

Atom	X	Y	Z	Atom	X	Y	Z
**C**	1.434522	-1.421878	2.490962	Cl	0.693023	2.481019	3.125978
**C**	2.724666	-1.572274	2.990854	H	3.209565	-2.539699	2.959111
**C**	3.39025	-0.476146	3.531859	H	4.394317	-0.58552	3.922153
**C**	2.774235	0.771417	3.577358	H	3.286559	1.626674	3.996599
**C**	1.487354	0.896349	3.073011	H	-0.198888	-0.058575	2.14109
**C**	0.803539	-0.181379	2.528297	H	0.91035	-2.269994	2.068549

Table S23: Atomic coordinates (in Å) for geometry optimised PhCl in THF solution.

Atom	X	Y	Z	Atom	X	Y	Z
**C**	1.434798	-1.421657	2.491109	Cl	0.693343	2.48042	3.125938
**C**	2.724624	-1.57218	2.990849	H	3.209522	-2.539596	2.9591
**C**	3.389908	-0.476225	3.531706	H	4.393969	-0.585753	3.921977
**C**	2.773926	0.770975	3.577124	H	3.285664	1.62657	3.996208
**C**	1.487198	0.896671	3.073012	H	-0.198285	-0.057948	2.141539
**C**	0.804078	-0.181409	2.528516	H	0.910746	-2.269877	2.068743

Table S24: Atomic coordinates (in Å) for geometry optimised MeCN in MeCN solution.

Atom	X	Y	Z
**C**	-3.516989	0.490599	-0.000059
**C**	-3.516976	-0.95902	-0.000137
**N**	-3.516656	-2.110827	0.000378
**H**	-2.490454	0.85978	0.00015
**H**	-4.030005	0.859819	-0.889154
**H**	-4.0304	0.85965	0.888822

Table S25: Atomic coordinates (in Å) for geometry optimised dehydrogenated MeN radical (MeCN-deH•) in MeCN solution.

Atom	X	Y	Z
**C**	-3.369103	0.389409	-0.256726
**C**	-3.478642	-0.969484	-0.068444
**N**	-3.570289	-2.120877	0.094408
**H**	-2.512853	0.919244	0.138558
**H**	-4.140192	0.922059	-0.796618

Table S26: Atomic coordinates (in Å) for geometry optimised THF in THF solution.

Atom	X	Y	Z	Atom	X	Y	Z
**C**	-3.104341	-0.337198	0.528296	H	-4.027065	0.054078	-1.429927
**C**	-3.271178	0.466188	-0.758292	H	-3.532361	1.508326	-0.540016
**O**	-1.994024	0.429928	-1.425416	H	-0.107287	0.552039	-0.589269
**C**	-0.994297	-0.081136	-0.521714	H	-0.719237	-1.097213	-0.828153
**C**	-1.631814	-0.081529	0.864623	H	-1.200151	-0.837582	1.520817
**H**	-3.78973	-0.014244	1.312182	H	-1.515718	0.895908	1.339149
**H**	-3.270681	-1.400539	0.339445				

Table S27: Atomic coordinates (in Å) for geometry optimised dehydrogenated THF radical (THF-deH•) in THF solution.

Atom	X	Y	Z	Atom	X	Y	Z
**C**	-3.11245	-0.343048	0.528056	H	-4.125818	0.034364	-1.367076
**C**	-3.297689	0.45425	-0.775497	H	-1.712321	0.523456	-2.446091
**C**	-1.976892	0.239014	-1.435522	H	-1.506437	-1.824142	0.858156
**O**	-1.39216	-0.926735	-1.002192	H	-2.750051	-2.32531	-0.309935
**C**	-2.188414	-1.473688	0.084496	H	-4.048597	-0.717832	0.940343
**H**	-3.523818	1.508182	-0.604195	H	-2.621511	0.275667	1.281262

Table S28: Total energy data (in a.u.) computed for B3LYP(D3BJ)/TZVP/SMD optimised structures. $\boldsymbol{E_0}$ and $\boldsymbol{G^0}$ denote electronic energies and Gibbs free energies both including solvent effects estimated via the SMD model.

	Solvent = MeCN		Solvent = THF	
**Molecule**	$\boldsymbol{E_0}$	$\boldsymbol{G^0}$	$\boldsymbol{E_0}$	$\boldsymbol{G^0}$
**Acr**$^{\bullet}$·PhCl	-2143.3813	-2142.7124	-2143.3786	-2142.7083
**Acr**$^{\bullet}$	-1451.3867	-1450.8004	-1451.3847	-1450.7980
PhCl	-691.9795	-691.9189	-691.9788	-691.9181
**Acr**H	-1452.0019	-1451.3988	-1451.9994	-1451.3961
MeCN	-132.8204	-132.7992	-	-
MeCN-deH$^{\bullet}$	-132.1551	-132.1483	-	-
THF	-	-	-232.5562	-232.4682
THF-deH$^{\bullet}$	-	-	-231.8979	-231.8231

9 References

1 I. S. Weitz and M. Rabinovitz, "The application of C_8K for organic synthesis: reduction of substituted naphthalenes" *J. Chem. Soc. Perkin Trans. 1*, **1993**, 117-120.

2 J. Hicks, M. Juckel, A. Paparo, D. Dange and C. Jones, "Multigram Syntheses of Magnesium(I) Compounds Using Alkali Metal Halide Supported Alkali Metals as Dispersible Reducing Agents" *Organometallics*, **2018**, *37*, 4810-4813.

3 S. J. Horsewill, G. Hierlmeier, Z. Farasat, J. P. Barham and D. J. Scott, "Shining Fresh Light on Complex Photoredox Mechanisms through Isolation of Intermediate Radical Anions" *ACS Cat.*, **2023**, *13*, 9392-9403.

4 S.J. Horsewill, C. Cao, N. Dabney, E.S. Yang, S. Faulkner and D.J. Scott, "Isolation of the elusive $[Ru(bipy)_3]^+$: a key intermediate in photoredox catalysis" *Chem. Commun.*, **2023**, *59*, 14665-14668.

5 U. Lennert, P. B. Arockiam, V. Streitferdt, D. J. Scott, C. Rödl, R. M. Gschwind and R. Wolf, "Direct catalytic transformation of white phosphorus into arylphosphines and phosphonium salts" *Nature Catal.* **2019**, *2*, 1101-1106.

6 J. M. Woolley, M. Staniforth, M. D. Horbury, G. W. Richings, M. Wills and V. G. Stavros, "Unravelling the Photoprotection Properties of Mycosporine Amino Acid Motifs", *J. Phys. Chem. Lett.*, **2018**, *9*, 3043–3048.

7 J. M. Woolley, J. S. Peters, M. A. P. Turner, G. J. Clarkson, M. D. Horbury and V. G. Stavros, "The role of symmetric functionalisation on photoisomerization of a UV commercial chemical filter", *Phys. Chem. Chem. Phys.*, **2019**, *21*, 14350–14356.

8 J. J. Snellenburg, S. Laptenok, R. Seger, K. M. Mullen and I. H. M. van Stokkum, "Glotaran: A Java-Based Graphical User Interface for the R Package TIMP" *J. Stat. Soft.* **2012**, *49*, 1-22.

9 K. Tajima, N. Fukui and H. Shinokubo, "Aggregation-Induced Emission of Nitrogen-Bridged Naphthalene Monoimide Dimers", *Org. Lett.*, **2019**, *21*, 9516-9520.

10 D. T. Breslin and M. A. Fox, "Excited-state Behavior of Thermally Stable Radical Ions", *J. Phys. Chem.*, **1994**, *98*, 408-411.

11 A. J. Rieth, M. I. Gonzalez, B. Kudisch, M. Nava and D. G. Nocera, "How Radical Are "Radical" Photocatalysts? A Closed-Shell Meisenheimer Complex Is Identified as a Super-Reducing Photoreagent", *J. Am. Chem. Soc.*, **2021**, *143*, 14352-14359.

12 Viscosity value for MeCN at T = 298 K is averaged from four collated at: https://materials.springer.com/thermophysical/docs/vis_c3%0A#experimental-data-table_wrapper, accessed 28/02/25.

13 B. Pfund and O. S. Wenger, "Excited Organic Radicals in Photoredox Catalysis", *J. Am. Chem. Soc. Au*, **2025**, *5*, 426-447.

14 O. V. Dolomanov, L. J. Bourhis, R. J. Gildea, J. A. K. Howard and H. Puschmann, "Olex2: A complete structure solution, refinement and analysis program", *J. Appl. Cryst.*, **2009**, *42*, 339-341.

15 G. Sheldrick, "Crystal Structure Refinement with SHELXL", *Acta Crystallogr. C*, **2015**, *71*, 3-8.

16 L. Pitzer, F. Sandfort, F. Strieth-Kalthoff and F. Glorius, "Carbonyl-Olefin Cross-Metathesis Through a Visible-Light-Induced 1,3-Diol Formation and Fragmentation Sequence" *Angew. Chem. Intl. Ed.*, **2018**, *57*, 16219-16223.

17 S. Battaglioli, G. Bertuzzi, R. Pedrazzani, J. Benetti, G. Valenti, M. Montalti, M. Monari and M. Bandini, "Visible-Light-Assisted Synthesis of Allylic Triflamides via Dual Acridinium/Co Catalysis" *Adv. Synth. Catal.*, **2022**, *364*, 720-725.

18 M. J. Frisch, G. W. Trucks, H. B. Schlegel, G. E. Scuseria, M. A. Robb, J. R. Cheeseman, G. Scalmani; V. Barone, G. A. Petersson, H. Nakatsuji, X. Li, M. Caricato, A. V. Marenich, J. Bloino, B. G. Janesko, R. Gomperts, B. Mennucci, H. P. Hratchian, J. V. Ortiz, A. F. Izmaylov, J. L. Sonnenberg, D. Williams-Young, F. Ding, F. Lipparini, F. Egidi, J. Goings, B. Peng, A. Petrone, T. Henderson, D. Ranasinghe, V. G. Zakrzewski, J. Gao, N. Rega, G. Zheng, W. Liang, M. Hada, M. Ehara, K. Toyota, R. Fukuda, J. Hasegawa, M. Ishida, T. Nakajima, Y. Honda, O. Kitao, H. Nakai, T. Vreven, K. Throssell, J. A. Montgomery Jr., J. E. Peralta, F. Ogliaro, M. J. Bearpark, J. J. Heyd, E. N. Brothers, K. N. Kudin, V. N. Staroverov, T. A. Keith, R. Kobayashi, J. Normand, K. Raghavachari, A. P. Rendell, J. C. Burant, S. S. Iyengar, J. Tomasi, M. Cossi, J.

M. Millam, M. Klene, C. Adamo, R. Cammi, J. W. Ochterski, R. L. Martin, K. Morokuma, O. Farkas, J. B. Foresman, and D. J. Fox, *Gaussian 16, Revision A.03*; Gaussian, Inc., Wallingford CT, 2016.
19 T. Lu, "A Comprehensive Electron Wavefunction Analysis Toolbox for Chemists, Multiwfn." *J. Chem. Phys.*, **2024**, *161*.
20 S. Grimme, S. Ehrlich and L. Goerigk, "Effect of the Damping Function in Dispersion Corrected Density Functional Theory." *J. Comput. Chem.,* **2011**, *32*, 1456–1465.
21 A. D. Becke, "Density-functional Thermochemistry. III. The Role of Exact Exchange." *J. Chem. Phys.,* **1993**, *98*, 5648–5652.
22 A. Schäfer, C. Huber and R. Ahlrichs "Fully Optimized Contracted Gaussian Basis Sets of Triple Zeta Valence Quality for Atoms Li to Kr." *J. Chem. Phys.,* **1994**, *100*, 5829–5835.
23 A. V. Marenich, C. J. Cramer and D. G Truhlar "Universal Solvation Model Based on Solute Electron Density and on a Continuum Model of the Solvent Defined by the Bulk Dielectric Constant and Atomic Surface Tensions." *J. Phys. Chem. B*, **2009**, *113*, 6378–6396.
24 P. P. Fehér, Á. Madarász and A. Stirling "Multiscale Modeling of Electronic Spectra Including Nuclear Quantum Effects." *J. Chem. Theory Comput.*, **2021**, *17*, 6340–6352.
25 P. P. Fehér, Á. Madarász and A. Stirling "A Practice-Oriented Benchmark Strategy to Predict the UV-Vis Spectra of Organic Photocatalysts." *Chemistry–Methods*, **2023**, *3*.
26 Z. Liu, T. Lu and Q. Chen, "An Sp-Hybridized All-Carboatomic Ring, Cyclo[18]Carbon: Electronic Structure, Electronic Spectrum, and Optical Nonlinearity." *Carbon*, **2020**, *165*, 461–467.
27 F. Neese, "The ORCA Program System." *Wiley Interdiscip. Rev. Com-put. Mol. Sci.,* **2011**, *2*, 73–78.
28 F. Neese "Software Update: The ORCA Program System—Version 5.0." *Wiley Interdiscip. Rev. Comput. Mol. Sci.,* **2022**, *12*, e1606.
29 C. Bannwarth, S. Ehlert and S. Grimme, "GFN2-xTB—An Accurate and Broadly Parametrized Self-Consistent Tight-Binding Quantum Chemical Method with Multipole Electrostatics and Density-Dependent Dispersion Contributions." *J. Chem. Theory Comput.*, **2019**, *15*, 1652–1671.
30 B. de Souza, F. Neese and R. Izsák, "On the Theoretical Prediction of Fluorescence Rates from First Principles Using the Path Integral Approach." *J. Chem. Phys.,* **2018**, *148*.
31 F. Weigend and R. Ahlrichs, "Balanced Basis Sets of Split Valence, Triple Zeta Valence and Quadruple Zeta Valence Quality for H to Rn: Design and Assessment of Accuracy." *Phys. Chem. Chem. Phys.*, **2005**, *7*, 3297.
32 F. Weigend, "Accurate Coulomb-Fitting Basis Sets for H to Rn." *Phys. Chem. Chem. Phys.*, **2006**, *8*, 1057.
33 A. Kumar, P. Malevich, L. Mewes, S. Wu, J. P. Barham and J. Hauer, "Transient absorption spectroscopy based on uncompressed hollow core fiber white light proves pre-association between a radical ion photocatalyst and substrate" *J. Chem. Phys.*, **2023**, *158*, 144201
34 B. Pfund, D. Gejsnæs-Schaad, B. Lazarevski and O. S. Wenger, "Picosecond reactions of excited radical ion super-reductants", *Nat. Commun.*, **2024**, *15*, 4738.
